# High-level expression of leghemoglobin in *Kluyveromyces marxianus* by remodeling the heme metabolism pathway

**DOI:** 10.3389/fbioe.2023.1329016

**Published:** 2024-01-08

**Authors:** Tian Tian, Xinwei Wu, Pingping Wu, Xinyi Lu, Qi Wang, Yifan Lin, Canjie Liu, Jungang Zhou, Yao Yu, Hong Lu

**Affiliations:** ^1^ State Key Laboratory of Genetic Engineering, School of Life Sciences, Fudan University, Shanghai, China; ^2^ Shanghai Engineering Research Center of Industrial Microorganisms, Shanghai, China

**Keywords:** *Kluyveromyces marxianus*, leghemoglobin, heme biosynthesis, yeast artificial chromosomes, high-level expression

## Abstract

Soy leghemoglobin, when bound to heme, imparts a meat-like color and flavor and can serve as a substitute for animal-derived proteins. Enhancing cellular heme synthesis improves the recombinant expression of leghemoglobin in yeast. To achieve high-level expression of leghemoglobin A (LBA) in *Kluyveromyces marxianus*, a food-safe yeast, large-scale heme synthesis modules were transferred into *K. marxianus* using yeast artificial chromosomes (KmYACs). These modules contained up to 8 native and heterologous genes to promote the supply of heme precursors and downstream synthesis. Next, eight genes inhibiting heme or LBA synthesis were individually or combinatorially deleted, with the *lsc1*Δ*ssn3*Δ mutant yielding the best results. Subsequently, heme synthesis modules were combined with the *lsc1*Δ*ssn3*Δ mutant. In the resulting strains, the module genes were all actively expressed. Among these module genes, heterologous *S. cerevisiae* genes in the downstream heme synthesis pathway significantly enhanced the expression of their counterparts in *K. marxianus*, resulting in high heme content and LBA yield. After optimizing the medium recipe by adjusting the concentrations of glucose, glycine, and FeSO_4_·7H_2_O, a heme content of 66.32 mg/L and an intracellular LBA titer of 7.27 g/L were achieved in the engineered strain in a 5 L fermentor. This represents the highest intracellular expression of leghemoglobin in microorganisms to date. The leghemoglobin produced by *K. marxianus* can be utilized as a safe ingredient for plant-based protein products.

## Introduction

Leghemoglobin was discovered in *Glycine max* in 1939 (Hoy and Hargrove, 2008). It is synthesized collaboratively by symbiotic rhizobia bacteria and the host plant in the root nodules, where it plays a crucial role in nitrogen fixation (Jiang et al., 2021). Leghemoglobin has a molecular weight of approximately 150–170 kDa and consists of four major components in the root nodules: leghemoglobin A (LBA), leghemoglobin C1 (LBC1), leghemoglobin C2 (LBC2), and leghemoglobin C3 (LBC3) (Singh and Varma, 2017). Leghemoglobin is composed of a single polypeptide chain and a heme group, similar to the structure of myoglobin found in mammals. The bound heme residues (porphyrin ring and iron ion complex) regulate the oxygen concentration and participate in the nitrogen fixation process of the rhizobia bacteria (Jiang et al., 2021). Due to the structural and functional similarity between leghemoglobin and myoglobin, leghemoglobin can produce meat-like color and flavor, making it a potential substitute for animal-derived proteins (Ismail, Hwang, and Joo, 2020).

Using microbial cell factories for leghemoglobin production offers several advantages, including industrial scalability, low cost, and short production cycles. As a result, it proves more appealing than extracting leghemoglobin from plant root nodules. Currently, secretory expression of leghemoglobin (LBC2) has only been achieved in *Pichia pastoris*, with a titer of 3.5 g/L (Shao et al., 2022). Intracellular expression of leghemoglobin (LBC2) in *Saccharomyces cerevisiae* achieved a level of 108 mg/L (Xue et al., 2023), and in *Escherichia coli*, intracellular expression of leghemoglobin II (LbII) reached 0.23 mg/g DCW (Arredondo-Peter et al., 1997). Different strategies have been applied to enhance hemoglobin expression in microorganisms. For instance, the deletion of *VPS10* and *PEP4*, which are involved in the degradation of misfolded proteins in the vacuole, has been employed to reduce hemoglobin degradation, thereby increasing the expression of human hemoglobin (Ishchuk et al., 2021). By using a strong promoter and increasing the copy number of the hemoglobin expression cassette, the expression of leghemoglobin was significantly improved (Shao et al., 2022). Another successful strategy to promote hemoglobin production is to enhance cellular heme synthesis. Heme binds to the nascent globin chains to promote the co-translational folding of the growing polypeptide (Komar et al., 1997). The supplementation of heme significantly contributed to the solubility and half-life of recombinant human hemoglobin expressed in *E. coli* (Weickert and Curry, 1997). In *S. cerevisiae*, a five-fold increase in bound heme levels was achieved by eliminating spatial barriers in the heme synthesis process, enhancing the synthesis of heme precursors, and moderately improving the rate-limiting steps of heme synthesis. Consequently, the engineered strains exhibited a 46% increase in the expression of soybean hemoglobin (Liu et al., 2014). In *P. pastoris*, the overexpression of all heme biosynthetic pathway genes resulted in a nine-fold increase in the secretion of active soybean hemoglobin H (Shao et al., 2022).

Heme is widely synthesized in animals, plants, and microorganisms. The precursor for heme synthesis is 5-aminolevulinic acid (5-ALA). Depending on the species, the synthesis of 5-ALA can occur via the C5 pathway or the C4 pathway (Su et al., 2023). Most bacteria use the C5 pathway, which involves the catalysis of glutamyl-tRNA synthetase (GltX), glutamyl-tRNA reductase (HemA), and glutamate-1-semialdehyde aminotransferase (HemL) to convert L-glutamate into 5-ALA (Yang et al., 2016). In eukaryotes, the C4 pathway is utilized, where 5-ALA is synthesized from glycine and succinyl-CoA by ALA synthase (Hem1). The conversion of 5-ALA to heme requires seven downstream enzymes: porphobilinogen synthase (Hem2), porphobilinogen deaminase (Hem3), uroporphyrinogen III synthase (Hem4), uroporphyrinogen III decarboxylase (Hem12), coproporphyrinogen III oxidase (Hem13), protoporphyrinogen oxidase (Hem14), and ferrochelatase (Hem15) (Kornitzer and Roy, 2020). Different metabolic engineering strategies have been applied to improve the amount of cellular heme. For example, improving the supply of precursors for heme synthesis increases heme production. In *Corynebacterium glutamicum*, the metabolic flux of heme precursor synthesis was enhanced by overexpressing *Rhodobacter capsulatus alaS* in the C4 pathway, *Salmonella typhimurium hemA* ^M^ and *E. coli hemL* in the C5 pathway. The synthesis of 5-ALA was increased by at least 5.6-fold, resulting in a 4.49-fold increase in heme content (Ko et al., 2021). Supplementation of L-glutamate, a precursor in the C5 pathway, resulted in an increase in the total heme content from 115.5 mg/L to 239.3 mg/L (Zhao, Choi, and Lee, 2018). Heme production can also be improved by alleviating the repression of key heme enzymes in cells. In *S. cerevisiae*, the transcription factor Rox1 inhibits the expression of *HEM13* under high heme conditions (Keng, 1992), and *HAP1* activates the transcription of *ROX1* (Martínez et al., 2015). The protein complex encoded by *SSN6* and *TUP1* inhibits the repressive regulatory genes of *ROX1* (Amillet, Buisson, and Labbe-Bois, 1995). Deletion of *ROX1*, *HAP1*, *SSN6* or *TUP1* significantly reduces the inhibition of *HEM13* in aerobic cells, thereby increasing cellular heme synthesis (Kastaniotis and Zitomer, 2000). Combined strategies were applied to achieve the highest heme content in several microbes. For instance, in *E. coli*, the overexpression of enzymes in the downstream pathway, along with the heme transporter CcmABC, and the elimination of heme degradation and competitive pathway genes, yielded a total heme titer of 115.5 mg/L and an extracellular heme titer of 73.4 mg/L (Zhao, Choi, and Lee, 2018). In *C. glutamicum*, the combination of C4 and C5 pathways increased the carbon flux toward heme and porphyrin intermediate biosynthesis, resulting in a total heme titer of 309 mg/L and an extracellular heme titer of 242 mg/L (Ko et al., 2021). In *S. cerevisiae*, eleven genes involved in heme biosynthesis, Fe(II) transportation, serine and glycine metabolism were modified, resulting in an intracellular heme titer of 53.5 mg/L, which was 70 times higher than the titer in the control strain (Ishchuk et al., 2022).

*Kluyveromyces marxianus* (*K. marxianus*) is a food-grade yeast certified by the U.S. Food and Drug Administration and the European Union Food Safety Authority (Lane and Morrissey, 2010). *K. marxianus* possesses various advantageous properties for industrial production, including thermotolerance (Raimondi et al., 2013), high biomass production, fast growth rate, and a broad spectrum of utilizable carbon sources, which make it a promising microbial cell factory for producing heterologous proteins, especially food-grade proteins. In this study, *K. marxianus* was applied in the production of leghemoglobin (LBA) for the first time. Yeast artificial chromosomes (KM-YACs) were used to introduce designed heme synthesis modules into *K. marxianus*, and genes affecting heme accumulation were deleted, resulting in the remodeling of the heme synthesis pathway. The crosstalk between heterologous and native heme synthesis genes was investigated. In an engineered strain, KM_LSC4D_, the relative level of heme and leghemoglobin increased by 6.57 times and 113%, respectively. After optimization of the media recipe, the content of heme in KM_LSC4D_ reached 66.32 mg/L, and the titer of LBA reached 7.27 g/L in a 5 L fermentor, which was the highest intracellular expression of leghemoglobin in microorganisms so far. The LBA expressed by food-grade *K. marxianus* provides a safe ingredient for substitutes of animal-based proteins.

## Materials and methods

### Plasmids and strains

Plasmids used in this study were listed in Supplementary Table S1. To construct a LBA-expressing plasmid, ORF of *LBA* (gene ID:100527427) was inserted between the *Not* I and *Spe* I sites of pUKDN115 (Duan et al., 2019). The resulting plasmid was named pLBA. A His_6_-tag was fused to the C-terminus of LBA in pLBA to obtain pLBA-His_6_. To construct a plasmid carrying a single-gene cassette of *HEM1*_*Km*_, ORF of *HEM1* (gene ID: 34715801), promoter and terminator of *ADH1* (gene ID:34716095) were amplified from the genome of FIM1ΔU, and ligated with pMD18-T vector (6,011, Takara, China) by Seamless Assembly Mix (RK21020, ABclonal, China). The resulting plasmid was named LHZ1081. Similarly, plasmids carrying other single-gene cassettes were constructed. ORF used in the construction included *HEM2*_*Km*_ (gene ID: 34714765), *HEM3*_*Sc*_ (gene ID: 851322), *HEM4*_*Km*_ (gene ID: 34713986), *HEM12*_*Sc*_ (gene ID: 851617), *HEM13*_*Km*_ (gene ID: 34716108), *HEM14*_*Sc*_ (gene ID: 856733), *HEM15*_*Sc*_ (gene ID: 854347), *GLTX*_*Km*_ (gene ID: 34715977), *HEMA*_*Sty*_ (gene ID: NC_003197.2), and *HEML*_*Eco*_ (gene ID: 946892). Promoter and terminator of *PDC1* (gene ID: 34717303), *PGK1* (gene ID: 34714197), *ENO2* (gene ID: 34714125), *INU1* (gene ID: 34714176), *AFT1* (gene ID: 8197809), *TEF* (gene ID: 4619833), *OM45* (gene ID: 34716359), *FBA1* (gene ID: 34716374) and *HXT4* (gene ID: 34716967) were used in the construction. The resulting plasmids were named LHZ1110∼LHZ1119. To construct a plasmid carrying a multiple-gene cassette of *HEM2*_*Km*_-*HEM3*_*Sc*_, *HEM2*_*Km*_ cassette was released from LHZ1110 by *Sac* II digestion, and *HEM3*_*Sc*_ cassette was released from LHZ1111 by *Swa* I and *Sac* I digestion. Two released cassettes were ligated together by Seamless Assembly Mix and then ligated with pMD18-T vector. The resulting plasmid was named LHZ1083. Similarly, a plasmid carrying cassette of *HEMA*_*Sty*_-*HEML*_*Eco*_-*GLTX*_*Km*_-*HEM1*_*Km*_ was constructed and named LHZ1082. A plasmid carrying cassette of *HEM4*_*Km*_-*HEM12*_*Sc*_-*HEM13*_*Km*_-*HEM14*_*Sc*_-*HEM15*_*Sc*_, was constructed and named LHZ1084. Sequences of single-gene and multiple-gene cassettes are listed in Supplementary Table S2. To construct CRISPR plasmids (LHZ1128∼1135) used in deleting genes, primers containing 20 bp target sequences were annealed in pairs and inserted into *Sap* I or *Aar* I sites of LHZ531 (Shi et al., 2021). Primers used in the construction are listed in Supplementary Table S3.

The strains used in this study are listed in Table 1. All *K. marxianus* strains originated from FIM-1 (China General Microbiological Culture Collection Center, CGMCC No.10621) (Zhou et al., 2018). To delete *LSC1* (gene ID: 34715035), 500 bp sequences upstream and downstream of *LSC1* ORF were amplified and ligated together as the donor sequence. CRISPR plasmid LHZ1128 was co-transformed with the donor sequence into T1a by a lithium acetate method to obtain a *LSC1* deletion mutant (KM_L1_) (Iborra, 1993). Using the same strategy, *LSC2* (gene ID: 34714005), *ROX1* (gene ID: 34718061), *HAP1* (gene ID: 34716284), *SSN3* (gene ID: 34717610), *TUP1* (gene ID: 34714724), *VPS10* (gene ID: 34714525) and *PEP4* (gene ID: 34717789) were individually deleted in T1a to obtain KM_L2_, KM_R1_, KM_H1_, KM_S3_, KM_T1_, KM_V10_, and KM_P4_, respectively. *SSN3* was deleted in KM_L1_ to obtain KM_LS_. *PEP4* was deleted in KM_L1_ to obtain KM_LP_. *ROX1* was deleted in KM_LS_ to obtain KM_LSR_. *TUP1* was deleted in KM_LS_ to obtain KM_LST_. *VPS10* was deleted in KM_LP_ to obtain KM_LPV_. *ROX1* was deleted in KM_LPV_ to obtain KM_LPVR_. *SSN3* was deleted in KM_LPV_ to obtain KM_LPVS_. *TUP1* was deleted in KM_LPVS_ to obtain KM_LPVST_. *HIS3* (gene ID:34716654) and *TRP1* (gene ID:34717357) were deleted in FIM1ΔU to obtain KM_HW_. *HIS3* and *TRP1* were deleted in KM_LS_ to obtain KM_LSHW_. Construction of strains containing KmYACs was described below.

**TABLE 1:** Strains used in this study.

Name	Genotype	Source
FIM1ΔU	*ura3*Δ	Zhou et al. (2018)
T1a	*ura3*Δ *atg1*Δ	Liu et al. (2018)
KM_ΔHW_	*ura3*Δ *his3*Δ *trp1*Δ	This study
KM_C4D_	*ura3*Δ *his3*Δ *trp1*Δ KmYAC [C4+Down]	This study
KM_C45D_	*ura3*Δ *his3*Δ *trp1*Δ KmYAC [C4+C5+Down]	This study
KM_L1_	*ura3*Δ *atg1*Δ *lsc1*Δ	This study
KM_L2_	*ura3*Δ *atg1*Δ *lsc2*Δ	This study
KM_R1_	*ura3*Δ *atg1*Δ *rox1*Δ	This study
KM_H1_	*ura3*Δ *atg1*Δ *hap1*Δ	This study
KM_S3_	*ura3*Δ *atg1*Δ *ssn3*Δ	This study
KM_T1_	*ura3*Δ *atg1*Δ *tup1*Δ	This study
KM_V10_	*ura3*Δ *atg1*Δ *vps10*Δ	This study
KM_P4_	*ura3*Δ *atg1*Δ *pep4*Δ	This study
KM_LS_	*ura3*Δ *atg1*Δ *lsc1*Δ *ssn3*Δ	This study
KM_LP_	*ura3*Δ *atg1*Δ *lsc1*Δ *pep4*Δ	This study
KM_LSR_	*ura3*Δ *atg1*Δ *lsc1*Δ *ssn3*Δ *rox1*Δ	This study
KM_LST_	*ura3*Δ *atg1*Δ *lsc1*Δ *ssn3*Δ *tup1*Δ	This study
KM_LPV_	*ura3*Δ *atg1*Δ *lsc1*Δ *pep4*Δ *vps10*Δ	This study
KM_LPVR_	*ura3*Δ *atg1*Δ *lsc1*Δ *pep4*Δ *vps10*Δ *rox1*Δ	This study
KM_LPVS_	*ura3*Δ *atg1*Δ *lsc1*Δ *pep4*Δ *vps10*Δ *ssn3*Δ	This study
KM_LPVST_	*ura3*Δ *atg1*Δ *lsc1*Δ *pep4*Δ *vps10*Δ *ssn3*Δ *tup1*Δ	This study
KM_LSHW_	*ura3*Δ *atg1*Δ *lsc1*Δ *ssn3*Δ *his3*Δ *trp1*Δ	This study
KM_LSC4D_	*ura3*Δ *atg1*Δ *lsc1*Δ *ssn3*Δ *his3*Δ *trp1*Δ KmYAC [C4+Down]	This study
KM_LSC45D_	*ura3*Δ *atg1*Δ *lsc1*Δ *ssn3*Δ *his3*Δ *trp1*Δ KmYAC [C4+C5+Down]	This study

### Construction of KmYAC carrying designed heme synthesis modules

KmYAC vector (LHZ1015) was composed of *HIS3*, *ARS1*/*CEN5*, *HphMX4*, *TRP1* and KmTEL-filler-KmTEL cassette, in which KmTEL referred to 25 tandem repeats of *K. marxianus* telomeric sequence (Wu et al., 2023). To construct the KmYAC [C4+Down], LHZ1015 was digested with *Not* Ⅰ and *BamH* Ⅰ to release two arms and the filler sequence. After dephosphorylation, the digested product was ligated with *HEM1*_*Km*_ cassette released from LHZ1081, *HEM2*_*Km*_ *-HEM3*_*Sc*_ cassette released from LHZ111, *HEM4*_*Km*_ *-HEM12*_*Sc*_ *-HEM13*_*Km*_ *-HEM14*_*Sc*_ *-HEM15*_*Sc*_ cassette released from LHZ1084, by Seamless Assembly Mix. The ligation product, containing KmYAC [C4+Down], was transformed into KM_ΔHW_ and KM_LSHW_, to obtain KM_C4D_ and KM_LSC4D_, respectively. To construct the KmYAC [C4+C5+Down], the digested and dephosphorylated product of LHZ1015 was ligated with *GLTX*_*Km*_ *-HEMA*_*Sty*_ *-HEML*_*Eco*_ *-HEM1*_*Km*_ cassette released from LHZ1082, *HEM2*_*Km*_ *-HEM3*_*Sc*_ cassette released from LHZ111, *HEM4*_*Km*_ *-HEM12*_*Sc*_ *-HEM13*_*Km*_ *-HEM14*_*Sc*_ *-HEM15*_*Sc*_ cassette released from LHZ1084. The ligation product, containing KmYAC [C4+C5+Down], was transformed into KM_ΔHW_ and KM_LSHW_, to obtain KM_C45D_ and KM_LSC45D_, respectively. Sequences of [C4+Down] and [C4+C5+Down] modules were listed in Supplementary Table S2.

### qPCR

Cells were grown in 50 mL YP medium (40 g/L Glucose, 20 g/L Yeast extract) for 48 h or 72 h. RNA was extracted from cells using a ZR Fungal/Bacterial Miniprep kit (R2014, Zymo Research, United States) and was reverse transcribed using HiScript III All-in-one RT SuperMix Perfect (R333, Vazyme, China). The qPCR was performed using ChamQ Universal SYBR qPCR TB Master Mix (Q711-02, Vazyme, China) in LightCyclery480 (Roche, United States). Primers used in qPCR are listed in Supplementary Table S3.

### Stability of KmYAC and pLBA

KM_LSC4D_-LBA were grown in YD medium overnight and culture was inoculated into fresh 50 mL YD medium to start at an OD_600_ of 0.2. Cells were grown for 24 h (about 7 generations). This process was repeated for 10 consecutive days (approximately 70 generations). The diluted cultures collected after indicated generations were spread on YPD (10 g/L yeast extract, 20 g/L hipolypepton, 20 g/L glucose, 20 g agar) plates, synthetic dropout media without histidine and tryptophan (SC-His-Trp) plates, and synthetic dropout media without histidine, tryptophan and uracil (SC-His-Trp-Ura) plates (Amberg, Burke, and Strathern, 2005). The plates were incubated at 30°C for 1–2 days. The percentage of cells containing KmYAC [C4+Down] was determined by calculating the ratio of colony numbers on SC-His-Trp plates to YPD plates. The percentage of cells containing both KmYAC [C4+Down] and pLBA was determined by calculating the ratio of colony numbers on SC-His-Trp-Ura plates to YPD plates.

### Fed-batch fermentation

Fed-batch fermentations were carried out in a 5 L bioreactor (BXBIO, Shanghai, China). A seed culture was prepared by growing cells in 150 mL SM medium (0.5% (NH_4_)_2_SO_4_, 1% glucose, 0.05% MgSO_4_·7H_2_O, 0.3% KH_2_PO_4_) for 16 h. The seed culture was inoculated into 1.5 L SM medium supplemented with 0.1 g/L glycine, 6% glucose, and 100 µM FeSO_4_·7H_2_O. A total of 1,300 g glucose was continuously added, and the fermentation temperature was maintained at 32°C. The pH was automatically controlled at 5.5 using ammonia, and sterile air was supplied at a rate of 3 L/min for oxygenation. Dissolved oxygen and agitation speed were controlled in a cascade manner within the range of 200–850 rpm.

### Measuring heme content

Cells were grown in 50 mL YD medium for 72 h. The total content of heme and free porphyrin in the cells was measured by a fluorescence-based method as described before (Liu et al., 2014). To accurately measure the heme content in cells grown in a feed-batch fermentor, a modified HPLC method was applied (Zhao, Choi, and Lee, 2018). A total of 1 mL of culture was collected at the indicated time. Cells were pelleted and mixed with 1 mL of acidic acetone (Ko et al., 2021). After centrifugation, the supernatant was mixed with 1 mL of methanol. A hemin chloride standard solution was prepared using acidic acetone and diluted with methanol. The samples were separated on a ZORBAX Eclipse Plus C18 column (250 × 4.6 mm, 5 μm) using a 1,260 Infinity II LC System (Agilent, United States). The column temperature was set at 40°C, and the flow rate was 0.6 mL/min. The mobile phase consisted of 0.05% trifluoroacetic acid (Solution A) and HPLC-grade methanol (Solution B). The gradient program was as follows: 0–1 min, 30% solution B; 1–19 min, a transition from solution A to solution B; 19–30 min, 100% solution B; 30–31 min, a transition from solution B to solution A until 30% solution B was reached; 31–35 min, 30% solution B. The absorbance at 400 nm was measured, and a standard curve was plotted using the peak heights and the concentration of the hemin chloride standard. The heme content in the samples was quantified based on the standard curve.

### Comparison and quantification of LBA titer

Cells were grown in 50 mL YD medium for 72 h. Proteins were extracted by a modified post-alkaline method (Kushnirov, 2000). In brief, 100 μL culture was pelleted and cells were washed by H_2_O. Cells were resuspended in 200 μL NaOH and kept at room temperature for 5 min. Cells were pelleted again and resuspended in 125 μL SDS-PAGE sample buffer (50 mM Tris-HCl (pH 6.8), 2% SDS, 0.1% Bromophenol blue, 10% Glycerol and 1% 2-Mercaptoethanol). Samples were boiled and centrifuged. The supernatant was subjected to SDS-PAGE analysis. LBA-His_6_ was detected by Western blot analysis by using anti-His antibody (AE003, Abclonal, China). Bands in SDS-PAGE were scanned using the Gene Gnome system (Syngene, United Kingdom). The intensity of bands at the position corresponding to LBA in the samples not expressing LBA was considered as the background signal, which was then subtracted from the intensities of bands in the samples expressing LBA. The adjusted intensities of LBA bands were then used to compare the relative yield of LBA in different samples. To quantify the yield of LBA in cells grown in a feed-batch fermentor, cells were collected at the indicated time and proteins were extracted as described above. Samples containing varying concentrations of lactoglobulin (L3908, Sigma-Aldrich, United States) were subjected to SDS-PAGE alongside the LBA samples. Lactoglobulin and LBA are both globular proteins and have a similar size. Lactoglobulin displays a uniform band on SDS-PAGE, making it suitable for the quantitative analysis. The intensities of lactoglobulin bands were quantified, and a standard curve correlating band intensity to protein concentration was established. The LBA concentrations in the culture were then calculated based on this curve.

## Results

### Transferring designed heme synthesis modules by KmYACs to improve heme synthesis and leghemoglobin production

**FIGURE 1:**
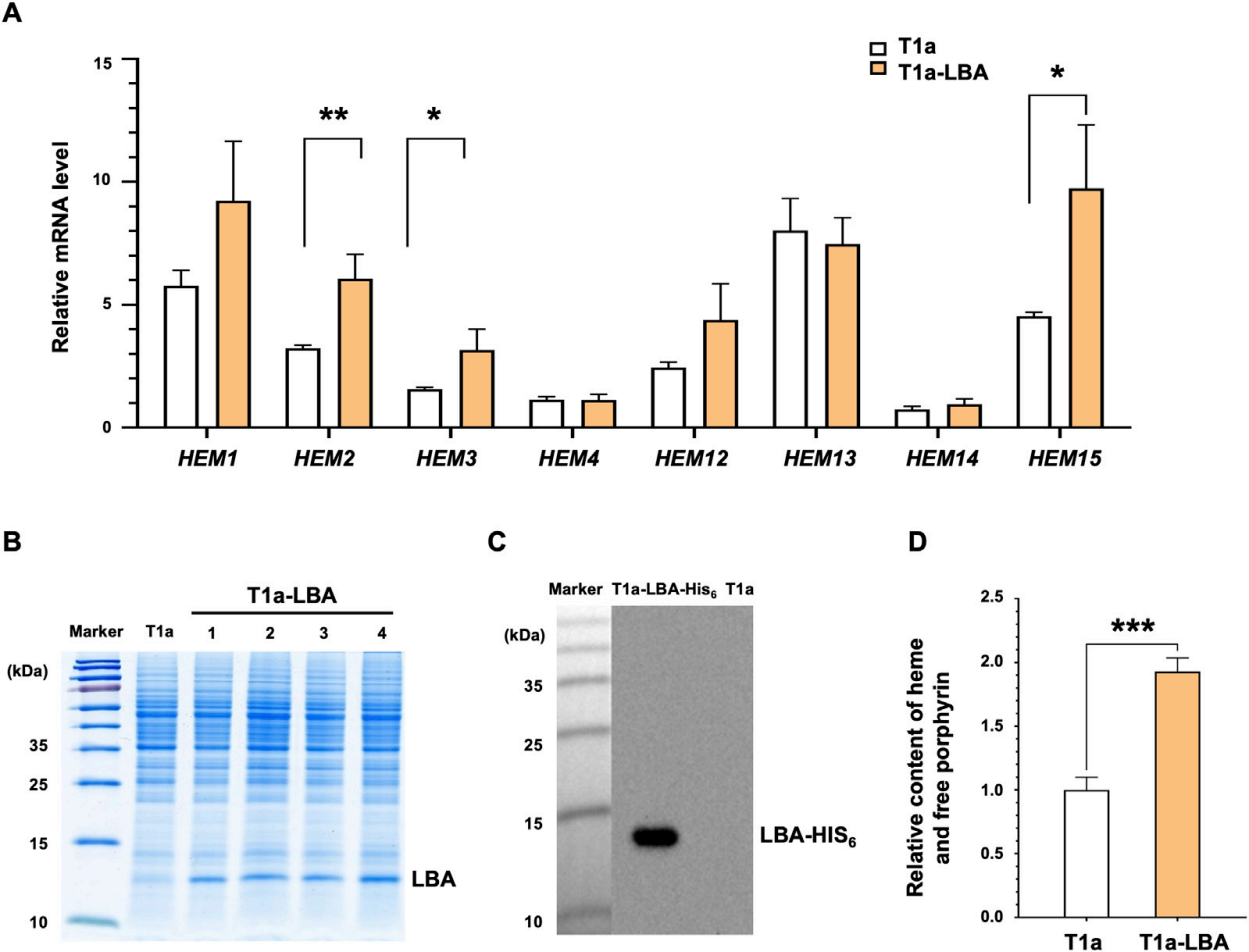
Correlation between heme synthesis and LBA production in *K. marxianus.* **(A)** Relative mRNA levels of the heme synthesis genes pathway in T1a and T1a-LBA. Cells were collected after culturing in YD medium for 48 h. The mRNA level of the heme synthesis gene was calculated relative to that of *SWC4*. Values represent the mean ± S.D. (*n* = 3). **(B)** Expression of LBA in T1a. pLBA was transformed into T1a, resulting in T1a-LBA. Cells were collected after culturing in YD medium for 72 h. The cell lysate was subjected to SDS-PAGE. Four independent clones (1–4) of T1a-LBA were analyzed. **(C)** Western of cell lysate of T1a and T1a-LBA-His_6_. pLBA-His_6_ was transformed into T1a, resulting in T1a-LBA-His_6_. Cells were collected after culturing in YD medium for 72 h. The cell lysate was subjected to SDS-PAGE and Western blot. **(D)** Relative Content of heme and free porphyrin in T1a and T1a-LBA. Cells were cultured in YD for 72 h. The content in T1a was designated as 1. Values represent the mean ± S.D. (*n* = 3). ***, *p* < 0.001.

The bioinformatics analysis revealed the presence of a complete heme synthesis pathway in *K. marxianus*. Similar to *S. cerevisiae*, *K. marxianus* possesses the C4 pathway for the synthesis of 5-ALA and the key gene *GLTX* involved in the first step of the C5 pathway. The expressions of heme synthesis genes were investigated in T1a strain. T1a originated from a wild-type FIM1ΔU strain and displayed an improved yield of heterologous proteins by attenuating autophagy (Liu et al., 2018). After T1a was cultured in YD medium for 48 h, *HEM1*, *HEM13*, *HEM15*, and *HEM2* were highly expressed, compared to a housekeeping gene *SWC4*, while the mRNA levels of *HEM3*, *HEM4*, *HEM12*, and *HEM14* were comparable to that of *SWC4* (Figure 1A). A LBA-expressing plasmid (pLBA) was transformed into T1a to obtain T1a-LBA and LBA was detected in the lysate of T1a-LBA (Figure 1B). To confirm the expression of LBA, a His_6_ tag was fused to the C-terminus of LBA and transformed into T1a to obtain T1a-LBA-His_6_. A clear band was detected by Western blot using anti-His_6_ antibody in the cell lysate of T1a-LBA-His_6_, but not in that of T1a, indicating that the band corresponded to LBA-His_6_ (Figure 1C). The theoretical MW of LBA-His_6_ is 16.2 KDa, but the LBA-His_6_ band detected by Western was still lower than 15 KDa. Previous reports showed that bovine hemoglobin, deer mouse hemoglobin, and cowpea leghemoglobin II exhibited similar faster mobilities in SDS-PAGE (Arredondo-Peter et al., 1997; Natarajan et al., 2011). A recent study observed LBA expressed intracellularly in *C. glutamicum* exhibiting a migration pattern on SDS-PAGE also below 15 kDa (Wang et al., 2023). The results indicate that LBA exhibits a faster mobility in SDS-PAGE, which might be related to the conformation of LBA. Compared with T1a, the mRNA levels of *HEM*2, *HEM3* and *HEM15* increased by 87%, 103%, and 115%, respectively in T1a-LBA (Figure 1A). Therefore, expression of heterologous LBA in T1a significantly increased the transcription levels of certain heme synthesis genes. After culturing for 72 h, the total content of heme and free porphyrin in T1a-LBA was 92.8% higher than the content in T1a (Figure 1D). This increase might be related to the elevated expression of *HEM2*, *HEM3*, and *HEM15* in T1a. Leghemoglobin needs to combine with heme to form the stable form of leghemoglobin-heme. The consumption of heme might alleviate the feedback inhibition on the enzyme(s) within the heme biosynthesis pathway, thereby promoting heme synthesis. Therefore, the synthesis of hemoglobin and heme can mutually promote each other (Ishchuk et al., 2021; Shao et al., 2022).

**FIGURE 2:**
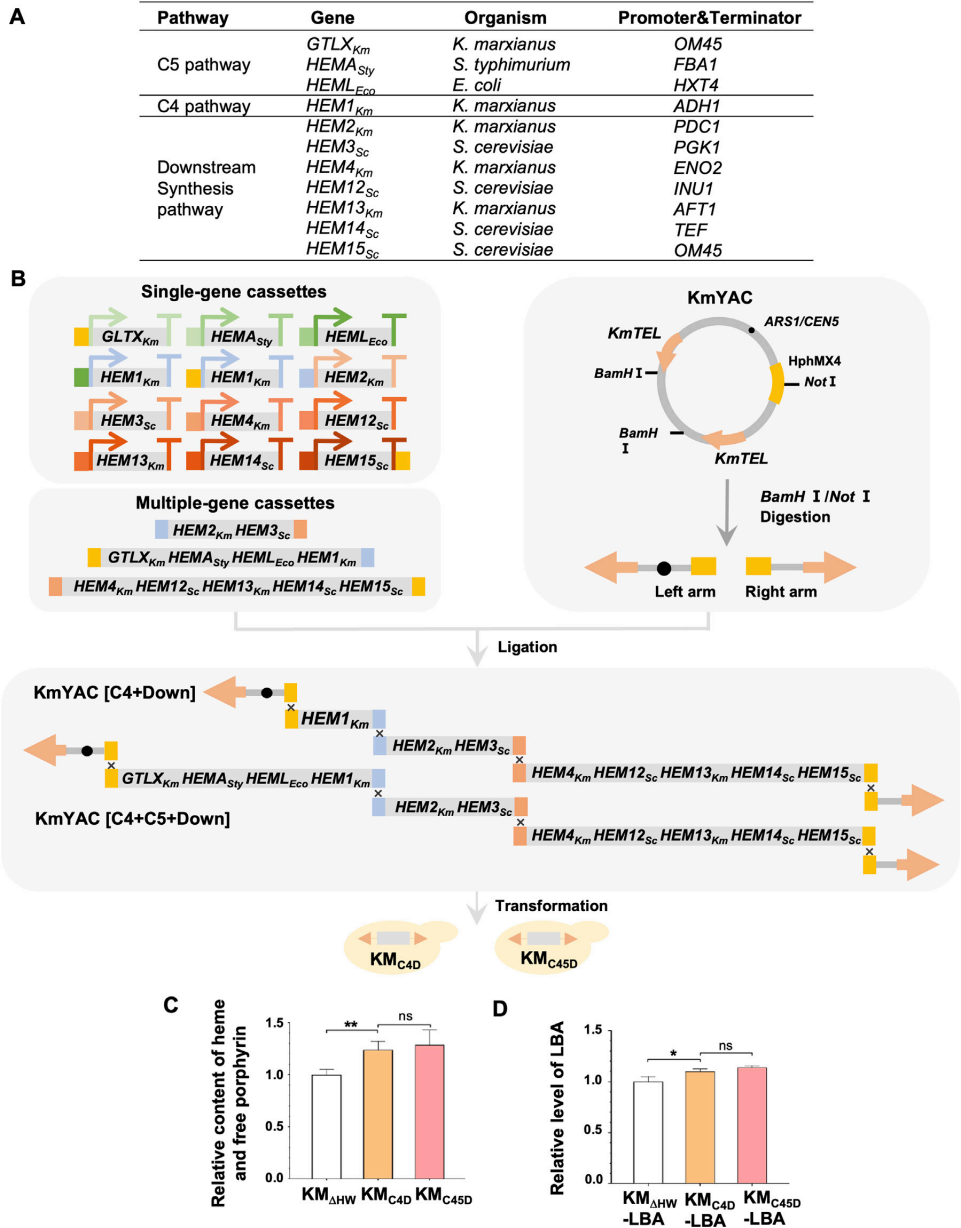
Remodeling of *K. marxianus* heme synthesis pathway by YAC. **(A)** Genes included in the heme synthesis module. **(B)** Flow chart of constructing KM-YAC that carried designed heme synthesis modules. KmYAC [C4+Down] and KmYAC [C4+C5+Down] were transformed into KM_DHW_ to obtain KM_C4D_ and KM_C45D_, respectively. **(C)** Relative content of heme and free porphyrin in KM_ΔHW_, KM_C4D_, and KM_C45D_. Cells were cultured in YD medium for 72 h. The content in KM_ΔHW_ was designated as 1. Values represent the mean ± S.D. (*n* = 3). **, *p* < 0.01. ns, not significant. **(D)** Relative levels of LBA in KM_ΔHW_-LBA, KM_C4D_-LBA and KM_C45D_-LBA. pLBA was transformed into KM_ΔHW_, KM_C4D_, and KM_C45D_, resulting in KM_ΔHW_-LBA, KM_C4D_-LBA and KM_C45D_-LBA, respectively. The transformants were then cultured in YD medium for 72 h. The level of LBA in KM_ΔHW_-LBA was designated as 1. Values represent the mean ± S.D. (*n* = 3). *, *p* < 0.05.

Therefore, we aimed to increase the production of LBA in *K. marxianus* by enhancing heme synthesis. To design a novel heme synthesis module, we selected heme synthesis genes from different species, including three genes from the C5 pathway, the native *K. marxianus HEM1* from the C4 pathway, and seven genes from the heme downstream synthesis pathway (Figure 2A). Each gene’s expression was driven by a strong promoter and terminator of *K. marxianus* in a single-gene cassette (Figure 2A) (Wu et al., 2022). Single-gene cassettes were ligated to construct multiple-gene cassettes. Different single-gene and multiple-gene cassettes were then combined to build two designed heme synthesis modules, [C4+Down] and [C4+C5+Down]. [C4+down] module included native *HEM1*_*Km*_ from the C4 pathway, three native genes (*HEM2*_*Km*_, *HEM4*_*Km*_, *HEM13*_*Km*_) and four *S. cerevisiae* genes (*HEM3*_*Sc*_, *HEM12*_*Sc*_, *HEM14*_*Sc*_, *HEM15*_*Sc*_) from downstream pathways, while the [C4+C5+down] module contained an additional native *GTLX*_*Km*_ and two bacterial genes (*HEMA*_*Sty*_, *HEML*_*Eco*_) from C5 pathways. Two modules were ligated with a yeast artificial chromosome (KmYAC), respectively. The resultant KmYAC [C4+Down] and KmYAC [C4+C5+Down] were transformed into KM_ΔHW_ to obtain KM_C4D_ and KM_C45D_, respectively (Figure 2B). After 72 h of culturing, the total heme/free porphyrin content in KM_C4D_ and KM_C45D_ increased by 23% and 28%, respectively, compared to that in KM_ΔHW_ (Figure 2C). pLBA was transformed into KM_ΔHW_ to obtain KM_ΔHW_-LBA, KM_C4D_-LBA and KM_C45D_-LBA, respectively. Compared with KM_ΔHW_-LBA, the levels of LBA in KM_C4D_-LBA and KM_C45D_-LBA increased by 10.1% and 14.0%, respectively, (Figure 2D). These results demonstrated that the designed heme synthesis modules, transferred by KmYACs, significantly improved heme synthesis and the yield of LBA in *K. marxianus*.

### Improving heme synthesis and LBA production by deleting inhibitory genes

**FIGURE 3:**
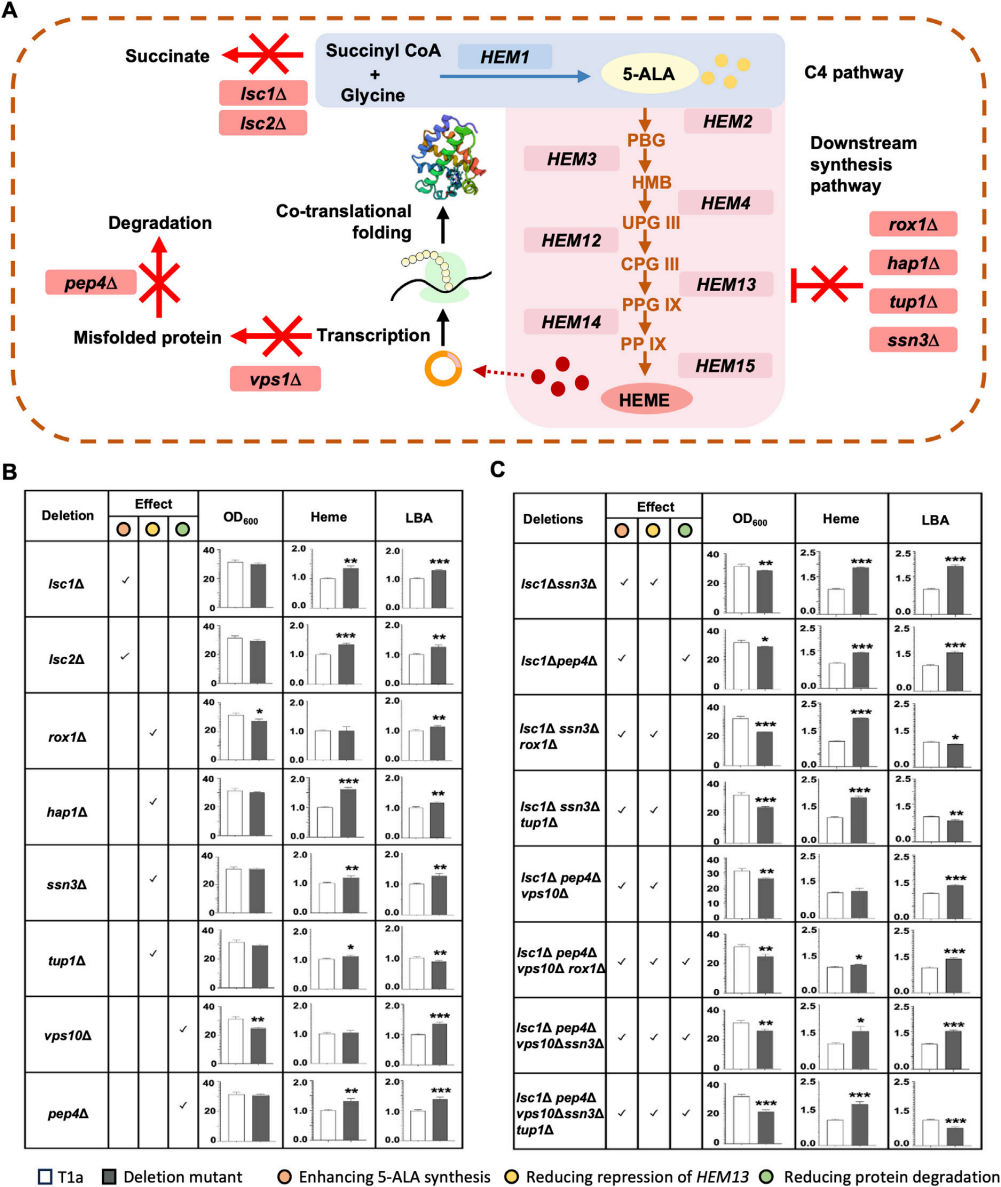
Improving heme synthesis and LBA production by deleting inhibitory genes. **(A)** Schematic diagram of effects of deletions. Deletions were expected to abolish the synthesis of succinate (*lsc1*Δ, *lsc2*Δ), repression of *HEM13* (*rox1*Δ, *hap1*Δ, *tup1*Δ, *ssn3*Δ), or degradation of misfolded proteins (*vps10*Δ, *pep4*Δ). **(B,C)** Effect of deleting a single gene **(B)** or multiple genes **(C)** on the biomass, heme and free porphyrin content, and LBA production. Predicted effects of deletion(s) on enhancing 5-ALA synthesis, reducing repression of *HEM13*, and reducing protein degradation were checked. Genes were deleted in T1a. OD_600_ and the total content of heme and free porphyrin in T1a and deletion mutant were compared after culturing in YD medium for 72 h. To compare the production of LBA, pLBA was transformed into T1a and mutants, respectively. The resulting transformants were cultured in YD medium for 72 h. Heme content and level of LBA in T1a were designated as 1 when compared to mutants. Values represent the mean ± S.D. (*n* = 3). *, *p* < 0.05, **, *p* < 0.01, ***, *p* < 0.001.

To improve cellular heme content and the yield of LBA, several genes exhibiting inhibitory effects on heme synthesis or LBA production were selected for deletion (Figure 3A). In *C. glutamicum, sucC* and *sucD* encode two subunits of succinyl-CoA ligase, responsible for converting succinyl-CoA into succinate. Deletion of *sucC* and *sucD* abolishes the flux to succinate and instead enhances the synthesis of 5-ALA from succinyl-CoA and glycine (Yang et al., 2016). Deletion of *ROX1*, *HAP1*, *SSN6*, or *TUP1* reduces the inhibition of *HEM13*, which encodes a key enzyme in the downstream synthesis pathway of heme (Keng, 1992; Kastaniotis and Zitomer, 2000; Martínez et al., 2015). The deletion of *VPS10* and *PEP4* reduces the degradation of hemoglobin in the vacuole (Ishchuk et al., 2021). Based on sequence homology analysis, 8 genes were identified as homologues of the above-mentioned genes in *K. marxianus*. They were individually deleted in T1a, resulting in eight single-gene deletion mutants. The biomass, the total content of heme and free porphyrin, and LBA production of T1a and deletion mutants were measured and compared side by side (Figure 3B). Deletion of *ROX1* or *VPS10* significantly reduced biomass, while other deletions did not affect biomass. Deletion of *LSC1*, *LSC2*, *HAP1*, *SSN*3, *TUP1* or *PEP4* significantly improves the total content of heme and free porphyrin.

Among the eight mutants, the *lsc1*Δ mutant demonstrated the greatest improvement in heme and leghemoglobin production. Specifically, compared with T1a, the *lsc1*Δ mutant exhibited a 35% increase in the total content of heme and free porphyrin, as well as a 28% increase in LBA production. Therefore, eight multiple-gene deletion mutants were constructed in the *lsc1*Δ background (Figure 3C). All eight multiple-gene deletion mutants showed decreased biomass after 72 h of culturing, indicating a burden on cell growth due to multiple deletions. Meanwhile, four mutants showed simultaneously increased heme content and LBA production. Among them, the *lsc1*Δ*ssn3*Δ mutant performed the best, displaying an 87% increase in the total content of heme and free porphyrin, as well as a 97% increase in LBA production. This mutant was designated as KM_LS_ and selected for further engineering.

### Remodeling the heme synthesis pathway by combining designed heme synthesis modules and deletions of inhibitory genes

Our results indicate that the designed heme synthesis modules transferred by KmYACs, as well as the deletions of *LSC1* and *SSN3*, significantly improve heme synthesis and the yield of LBA in *K. marxianus*. Therefore, we aimed to combine these two strategies to achieve further improvement. *HIS3* and *TRP1* were deleted in KM_LS_ to create auxotrophic markers to accept KmYAC and the resultant strain named KM_LSHW_. KmYAC [C4+Down] and KmYAC [C4+C5+Down] were transformed into KM_LSHW_ to obtain KM_LSC4D_ and KM_LSC45D_, respectively.

**FIGURE 4:**
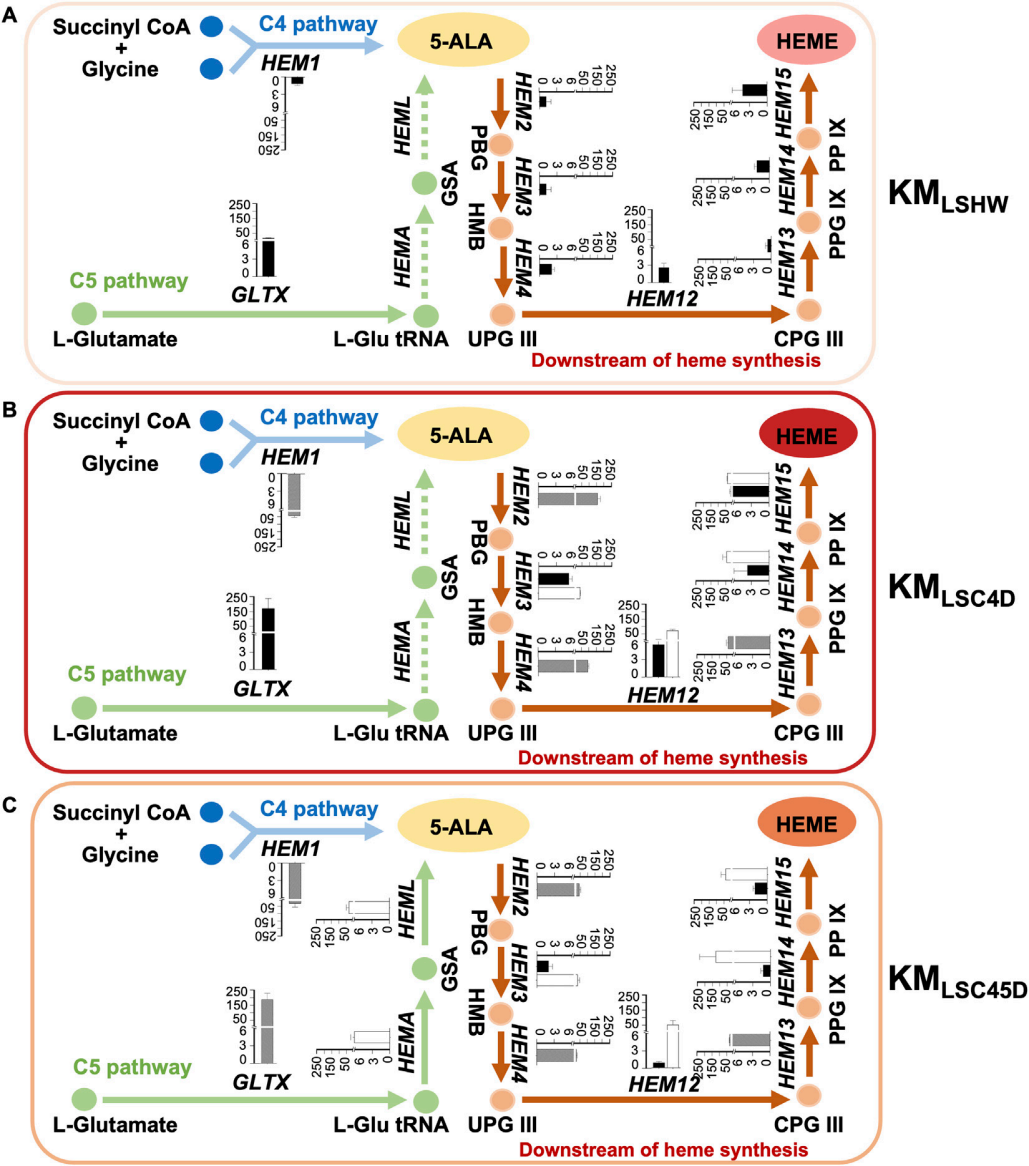
Expressions of heme synthesis genes in strains containing designed heme synthesis modules and deletions of inhibitory genes. **(A–C)** The relative mRNA levels of the heme synthesis genes pathway in KM_LSHW_ **(A)**, KM_LSC4D_ **(B)**, and KMLS_C45D_ **(C)**. KmYAC [C4+Down] and KmYAC [C4+C5+Down] were transformed into KM_LSHW_ to obtain KM_LSC4D_ and KM_LSC45D_, respectively. Cells were collected after culturing in YD medium for 72 h. The mRNA levels of heme synthesis genes were calculated relative to that of *SWC4*. The relative mRNA levels of genomic *K. marxianus* genes were represented by black columns, while those of the heterologous genes introduced by KmYAC were represented by white columns. The relative mRNA levels of *K. marxianus* genes expressed from one genomic copy and one additional copy introduced by KmYAC were represented by grey columns. The values represent the mean ± S.D. (*n* = 3).

The expressions of heme synthesis genes in KM_LSHW_, KM_LSC4D_ and KM_LSC45D_ were investigated (Figure 4). In KM_LSC4D_, the expression of *HEM1*_*Km*_, *HEM2*_*Km*_, *HEM4*_*Km*_, or *HEM13*_*Km*_ was derived from one native gene in the genome and an additional copy transferred by KmYAC (Figure 4B, gray column). The relative mRNA levels of *HEM1*_*KM*_, *HEM2*_*Km*_, *HEM4*_*Km*_, and *HEM13*_*Km*_ in KM_LSC4D_ were 32, 136, 43, and 82 times higher than the corresponding genes in KM_LSHW_, where the expression of *HEM1*_*Km*_, *HEM2*_*Km*_, *HEM4*_*Km*_, and *HEM13*_*Km*_ only originated from the native genes (Figure 4A, black column). The results indicate transferring *K. marxianus* heme synthesis gene by KmYAC significantly upregulates the overall expression of these genes. Four heme synthesis genes from *S. cerevisiae*, including *HEM3*_*Sc*_, *HEM12*_*Sc*_, *HEM14*_*Sc*_, *and HEM15*_*Sc*_ were all actively expressed in KM_LSC4D_ (Figure 4B, white columns). Notably, the presence of these heterologous genes significantly enhanced the expression of their orthologues (Figure 4B, black columns), suggesting a mechanism to promote the co-upregulation of orthologous genes.

In KM_LSC45D_, the expression of *GLTX*_*Km*_, *HEM*_*Km*_, *HEM2*_*Km*_, *HEM4*_*Km*_, or *HEM13*_*Km*_ was a combined expression from a native genomic copy and a KmYAC-transferred copy (Figure 4C, gray columns). Similar to the situation in KM_LSC4D_, the relative mRNA levels of *GLTX*_*Km*_, *HEM1*_*Km*_, *HEM2*_*Km*_, *HEM4*_*Km*_, and *HEM13*_*Km*_ in KM_LSC45D_ were 12, 21, 25, 6, and 26 times higher respectively than the corresponding genes in KM_LSHW_ (Figure 4A, black column). In KM_LSC4D_, the heterologous *HEMA*_*Sty*_, *HEML*_*Eco*_, *HEM3sc*, *HEM12*_*Sc*_, *HEM14*_*Sc*_, and *HEM15*_*Sc*_ introduced by KmYAC were all actively expressed (Figure 4C, white columns). However, the expressions of endogenous *HEM3*_*Km*_, *HEM14*_*Km*_, and *HEM15*_*Km*_ were not significantly different from those in KM_LSHW_, while the expression of *HEM12*_*Km*_ in KM_LSC45D_ was significantly reduced compared to that in KM_LSHW_ (Figures 4A, C, black columns). The result suggests that the transfer of heterologous genes in C5 pathways might interfere with the co-upregulation of orthologous genes in the downstream pathway.

There was no difference in the expressions of *HEM3sc*, *HEM12*_*Sc*_, *HEM14*_*Sc*_, and *HEM15*_*Sc*_ in KM_LSC4D_ and KM_LSC45D_ (Figures 4B, C, white columns), indicating the expressions of *S. cerevisiae* genes were stable in different environments. However, the expressions of *K. marxianus* genes in the downstream pathway were significantly higher in KM_LSC4D_ compared to the KM_LSC45D_ strain (Figures 4B, C, gray and black columns).

**FIGURE 5:**
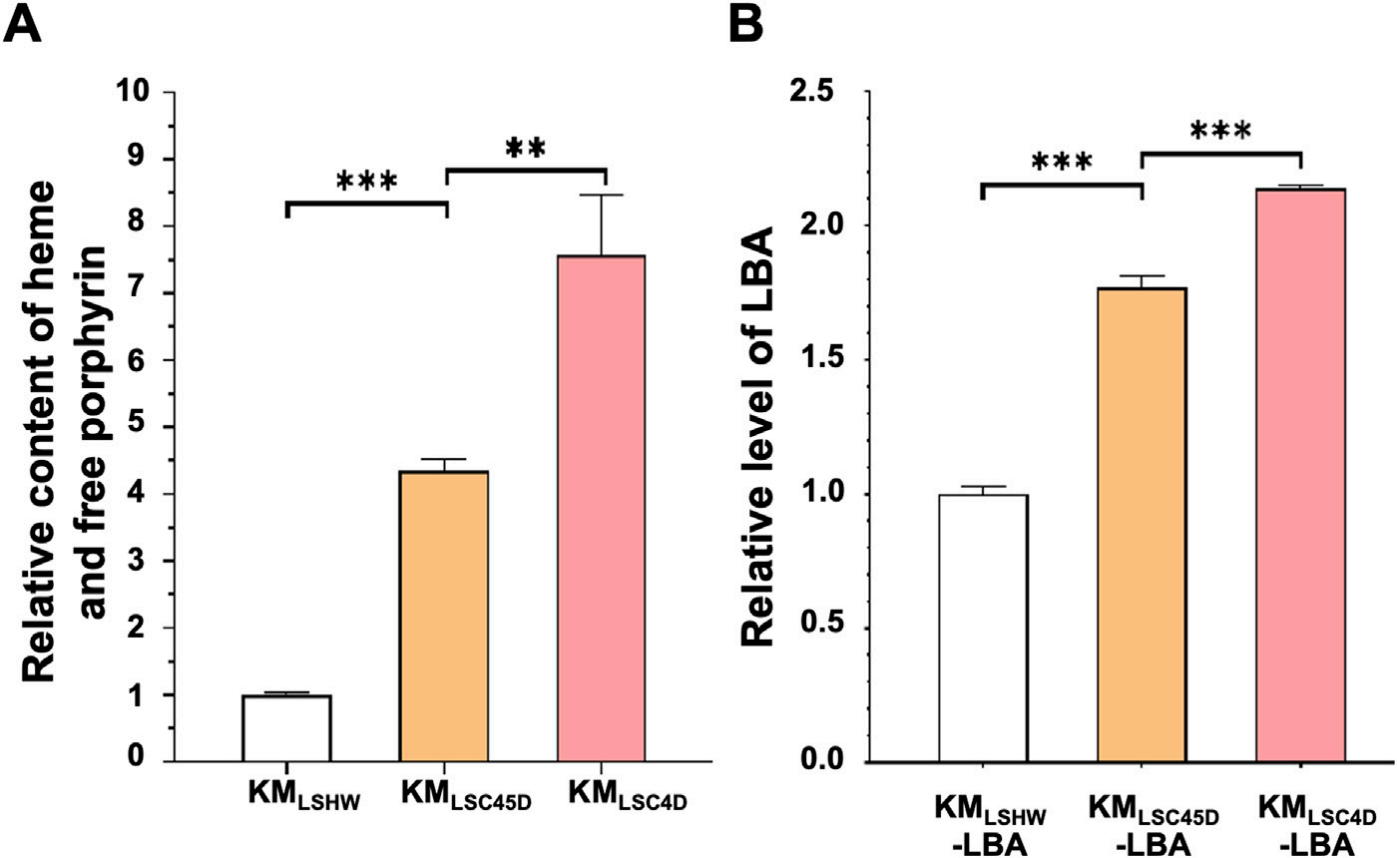
The heme content and LBA production in strains containing designed heme synthesis modules and deletion of inhibitory genes. **(A)** The relative contents of heme and free porphyrin in KM_LSC4D_ and KM_LSC45D_. Cells were cultured in YD medium for 72 h. The heme content in KM_LSHW_ was designated as 1. The values represent the mean ± S.D. (*n* = 3). **, *p* < 0.01, ***, *p* < 0.001. **(B)** The relative levels of LBA produced in KM_LSC4D_ and KM_LSC45D_. pLBA was transformed into KM_LSHW_, KM_LSC4D_ and KM_LSC45D_, resulting in KM_LSHW_-LBA, KM_LSC4D_-LBA and KM_LSC45D_-LBA, respectively. The transformants were then cultured in YD medium for 72 h. The level of LBA produced in KM_LSHW_-LBA was designated as 1.

As shown in Figure 5, the total contents of heme and free porphyrin in KM_LSC4D_ and KM_LSC45D_ were 7.57 times and 5.34 times that of KM_LSHW_, respectively (Figure 5A). pLBA were transformed into KM_LSHW_, KM_LSC4D_ and KM_LSC45D_, resulting in KM_LSHW_-LBA, KM_LSC4D_-LBA and KM_LSC45D_-LBA, respectively. The levels of LBA produced in KM_LSC4D_-LBA and KM_LSC45D_-LBA increased by 113% and 76.9% respectively, compared to that in KM_LSHW_-LBA (Figure 5B). Compared to KM_LSC45D_, higher heme accumulation and LBA production in KM_LSC4D_ might be attributed to the relatively high expressions of *K. marxianus* genes in the downstream pathway.

### Optimization of the medium recipe and feed-batch fermentation for improved yield of LBA

**FIGURE 6:**
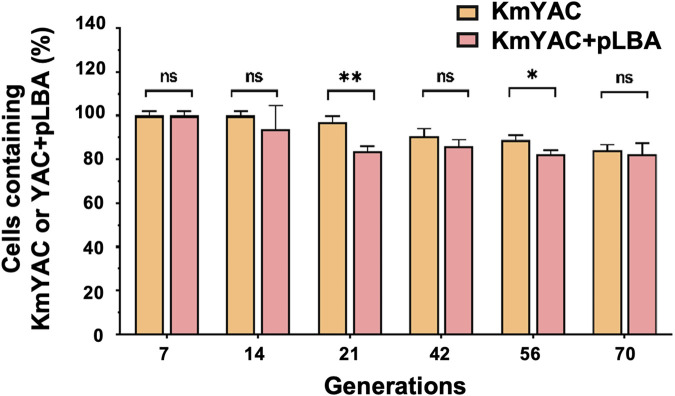
Stability of KmYAC and LBA-expressing plasmid in KM_LSC4D_. KM_LSC4D_-LBA were grown for 70 generations without selective pressure. The percentage of cells containing KmYAC [C4+Down] and pLBA was calculated after growing for 7, 14, 21, 42, 56, and 70 generations. The values represent the mean ± S.D. (*n* = 3). *, *p* < 0.05. **, *p* < 0.01. ns, not significant.

The improved concentration of cellular heme and the yield of LBA were dependent on the presence of KmYAC [C4+Down] and pLBA. Therefore, the stabilities of KmYAC [C4+Down] and pLBA in KM_LSC4D_-LBA became a concern during industrial production and were thus investigated. KM_LSC4D_-LBA was cultured in YD medium without selective pressure for 70 generations. After 70 generations, 84.09% of cells contained KmYAC [C4+Down], and 82.46% of cells contained both KmYAC [C4+Down] and pLBA (Figure 6). The result indicate the robust and stable propagation of both KmYAC [C4+Down] and pLBA during mitosis, which will facilitate their applications in industrial production.

**FIGURE 7:**
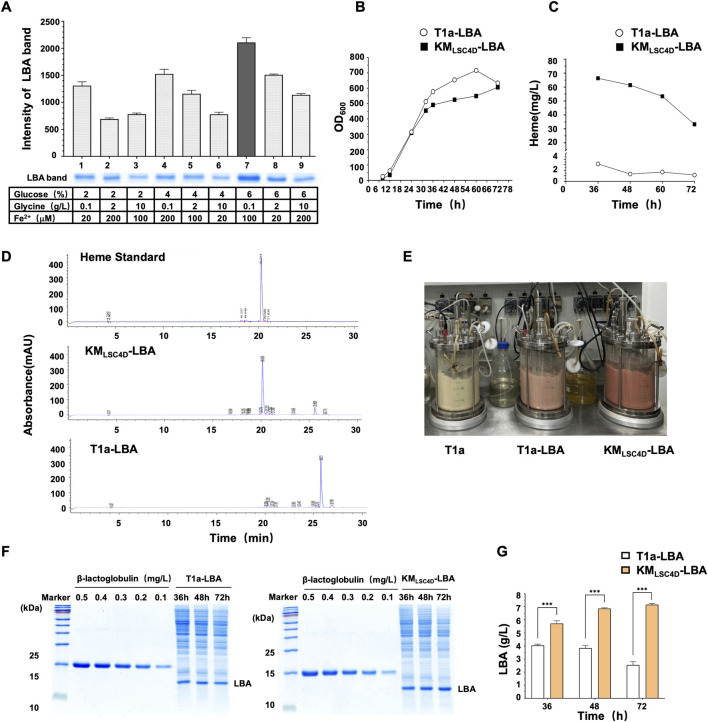
Optimization of the medium recipe and feed-batch fermentation for improved yield of LBA. **(A)** Optimization of the medium recipe to improve the yield of LBA. KM_LSC4D_-LBA was grown in a medium with the indicated concentrations of glucose, glycine, and FeSO_4_·7H_2_O (Fe^2+^) for 72 h. The cell lysate was subjected to SDS-PAGE. The bands of LBA were scanned in grayscale, and the intensities of the bands were quantified. The values represent the mean ± S.D. (*n* = 3). **(B)** Growth curves of KM_LSC4D_-LBA and T1a-LBA in a 5L fermentor. pLBA was transformed into T1a, resulting in T1a-LBA, which served as a control. Cells were grown in the optimized medium in a feed-batch 5L fermentor for 72 h. **(C,D)** Comparison of heme concentrations in KM_LSC4D_-LBA and T1a-LBA during feed-batch fermentation. Cells were collected at the indicated times during feed-batch fermentation described in **(B)**. The concentrations of heme in the cell lysate at each time point were quantified by HPLC and compared **(C)**. A chromatogram map of 36-h samples was shown in **(D)**. **(E)** Image of cells in the fermentor after 36 h. **(F)** SDS-PAGE of cell lysate of KM_LSC4D_-LBA and T1a-LBA during feed-batch fermentation. Cells were collected at the indicated times during feed-batch fermentation described in **(B)**. The cell lysate was diluted and subjected to SDS-PAGE. The amount of LBA was semi-quantified using a β-lactoglobulin standard. **(G)** Comparison of LBA titer in KM_LSC4D_-LBA and T1a-LBA during feed-batch fermentation. The values represent the mean ± S.D. (*n* = 3). ***, *p* < 0.001.

To further enhance LBA yield, we conducted an orthogonal experiment to optimize the recipe of synthetic medium (SM). We varied three concentrations each of glucose (2%, 4%, and 6%), glycine (0.1 g/L, 10 g/L, and 20 g/L), and FeSO_4_·7H_2_O (20 μM, 100 μM, 200 μM), resulting in nine distinct media. KM_LSC4D_-LBA was cultured in these media, and titers of LBA were quantified by the grayscale intensities of the bands in SDS-PAGE (Figure 7A). The analysis of variance revealed that the concentrations of glucose, glycine, and FeSO_4_·7H_2_O significantly influenced LBA production. According to the range analysis, the impact of these factors on LBA titers was determined as follows: glycine concentration > glucose concentration > iron ion concentration. The recipe that achieved the highest yield of LBA was the SM supplemented with 0.1 g/L of glycine, 6% glucose, and 100 µM FeSO_4_·7H_2_O.

The optimized recipe was applied in the SM medium for the feed-batch fermentation of KM_LSC4D_-LBA. As a control, LBA was transformed into T1a, resulting in T1a-LBA. The fermentation of both strains was carried out in a 5L fermentor at 32°C for 72 h. The growth rate of KM_LSC4D_-LBA was slightly lower than that of T1a-LBA during the exponential stage, but the biomass of both strains reached the same level after 72 h (Figure 7B). The heme content in the cell lysate of KM_LSC4D_-LBA and T1a-LBA were analyzed by quantitative HPLC and compared. The highest heme contents were observed after 36 h, with the content in KM_LSC4D_-LBA reaching 66.32 mg/L, which was 23.74 times higher than that in T1a-LBA (Figures 7C, D). The higher heme content in KM_LSC4D_-LBA resulted in a noticeably redder culture compared to that of T1a-LBA (Figure 7E). In KM_LSC4D_-LBA, accumulated heme might exceed its consumption by LBA and other cellular processes. Excess heme could potentially induce toxicity within the cells (Anzaldi and Skaar, 2010), leading to cell lysis or heme degradation, subsequently causing a noticeable decrease in heme titers after 36 h (Figure 7C). The titer of LBA in KM_LSC4D_-LBA was quantified by a lactoglobulin standard (Figure 7F). Throughout the fermentation, the titer of LBA in KM_LSC4D_-LBA consistently surpassed that in T1a-LBA at every time point (Figure 7G). Additionally, the titer of LBA in KM_LSC4D_-LBA exhibited gradual increments during fermentation. In contrast, the yield of LBA in T1a-LBA declined, possibly due to the instability of LBA resulting from insufficient heme available for binding. After 72 h, the titer of LBA in KM_LSC4D_-LBA reached 7.27 g/L, which was 80% higher than the titer in T1a-LBA (4.04 g/L) and represented the highest titer of intracellular leghemoglobin reported to date (Figure 7G).

## Discussion

Microbial cell factories offer a sustainable, high-yield, and cost-effective source of leghemoglobin to enhance the sensory qualities of plant-based meat. Secretory expression of leghemoglobin simplifies purification procedures and is favored in industrial production. Currently, the highest secretory expression of LBC2 has been achieved in *P. pastoris*, with a titer of 3.5 g/L, making *P. pastoris* a favorable host for leghemoglobin production. However, while some proteins expressed by *P. pastoris* are considered food-safe, *P. pastoris* itself has not yet obtained Generally Recognized as Safe (GRAS) status (Barone et al., 2023). During the construction of *P. pastoris* strains for leghemoglobin production, the high-copy integration of the target gene into the genome is achieved through the selection of the G418 antibiotic marker. Within a certain range, the yield of the recombinant protein is positively correlated with the degree of G418 resistance (Lin-Cereghino et al., 2008). Embedding antibiotic resistance of *P. pastoris* strains might bring potential risks (Baghban et al., 2019). Additionally, *P. pastoris* is a methylotrophic yeast, and methanol induction is required to induce the expression of recombinant proteins, raising safety concerns related to the final product. Studies have reported no allergenicity or toxicity associated with the recombinant LBC2 expressed in *P. pastoris*, as well as with the 17 *P. pastoris* host proteins co-purified with LBC2 (Jin et al., 2018). Nevertheless, the presence of host proteins from a species lacking a GRAS certificate still raises certain safety concerns.

Four types of leghemoglobin are induced at different stages in root nodules. The predominant type of leghemoglobin expressed by *P. pastoris* is LBC2. In this study, we chose to work with LBA because it lacks predicted glycosylation sites (N-X-S/T) and has demonstrated a relatively higher yield in *K. marxianus*. Considering that *K. marxianus* has attained GRAS status (Leonel et al., 2021), there are fewer safety concerns associated with the LBA product produced by *K. marxianus*. We investigated both intracellular and secretory expressions of LBA in *K. marxianus*. While achieving a high yield of intracellular expression of LBA in this study, we observed a low yield in secretory expression. Currently, we are optimizing the secretory expression method.

*K. marxianus* is a crabtree-negative strain known for its rapid growth and high glucose consumption under aerobic conditions. The results of the orthogonal experiment indicate glucose is a pivotal factor influencing LBA production. Consequently, during the feed-batch fermentation, the glucose feeding rate was controlled based on dissolved oxygen levels, typically maintained within the range of 5%–10%. This approach ensured both rapid cell growth and efficient LBA production. The highest LBA production was achieved in 72 h of fermentation, ensuring optimal efficiency for industrial-scale production.

The heme synthesis pathway of KM_LSC4D_ was remodeled by deleting *LSC1* and *SSN3*, which inhibited heme synthesis, and by introducing a designed C4+down module through KmYAC. The expression of native heme synthesis genes was significantly upregulated in KM_LSC4D_ (Figure 4B, black columns). The introduction of additional heme synthesis modules might accelerate the consumption of intermediate metabolites of heme and promote the cycling of the heme metabolism. As a result, the total heme and porphyrin content in KM_LSC4D_ was 6.57 times higher compared to the control. In comparison with KM_LSC4D_, KM_LSC45D_ contained additional heme synthesis genes from the C5 pathway. However, the enhancement in total heme and porphyrin content in KM_LSC45D_ was reduced to 5.34 times that of the control. The expressions of *S. cerevisiae* heme synthesis genes did not differ significantly between KM_LSC4D_ and KM_LSC45D_, while the expressions of *K. marxianus* heme synthesis genes in KM_LSC4D_ were significantly higher than those in KM_LSC45D_. These results suggest that heterologous modules transferred by KmYAC, represented by *S. cerevisiae* heme synthesis genes, exhibit a certain degree of orthogonality and are more robust in countering disturbances caused by the introduction of additional genes. Therefore, the introduction of heterologous modules, rather than native *K. marxianus* modules, tends to result in the expected and orthogonal expressions in *K. marxianus*, thereby facilitating the engineering of strains for improved yield.

In summary, in food-grade *K. marxianus*, we intentionally deleted genes that inhibit heme synthesis and strategically combined them with heme synthesis modules carried by KmYACs. Compared to the wild-type strain, the engineered strains achieved enhanced heme content, increasing by 23.74 times to reach 66.32 mg/L. LBA produced in the engineered strain reached 7.2 g/L, marking the highest intracellular expression of recombinant leghemoglobin to date. The utilization of recombinant LBA expressed in *K. marxianus* as a substitute ingredient for animal meat will enhance food safety and reduce reliance on animal resources, making a significant contribution to sustainability and environmental protection.

## Data Availability

The original contributions presented in the study are included in the article/Supplementary Material, further inquiries can be directed to the corresponding authors.
