# Supplementary material for: High-level expression of leghemoglobin in Kluyveromyces marxianus by remodeling the heme metabolism pathway
Source: Front Bioeng Biotechnol. 2024 Jan 8;11:1329016. doi: 10.3389/fbioe.2023.1329016 (PMC10804453; doi:10.3389/fbioe.2023.1329016)
Supplement: Supplementary file 1 [file Table1.DOCX]

**Supplementary Material**

Table S1. Plasmids used in this study.

| **Name** | **Essential features** | **application** | **Backbone** | **Source** |
| --- | --- | --- | --- | --- |
| pLBA  (LHZ1085) | pKD1, KmURA3, *LBA* | Intracellular expression of LBA | pUKDN115  (Duan et al. 2019) | This study |
| pLBA-His_6_  (LHZ1463) | pKD1, KmURA3, *LBA*-*His_6_* | Intracellular expression of LBA-His_6_ | pLBA | This study |
| LHZ1081 | P_ADH1_- *HEM1_Km_* -T_ADH1_ | Providing cassette of *HEM1_Km_* | pMD18-T | This study |
| LHZ1110 | P_PDC1_- *HEM2_Km_*-T_PDC1_ | Providing cassette of *HEM2_Km_* | pMD18-T | This study |
| LHZ1111 | P_PGK1_- *HEM3_Sc_* -T_PGK1_ | Providing cassette of *HEM3_Sc_* | pMD18-T | This study |
| LHZ1112 | P_ENO2_- *HEM4_Km_* -T_ENO2_ | Providing cassette of *HEM4_Km_* | pMD18-T | This study |
| LHZ1113 | P_INU1_- *HEM12_Sc_* -T_INU1_ | Providing cassette of *HEM12_Sc_* | pMD18-T | This study |
| LHZ1114 | P_AFT1_- *HEM13_Km_* -T_AFT1_ | Providing cassette of *HEM13_Km_* | pMD18-T | This study |
| LHZ1115 | P_TEF_- *HEM14_Sc_* -T_TEF_ | Providing cassette of *HEM14_Sc_* | pMD18-T | This study |
| LHZ1116 | P_OM45_- *HEM15_Sc_* -T_OM45_ | Providing cassette of *HEM15_Sc_* | pMD18-T | This study |
| LHZ1117 | P_FBA1_- *HEMA_Sty_* -T_FBA1_ | Providing cassette of *HEMA_Sty_* | pMD18-T | This study |
| LHZ1118 | P_HXT4_- *HEML_Eco_* -T_HXT4_ | Providing cassette of *HEML_Eco_* | pMD18-T | This study |
| LHZ1119 | P_OM45_- *GLTX_Km_* -T_OM45_ | Providing cassette of *GLTX_Km_* | pMD18-T | This study |
| LHZ1082 | P_FBA1_- *HEMA_Sty_* -T_FBA1_,  P_HXT4_- *HEML_Eco_* -T_HXT4,_ P_OM45_- *GLTX_Km_* -T_OM45_, P_ADH1_- *HEM1_Km_* -T_ADH1_ | Providing cassette of *GLTX_Km_-HEMA_Sty_-HEML_Eco_-HEM1_Km_* | pMD18-T | This study |
| LHZ1083 | P_PDC1_- *HEM2_Km_*-T_PDC1_, P_PGK1_- *HEM3_Sc_* -T_PGK1_ | Providing cassette of *HEM2_Km_ -HEM3_Sc_* | pMD18-T | This study |
| LHZ1084 | P_ENO2_- *HEM4_Km_* -T_ENO2_, P_INU1_- *HEM12_Sc_* -T_INU1_, P_AFT1_- *HEM13_Km_* -T_AFT1_, P_TEF_- *HEM14_Sc_* -T_TEF_, P_OM45_- *HEM15_Sc_* -T_OM45_ | Providing cassette of *HEM4_Km_-HEM12_Sc_-HEM13_Km_-HEM14_Sc_-HEM15_Sc_* | pMD18-T | This study |
| LHZ1128 | pKD1, KmURA3, Cas9, gRNA- *LSC1* | CRISPR plasmid to delete *LSC1* | LHZ531  (Shi et al. 2021) | This study |
| LHZ1129 | pKD1, KmURA3, Cas9, gRNA- *LSC2* | CRISPR plasmid to delete *LSC2* | LHZ531 | This study |
| LHZ1130 | pKD1, KmURA3, Cas9, gRNA- *SSN3* | CRISPR plasmid to delete *SSN3* | LHZ531 | This study |
| LHZ1131 | pKD1, KmURA3, Cas9, gRNA- *ROX1* | CRISPR plasmid to delete *ROX1* | LHZ531 | This study |
| LHZ1132 | pKD1, KmURA3, Cas9, gRNA- *TUP1* | CRISPR plasmid to delete *TUP1* | LHZ531 | This study |
| LHZ1133 | pKD1, KmURA3, Cas9, gRNA- *PEP4* | CRISPR plasmid to delete *PEP4* | LHZ531 | This study |
| LHZ1134 | pKD1, KmURA3, Cas9, gRNA- *VPS10* | CRISPR plasmid to delete *VPS10* | LHZ531 | This study |
| LHZ1135 | pKD1, KmURA3, Cas9, gRNA- *HAP1* | CRISPR plasmid to delete *HAP1* | LHZ531 | This study |
| LHZ1015 | *HIS3*, *ARS1*/*CEN5*, *HphMX4*, *TRP1*, KmTEL-filler-KmTEL | KmYAC vector | - | (Wu et al. 2023) |

Duan, J., D. Yang, L. Chen, Y. Yu, J. Zhou, and H. Lu. 2019. "Efficient production of porcine circovirus virus-like particles using the nonconventional yeast Kluyveromyces marxianus." *Appl Microbiol Biotechnol* 103 (2):833-842. doi: 10.1007/s00253-018-9487-2.

Shi, T., J. Zhou, A. Xue, H. Lu, Y. He, and Y. Yu. 2021. "Characterization and modulation of endoplasmic reticulum stress response target genes in Kluyveromyces marxianus to improve secretory expressions of heterologous proteins." *Biotechnol Biofuels* 14 (1):236. doi: 10.1186/s13068-021-02086-7.

Wu, P., W. Mo, T. Tian, K. Song, Y. Lyu, H. Ren, J. Zhou, Y. Yu, and H. Lu. 2023. "Transfer of disulfide bond formation modules via yeast artificial chromosomes promotes the expression of heterologous proteins in Kluyveromyces marxianus." *bioRxiv* 2023.11.30.569359. doi: 10.1101/2023.11.30.569359.

Table S2. Sequences of single-gene cassettes, multiple-gene cassettes, [C4+Down] module and [C4+C5+Down] module

Note: The promoter(s), ORF(s), and terminator(s) were labelled. Restriction sites used to release the cassette were labelled in red. Sequence used for homologous recombination during seamless ligation are underlined.

LHZ1117: P_FBA1_- *HEMA_Sty_* -T_FBA1_

GCGGCCGCGCGCTCCCGATTCCGGAAGTGCTTGACATTGGGGAATTCAGAGCTCACAAGGGCCAAACGTCAATGCTTTGACGTCTCCCGGAGGTATCCCGTTCCAGTGCGTGAGGGGCGTGGAGCGACAAACACACTCCCACATACATCTTTCCCGGAGGCAGAAACAAAATAAGAACAACAACAACACACCAACACGCCAACCATAGTAACCCACACGCTTAAACAACCAGTATCAGACCGGTACCCAACTTCTACGGCCGGCGGCGGCTGGCAGCGGCCTCGAGCATCCTCTATTCTCTCACCTCTTCTCTCGCTGTTGTACAACTTACAGGAATTCCCCTCTCTCCTCGAACTGGAAGCCGTGTTTCACGTGAGTGGTGGTACGATACCCGGTGGGAACCCTGTGTTGTGATATTTCGCCTTCTGGTTTTTGTTTTTGTCTCTCCCCTCTGGCTTCCAATACCGGAAAATTTGAAATTTTGGAAAAACAGGAAATCACAAAAAAACAGGAGAACCTGGAAAACGTAACGAAACCAAATAGTGGAACCCAACTGAATCCGCCGTAGACCAGATCAACCCACATGATTAAACGTCGCCCTCGACTTCCAGTGGTACTTCCAGTACCAATTCCAGTACCACTTTGAACAACTCTCCATAACACTTCCAATTCCACTTACAGTGTATGTGTGTGTATGTGTGTGCGTGTGTGGACAGGTTTCTTGCGCTTCTTGTACTTCTGGTGATGATAGATGGTGGATTCTCGGTACAGGAATTGGCTCAGGCTTTTTTTTTTTTCACATCGATTTCAGTTTGGGCTTTCCCTATGCATTCATTATATTGTGATTGCATTGAGCTTTGGAAATTTTTCATACTCTTCGAAATCATATATAATAAGTTACAATGTGAGTTATTCTCTCCTTCCTGGTTGTTGGTGTAAGCATCATTTAACTAGTCCCTTTTTTTTAAAGTTATTTAATATATATTTTTATTTTGTCCCAAACACCAAGAAGTAATCTTTACTTTTAGCACACAAATAACCAATAAATTCTAAAATGACCAAGAAGCTTTTAGCGCTCGGTATTAACCATAAAACGGCACCTGTATCGCTGCGAGAACGCGTAACGTTTTCGCCGGACACGCTTGATCAGGCGCTGGACAGCCTGCTTGCGCAGCCAATGGTGCAGGGCGGGGTCGTGCTGTCAACCTGTAACCGTACAGAGCTGTATCTGAGCGTGGAAGAGCAGGATAACCTGCAAGAAGCGCTGATCCGCTGGTTATGCGATTACCATAACCTGAACGAGGACGATCTGCGCAACAGTCTGTACTGGCATCAGGACAATGACGCCGTCAGCCACCTGATGCGCGTCGCCAGCGGTCTGGATTCACTGGTGCTGGGCGAACCGCAAATCCTCGGTCAGGTGAAAAAAGCGTTTGCGGATTCGCAAAAAGGCCACCTTAACGCCAGCGCGCTGGAGCGAATGTTTCAGAAGTCTTTTTCCGTCGCTAAGCGAGTGCGGACTGAAACCGATATCGGCGCTAGCGCCGTCTCCGTCGCGTTTGCCGCCTGTACGCTCGCCCGCCAAATCTTTGAATCGCTCTCGACGGTCACCGTACTGTTAGTTGGCGCGGGCGAAACCATTGAACTGGTGGCGCGTCACCTGCGCGAGCATAAAGTACAAAAGATGATTATCGCCAACCGAACCCGCGAGCGCGCGCAAGCCCTGGCGGATGAGGTAGGCGCTGAGGTTATCTCGCTCAGCGATATCGACGCCCGTTTGCAGGATGCCGATATTATTATCAGTTCGACCGCCAGCCCGCTGCCGATTATCGGTAAAGGCATGGTGGAGCGCGCATTAAAAAGCCGTCGCAACCAGCCGATGCTGCTGGTGGATATTGCCGTACCGCGCGACGTTGAACCGGAAGTCGGCAAACTGGCGAACGCTTATCTTTATAGCGTCGATGATTTACAGAGCATCATTTCGCATAATCTGGCGCAGCGTCAGGCTGCGGCAGTAGAAGCGGAAACGATTGTTGAGCAGGAAGCCAGCGAGTTTATGGCCTGGCTACGCGCCCAGGGGGCCAGCGAGACCATTCGGGAATACCGTAGTCAGTCGGAGCAGATTCGTGACGAACTGACTACCAAAGCGCTGTCGGCCCTTCAACAGGGCGGTGATGCGCAAGCCATCTTGCAGGATCTGGCATGGAAACTGACCAACCGCCTGATTCATGCGCCAACGAAATCACTTCAACAGGCTGCCCGTGACGGGGATGACGAACGCCTGAATATTCTGCGCGACAGCCTCGGGCTGGAGTAGGTGTTTCCTCCTTTGGAATGACGCGCTTACTTTACGAAAGTATTAATTTATATATAACAAATTTTATATACTAGAAACGCATGAAATGAATTATGTGAATGGTCTCTTTATTATACCGAAATTGGGCCGCTACCAGCTGTTGCTCCCCTTGCCTATTAATTTATTGGTTTGGTTTGGAAACGGTTGGTGAATTGATTGATTTGTTCAAAACTTTCCCAAAGTGACAGTCACGTGCCGCATATCAGTCGTGGAGAAGTTCTTATAAGCGTCCAACCAGCCAAATAGTCAATTTTAAGTTTGTATTTTTCTGGGGAAAATTCGGATGCCGCGGGAGAGCCTGACCTATTGCATCTCCCGCCGTGCACAGGGTGATTTAAAT

LHZ1118: P_HXT4_- *HEML_Eco_* -T_HXT4_

GCGGCCGCGCGCTCCCGATTCCGGAAGTGCTTGACATTGGGGAATTCAGAGCTCTATTTTTCTGGGGAAAATTCGGATGGGGTGATGAATTCGAGGTTTCTCCACATTGTGTTGTTGTTTTGGTGCCGAAGATGGTAATTACAGCAAAATTAAAAAAAAAAAATGCATTAGATAAAATTTATTATTTTTGTTTGAGCTTTTTTAAGGCACAGATTCTGCCGCACAGGGCGCACACAGGGCGCGCCCGCGCTTACGGTAATAGCCCTGGAAAAGTCCAGGAATAGCCACGGATGGAGAAAAAGAAACAACGGAAAATCTTTGGGGGTGGTTTTAGTAGGCGGTGCACGGGTGGGATCATGCGTTTTTGTCGAGCGATGCCGGAAGAATCTGGGGAAAGCAGAGTTCCCGGCGTGAGATATGGAGTGGAAGCGCGCGTAGACGCGGGCGCAGTGAAGAAAGAGGGAGAGCAAGTGCTGCAGTAAAATAGGAAGTAGCAGGTAACAAGATCTGAGCTGGATTGGTTAGGGTAGTTGTTCCGATACGGGGGTGTCTGCGGGCGAAAAAAAAAACTGGGACCATTACATTGTCCAACAATAGTCACATCACACTATGAAATTTTTTTAACTAATTAGTGTATTGTTCCGGAAATAGTGTGTATGTGTGAGAGTATTTTCGCTTGGCTTACTTGGCTACTTGGCACCGGCTGCTAGATTCTGGTGCCAAGGTGGTGCCAAGGTGTCCAAGAGAAGAAGCGTTAGCAGACGCTAAAGGTGAACGGGTTTTCGCCATGAATTTTCTGATATAAAAGGAAGTGGTTTGGAACTGAAATTTGAAATTGGAATCGTATCTCAATCTATCTCTCTCTGTTATATTTATTATTATTATCTTTTGAATGCTCAGACCCCATCAAACAATAAGAATCACACACACTTAATATTATTATTACTATTAATTTTTTGCGACCACTATTAAAAGTGTCATTATAGTGAAACTGTTATAGGAATAAGAAGACTATAATATTTCCAAACAAAAAAACTCAAAAAATTGATTAAAAATGAGTAAGTCTGAAAATCTTTACAGCGCAGCGCGCGAGCTGATCCCTGGCGGTGTGAACTCCCCTGTTCGCGCCTTTACTGGCGTGGGCGGCACTCCACTGTTTATCGAAAAAGCGGACGGCGCTTATCTGTACGATGTTGATGGCAAAGCCTATATCGATTATGTCGGTTCCTGGGGGCCGATGGTGCTGGGCCATAACCATCCGGCAATCCGCAATGCCGTGATTGAAGCCGCCGAGCGTGGTTTAAGCTTTGGTGCACCAACCGAAATGGAAGTGAAAATGGCGCAACTGGTGACCGAACTGGTCCCGACCATGGATATGGTGCGCATGGTGAACTCCGGCACTGAAGCGACCATGAGCGCCATCCGCCTGGCCCGTGGTTTTACCGGTCGCGACAAAATTATTAAATTTGAAGGGTGTTACCATGGTCACGCTGACTGCCTGCTGGTGAAAGCCGGTTCTGGCGCACTCACGTTAGGCCAGCCAAACTCGCCGGGCGTTCCGGCAGATTTCGCCAAATATACCTTAACCTGTACTTATAATGATCTGGCTTCTGTACGCGCCGCATTTGAGCAATACCCGCAAGAGATTGCCTGTATTATCGTCGAGCCGGTGGCAGGCAATATGAACTGTGTTCCGCCGCTGCCAGAGTTCCTGCCAGGTCTGCGCGCGCTGTGCGACGAATTTGGCGCGTTGCTGATCATCGATGAAGTGATGACCGGTTTCCGCGTAGCGCTAGCTGGCGCACAGGATTATTACGGCGTAGTGCCAGATTTAACCTGCCTCGGCAAAATCATCGGCGGTGGAATGCCGGTAGGCGCATTCGGTGGTCGTCGTGATGTAATGGATGCGCTGGCCCCGACGGGTCCGGTCTATCAGGCGGGTACGCTTTCCGGTAACCCGATTGCGATGGCAGCGGGTTTCGCCTGTCTGAATGAAGTCGCGCAGCCGGGCGTTCACGAAACGCTGGATGAGCTGACAACACGTCTGGCAGAAGGTCTGCTGGAAGCGGCAGAAGAAGCCGGAATTCCGCTGGTCGTTAACCACGTTGGCGGCATGTTCGGTATTTTCTTTACCGACGCCGAGTCCGTGACGTGCTATCAGGATGTGATGGCCTGTGACGTGGAACGCTTTAAGCGTTTCTTCCATATGATGCTGGACGAAGGTGTTTACCTGGCACCGTCAGCGTTTGAAGCGGGCTTTATGTCCGTGGCGCACAGCATGGAAGATATCAATAACACCATCGATGCTGCACGTCGGGTGTTTGCGAAGTTGTGATTTTTCCAACATGCTTAATTCTGTTCTAACTCGTAACTTGTTATTGACTTTTTTCTCTTAACCAAATTAACACATTCATTTCAGAAGATATGCCTTACTGGGATGCTTCATGTCAGTGCTCCTTAGCTTTCTTTTGCTGGAACACGAACGAATAATAATGATAATATCTTTTTATCGTCCTAATATTAATGCTAATTGCAATAAAACTAATGAATGATAATGAACGACTTTTATAAACTGAACATACATTTTATAATATAGACTCTAAAATATATTTCAGTTACTTTCAAGCCACACGAATTTTAGTTTGTTTAGAGATGGACCACCGCGGGAGAGCCTGACCTATTGCATCTCCCGCCGTGCACAGGGTGATTTAAAT

LHZ1119: P_OM45_- *GLTX_Km_* -T_OM45_

GCGGCCGCGCGCTCCCGATTCCGGAAGTGCTTGACATTGGGGAATTCAGAGCTCTTTTAGTTTGTTTAGAGATGGACCATGGACCATGGACGGTTAATGGTTTATATACAGCATGCACCCTTTGCCTTAAAACGGTAAAATGAGATAATGCCTTTCCGTTGTATCCCAGCCAGGCCAACCTCCCAGAGTATGTGCGGAGGGTAACGTCACGTAATGTAGAAAGTATTTCTGTATAGGTAGGGTACCCACCCGCATATGAAATTACTCTGTAGTTCCTGGGTCCCGTCGCCTCCGATATTCTTCTGCTTGATTTCTTTTCTGCCGTTTGCTAGTTTTCTTCAACCCCGGTTACGTTTATGCTTTTTCCTTTTGCTCTAGAAGACGGGTTTTTTTGCTCTTAACAAAGGCCAATCAATGCGATGGACTGGTCAATCTCGGCCGGACAGAGACAAACCCATCGTTCTTGACGATTGGGTTACAGTTCGCAGAGAGAAATGTAGTGGAATGGAAAGAGATCGCAGCAGTATATAGTCAGTTTATTCAGGTTACGCTATGGCATTTTTTTTTTTTGTTACGTAACTTGAGTACGGTGTTAGGTTTCTCTTTCTTTAGAAATTAGGGATGCTTCGGACAATTGGGGTTTTAGAAGAAGGGATACAAAGGAGTAGCATGCGTCGGGTGGTAGAGCTAATGTTAGCTGGTTGGGCTGGCCTGCCGGCTAGAATGGTTAATTGGTAGACCAGGTTTTTTTTTTTTCCCTTACTTCATTCCCTTACGTCTTTTGGGGCGACACAGGGTTTTGTATGAAGAGAATTGCATTTGCTATTAGTTAAGGCTTCTCCGAACCTGTATAAAAGTGGAGAGTTCGCAAGGTATGTAATTATATATACAGGATATATTTCCCTGCAATGCTAGTATTTAATGATGATAGACAGTTGGTTTTACAGGACAAAGATTCGTTTGGTCCATTGATTGAATAGCACACACTTCTGACTACGTAGTTGTTTAACAAGATCGAAGTGAATCTGACTCTAAGAATACAGTATACGAAACAATGTCTGTTTCGCTAGTTATTGGTGCTAAGGCACCAGTTGTCGCTTATCCAGAGTTGATTGCAGCTCGTTTGGTTAATGCTTCTCATCAAAACGCCATTGATATCTCTTTTGTTGAGGATAAGAAAGCCCCAGCTGCCTCTTTCCAAGGCAAAACTGAAAATGTTTTAGCCGAGATTGCTGCTGCATATCCAGAAGTGTTGAAGAATGTGGCTGGTTTCGAAGAATGGGTCGAATTTGGTGCCAACCAGCTTGTAATTAAGAACTTCCAACAACTTGCCTCATCACTTGAAAAGTTGGACGCCCACTTAAATTTGAGAACCTACATTTTGAACACTGTTGAACTAACTTTGGCTGATATTGCTGTCTGGGGTTACTTGAGATCAAACGGTATGGTGGGCTCTATCATCAAGAATAAAGTCTACATCAATGTTTCTCGTTGGTACTCAACCTTGGAATCCATCCCAGAGTTTGGCCAAGCTCATGAATTTTTGACCAAATCTTTGCAAGAAATGAAGAAAGCTGCTAATGTAAACAAGAAGAAAGAAACACACAAGGCCAACTTTGAAATCGATTTACCTGATGCCAAGATTGGCGAAGTCGTTACGCGTTTCCCACCTGAACCATCTGGATACCTACACATCGGACATGCCAAGGCTGCTTTGTTGAATCAATACTTCGCTCAAGCTTACAAAGGTAAATTGATTATTAGATTTGATGACACAAATCCTTCGAAAGAAAAGGAAGAATTCCAGGACTCTATTTTAGAGGATTTGGAATTATTGGGCATCAAGGGTGACAGAATTACTTACTCTTCTGACTACTTCCAAGAAATGTATGATTACTGTGTTCAAATGATCAAGGATGGTAAGGCATATTGTGATGATACTCCAACCGAAAGAATGAGAGAGGAACGTTCTGAAGGTATTCCATCTTGCAGAAGAGAAAGATCAGTTGAAGAAAACTTGAAGATTTTCACGGAAGAAATGAAGAACGGAACCGAAGAAGGCTTGAAGAACTGTGTTCGTGCTAAGATCGACTATCAAGCTTTAAACAAGGCTCTAAGAGATCCAGTTATTTACAGATGCAACTTGACTCCTCACCATAGAACTGGAACTGCTTGGAAGATTTATCCAACTTATGATTTCTGTGTTCCTATTGTCGACTCTTTGGAAGGTGTTACCCATGCATTGCGTACAATTGAGTACAGAGACCGTAACCCACAATACGAGTGGATGCTAAACGCTCTTAACCTAAGAAAGGTCCATATCTGGGATTTCGCTCGTGTTAACTTCGTCAGAACCTTGCTATCAAAGAGAAAGTTGCAATGGTTGGTTGACAAGGATATTGTTTCCAACTGGGACGATCCAAGATTCCCAACTGTAAGAGGTGTAAGAAGAAGAGGTATGACTATTGAGGGCCTAAGAAACTTTGTTTTGTCTCAAGGTCCATCTAGAAATGTTATCAATTTAGAATGGAACCTCATTTGGTCTTTTAACAAGAAGGTTATTGACCCAATCGCTCCAAGACACACTGCTATTGTTTCACCAGTTAAGTTGCACTTGGAAGGTAGCGAAGTTCCTCAAACTCCAAAAATTGAAATGAAATTGAAACATAAGAAGAATCCAGATGTTGGTGAAAAGAAGGTTATATATTACAAGGATATTCTCATCGACGAAGAAGACGCTAAGTTGCTTTCTGAAGGGGAAGAAGTTACTTTGATGGACTGGGGTAATGCTATTATTACAAAGAAAAATGAAGATGGATCTTTGGTTGCAAAGCTACACTTGGAAGGTGATTTCAAAAAGACCAAATTTAAGTTAACCTGGTTAGCCGATACCGATGATAAGGTCGAAGCTGATCTTGTCGACTTTGACCACTTGATTTCCAAAGACAAGTTGGAAGAAGGTGACAACTTTGAGGATTTCTTGACTCCAAAGACCGAATTTCACACCAGGGCTATTGCCGACTTGAATGTCAAGGATATGAAGGTTGGTGATATTATTCAATTTGAAAGAAAGGGTTACTACAGATTGGATTCCTTACCAAAGGATGGAAAGCCTTACGTATTCTTCACCATTCCAGATGGTAAGTCTGTTAACAAATATGGTGCTAAGAAATAAAGATAAGACAGGGGAGAGGGATTCTTCTTCTTACCGACTCAACGCACCACACACCCTCTTTCCCAATTCTAATCATTCGATCTATGTAAACTTATCGTAACTAAACTAACTAACTACCAAGATTCTTCTTTACTTTACAGAGGTGTATTTCTTTAGTTGTCACATACAAAAACGCACACACACACACACATTTTGTCCTGTCCAATGACAAAATGTGACAGGAAACCCGAACGTCGGAGGTTTTCGGGTGGCAGTCATGTCGCCCAGTACTACTCGTAGCAGTAGTACTACTACCATTACGaagaaatgatggtaaatgaaatagCCGCGGGAGAGCCTGACCTATTGCATCTCCCGCCGTGCACAGGGTGATTTAAAT

LHZ1081: P_ADH1_- *HEM1_Km_* -T_ADH1_

GCGGCCGCGCGCTCCCGATTCCGGAAGTGCTTGACATTGGGGAATTCAGAGCTCGaagaaatgatggtaaatgaaataggaaatcaaggagcatgaaggcaaaagacaaatataagggtcgaacgaaaaataaagtgaaaagtgttgatatgatgtatttggctttgcggcgccgaaaaaacgagtttacgcaattgcacaatcatgctgactctgtggcggacccgcgctcttgccggcccggcgataacgctgggcgtgaggctgtgcccggcggagttttttgcgcctgcattttccaaggtttaccctgcgctaaggggcgagattggagaagcaataagaatgccggttggggttgcgatgatgacgaccacgacaactggtgtcattatttaagttgccgaaagaacctgagtgcatttgcaacatgagtatactagaagaatgagccaagacttgcgagacgcgagtttgccggtggtgcgaacaatagagcgaccatgaccttgaaggtgagacgcgcataaccgctagagtactttgaagaggaaacagcaatagggttgctaccagtataaatagacaggtacatacaacactggaaatggttgtctgtttgagtacgctttcaattcatttgggtgtgcactttattatgttacaatatggaagggaactttacacttctcctatgcacatatattaattaaagtccaatgctagtagagaaggggggtaacacccctccgcgctcttttccgatttttttctaaaccgtggaatatttcggatatccttttgttgtttccgggtgtacaatatggacttcctcttttctggcaaccaaacccatacatcgggattcctataataccttcgttggtctccctaacatgtaggtggcggaggggagatatacaatagaacagataccagacaagacataatgggctaaacaagactacaccaattacactgcctcattgatggtggtacataacgaactaatactgtagccctagacttgatagccatcatcatatcgaagtttcactaccctttttccatttgccatctattgaagtaataataggcgcatgcaacttcttttctttttttttcttttctctctcccccgttgttgtctcaccatatccgcaatgacaaaaaaaatgatggaagacactaaaggaaaaaattaacgacaaagacagcaccaacagatgtcgttgttccagagctgatgaggggtatctcgaagcacacgaaactttttccttccttcattcacgcacactactctctaatgagcaacggtatacggccttccttccagttacttgaatttgaaataaaaaaaagtttgctgtcttgctatcaagtataaatagacctgcaattattaatcttttgtttcctcgtcattgttctcgttccctttcttccttgtttctttttctgcacaatatttcaagctataccaagcatacaatcaactATGGAGTCTGTTGTTCGTCAATCTGCAAGGGTTTGTCCCTTCATGAAGTCTGCTACAGGGTCTGTGCAGAATGTGAAGGCTTTGAAAAACGCCAACTTGCCAGCTATTGCGCAAAAGTGTCCGTTTATGGGTCGTGCTATGGAGCAGCGTAGAGCATATGCGTCAGCATCCGGGTCAGGAGCTGGGTCAGGAGCTGGTGCAGCAGCAGCGGCTTCTCCAGCAGCCGTTGAAGCGAGGAACGCAAGTACTGCTTCTGCAGATGCTACTGTTTTGGACCATGCTACCAATGAAGCTTCCTTTGACTACCAAGGATTATTTGAGTCTGAGTTGGCCAGAAAGAGAATGGACAAGTCGTATCGTTACTTCAACAACATCAACCGTTTGGCAAAGGAATTCCCAATGGCCCACAGACAACAAGAGGACGATAAGGTCACTGTGTGGTGCTCCAACGATTACTTGGCTCTTTCCAAGAACCAAGAAGTCATCGATGTGATGAAGAAAACTTTGGACAAGTACGGTGCAGGTGCTGGTGGGACCAGAAATATCGCAGGTCACAACAAGCACGCGTTGCAGCTAGAAGCAGAAATCGCAGCCTTGCACAAAAAAGAGGGTGCGTTGGTGTTCTCCTCTTGTTTTGTCGCTAACGATGCCGTCATCTCGTTGCTCGGCCAGAAGATTAAGGATCTAGTCATCTTTTCCGATGAGCTGAACCATGCCTCCATGATCGTGGGTATTAAACACGCCTACACCAAGAAGCATATCTTCAAGCACAATAACTTGGAAGAATTGGAAAAATTGCTAGCCATGTACCCAAAGTCCACTCCGAAATTAATCGCCTTTGAATCTGTTTACTCTATGTCTGGGTCCGTCTCTGATATCAATAAAATTTGCGACTTGGCAGAAAAATACGGTGCCTTGACTTTCTTGGATGAAGTTCACGCAGTTGGTTTGTACGGTCCACATGGTGCCGGTGTTGCAGAGCATTGTAACTTTGAAGCTCACCGCAAAGCTGGTATTGCATCCCCAGAATTCCGCACTGTCATGGACCGTGTCGACATGATCACCGGTACCTTGGGTAAATCTTTCGGTACTGTCGGTGGTTACGTTGCTGCTTCCTTAAAATTAATCGATTGGCTCAGATCTTACGCACCAGGATTCATCTTTACCACATCTCTACCACCATCCGTTATGGCGGGTGCCGCAGAGGCTATCAGATACCAACGTTCTCACTTGGACCTAAGACAAGACCAACAAAAACATACAACTTACGTCAAGGAGGGATTATCCGATCTGGGTATTCCAGTGATGCCAAACCCATCCCACATTGTCCCAGTTTTGGTTGGTAACCCTCATTTGGCTAAAGAGGCTTCGGATATCTTAATGCATAAACATCGTATCTACGTTCAAGCAATCAACTTCCCAACGGTTTCCAGAGGTACCGAACGTTTAAGAATTACTCCAACTCCTGGTCATACTAATGATTTGTCTGACATCTTGTTGGATGCTATGGAAGACGTTTGGTCAACCCTACAATTACCAAGAGTTCGTGACTGGGAAGCCCAGGGCGGCTTATTGGGTGTTGGTGATCCAAACCACATCCCTGAACCAAACTTGTGGACTGAAGAACAATTATCTTTGTCTAACGATGACTTGAACCCAAATGTCAAACATCCAATAATAGAACAATTGGAAGTTTCTTCTGGTATTAGATATTGAgcgaatttcttatgatttatgatttttattattaaataagttataaaaaaaataagtgtatacaaattttaaagtgactcttaggttttaaaacgaaaattcttattcttgagtaactctttcctgtaggtcaggttgctttctcaggtatagtatgaggtcgctcttattgaccacacctctaccggcaTGAATAATGAATGGCCTTGTATTCGCCGCGGGAGAGCCTGACCTATTGCATCTCCCGCCGTGCACAGGGTGATTTAAAT

LHZ1110: P_PDC1_- *HEM2_Km_*-T_PDC1_

GCGGCCGCGCGCTCCCGATTCCGGAAGTGCTTGACATTGGGGAATTCAGAGCTCTGAATAATGAATGGCCTTGTATTCGTTTTTTTCCGAGAGAAAATTAACAAGAGCGAAAAAAAAAACGGGCTTCGGTGAAAATCGGGTGAATATGCAACTAGCGGGACGAATGCTCTGGAAATGCATATCCTATGCAACTAGCGGGATGAACAAATCTCACCCCAGAATTCGCAGGAAAAAACAGGAAAAAAAAAAAGAAGGCCACCACGGCCACAAAGACCACAAAGGCCACAAAAAGAACAAAAAAACAACCGTCCCAGCTTCCAGTGTTTGGAATACTGGAACACAGGAAGCCGCATAAGAGTGGGCGTTGCACAGGAAGCCAGGCCCAGAAGCCCCAGAGTTACTTTTTTTTTTTTGTTTTTTCCTTCTGTTCGCTGTGCCCGCATCAGATGATGCGCCTTTATTTACGATGCCAATGCGAATAGCACCAGTGAGAGCACCAGTAAAAGCATACGCATACACATACACACATAGAGCAAGCAAGCAGGCTAGCAACCAGGAAAGGCTGCCAGTGACTGCTACTGGGTGTCTAAGAACCGTAGGGCGGATTATTGTTGCGGTGGTTGGTTGCGGGTGGTTATGCGATGGTACGGTGCAGAATCGTACGGTGTTGGTTATGGAATTAGTATGGGTATGTGATATGTGGTAATATGTGATATTGGGTTATTGTGATTTGGAATACTGAATATCGAATATGGGATATGGAATATGGCCATGGCATGGTATGGTATGGGATGGGAGTATTCTATTTTATTTTATTTTATTCTGGTTCCTGCGTTTAGGGTAGGGTAGGAAGAAGGTGAGTGCTTTTGTATATAAGTGGAGTGTCTGGATCAGTTTTGTGGATTGTGAATGTTAGTTTCCCCTTTAATGTATATTTGTATTATTTGCTTTTGAGTACTCAATAACCAAGCACAACTACTAGTTTTAAAGGATCCATCCTCTTAAACAGTACAAATCGCAAAGAAAAGCTCCACACCCAAACCAAATAATTGCAATGGTTCACGTTGCAAAATTTTTAGACGAACGTCCTACTGAAATTCCTTCCATCCTTGCTGGTGGATACAACCACCCTCTATTGAGAGAATGGCAGAATGAAAGACAGTTGACTAAGAGCATGTTCATTTTCCCGCTATTCATTAGTGACCAGGACGAGGAAGAGACCCTGATACCATCGCTACCAAACATCAAGAGATTTGGTATCAACAAATTGAAGGATTATGTTGCTGGTTTGGTTGCCAAAGGTTTACGTTCGGTGATCTTGTTTGGTGTTCCATTGAAGCCTGGTGCTAAGGACGAGGTTGCAACCGCAGCTGACGATCCAGATGGGCCTGTCATCAAGGCTATCAAGTTGTTGCGTAAGGAATTCCCAGATTTGTACATTATCTGCGATGTGTGTTTGTGTGAATACACTAGTCATGGTCACTGTGGGATCTTGTATGAAGATGGTACTATCAACAGAGAAAAATCTGTGCAAAGAATTGCTGCTGTTGCTGTGAACTATGCGATAGCTGGTGCGCACTGTGTTGCCCCAAGTGATATGATTGATGGTAGAATCAGAGAGATCAAAATGGGATTGATCGAACACGGATTAGCCCACAAGACGTTTGTTATGAGTTACTCTGCCAAGTTTAGTGGTAATTTGTACGGTCCATTCAGAGATGCAGCATGCTCGCAACCAGGTCAAGGTGATCGTAAGTGCTACCAGTTGCCTCCAGGTGGTCGTGGTTTAGCCAGACGTGCATTGAAAAGAGATTTGGCTGAGGGTTCCGATGGTATTATCGTCAAGCCTTCGACCTTTTACTTGGACGTTATGTCCGATGCTGCTGAAATTTGTCGCGACATTCCAGTTTGCGCATACCACGTCAGTGGTGAATACGCAATGTTGCATGCTGCTGCTGAAAAGGGTGTTGTTGACTTGAAGAGCATTGCTTTTGAATCCCACTACGGTTTCTTGAGAGCTGGTGCTCGTTTAATCATCAGTTACCTCACCCCTGAGTTTTTGGAATGGTTAGATGAAGTGAACTAAAGAGGGAGAGGATAAAGAGATAAATTACGATTTTGGATTTTAATGATTTTATAAACAACAACAACCAACCAGCCTTTTACTTTATTTGGCATATACACAAGCTTACTCCATTTCATTGATTATCTATGTGTATATATATAAGTGATGTATAACAATTATTATTATACATAGATAATATTTTTATGATATGTTTTTTCTGAGTTTTGATATTATTTATTACAAGTTACAAGTTACAAGTTACAAGTTACCAGGAAGAATTAAATAAAGGTAAATTGGGGGAATTATAAGCGTATGGGCATAGCCTTGTCCAAGGCCTTGGCCTTGGCCGCGGGAGAGCCTGACCTATTGCATCTCCCGCCGTGCACAGGGTGATTTAAAT

LHZ1111: P_PGK1_- *HEM3_Sc_* -T_PGK1_

GCGGCCGCGCGCTCCCGATTCCGGAAGTGCTTGACATTGGGGAATTCAGAGCTCGCCTTGTCCAAGGCCTTGGCCTTGGTCAATGCCCTTATTCTCTTGTTTCCATTGCCTGTCGATATACGACTTCAAGGCCATGTACCTGAGATCCTCATCTGGGGCCTGAAGATCCATTGGTGAGTGCCACTGGCTGCTGTGCCGTTGTGAAAAGGCCCTGTGTAGCAGAGAGGGAGGTGTGTATAGGTATATATATGTATATATATGTATATAAGTGTGGATTTAACACACTCTCTCTTCTTTCCCATGCCATGCCAGTCACGTGTTCCACACGTGACTGTACTTACCCTCACATTACCCTCACTCTTTCACATTACCCTCCCCATCCCATCTTCTTTGGTTTTCCCCCCCAGCTTCCTGTCTTCCACTATCCCTGCAACCACCACCACCACACAACAAACATCCCCCAGCAGCCCCCCAGCAGCCCGGAAAAATCCACGCCTCCCCCCGAACACACGAAGCCCGCCCCGGCTTCCACTCCAACACTGCCAACACTGGAACCCCGCACCACGTAACCACCCACTTTAGTGGCTGCCCGCCCCTCCTGCGCTTCCCTGGGGCTTCCCTTGCGACCACACTCCGGTGGCATGGCCCTCGCTTGCTACATGCTACCTTTTTTTTGCCCTTTTCTGGCCTTTTCTGGCTTTTCTGGCCTCACCACAGTGTACCACAGTGTAGTAGTAGTGTAGCATTGTCGAGTGGTATTGTCGAATGGTATTGTAGCCTGTTGTAGCCTGTTGTAGCCTGGTGTAATGTCTGGCATAGTCTGGTGTCTGGTGTCTGGTGTCTTTTGGAAACTGAACAGTGGAAAGTGGAAGTGAAAAATTGTATAAATATAGGTGTCCCATTCGTGGTTTGGTGGATGTGTCCTTGACATTGCAGGTTTCTCTGTTTCGCTGTGTTAAGACTTGTTAACACTTGATAAGTATTTCCCTAGTAATACCCCACCAACCATCGGTTGATACATTTTAATACACACTACCTATATAAAGATACAAAAATGGGCCCTGAAACTCTACATATTGGTGGGAGAAAATCGAAATTGGCGGTAATACAATCCAACCATGTTTTAAAACTGATCGAAGAAAAGTATCCGGACTACGACTGCAAGGTTTTCACTTTGCAAACTCTTGGTGACCAGATTCAATTCAAACCTTTGTACTCATTTGGCGGTAAAGCTTTATGGACAAAGGAGTTGGAAGACCATCTTTACCATGACGATCCCTCAAAGAAGCTTGACTTGATCGTTCATTCTCTGAAGGACATGCCCACTTTACTACCAGAGGGTTTCGAGCTGGGGGGTATCACTAAGCGGGTCGATCCAACAGATTGTCTTGTCATGCCCTTTTACTCTGCTTATAAGTCTCTGGATGACCTTCCAGACGGGGGGATTGTGGGAACCTCATCCGTGAGAAGATCTGCTCAGCTAAAAAGAAAATACCCACATTTGAAATTTGAAAGTGTCAGAGGAAATATACAAACTAGATTACAAAAACTAGACGACCCAAAATCTCCGTACCAATGCATCATCTTGGCGTCTGCTGGGTTGATGCGTATGGGGTTGGAAAACAGAATTACGCAGCGATTCCATTCGGATACAATGTACCATGCAGTTGGACAAGGCGCCCTGGGTATAGAAATTAGAAAGGGTGACACCAAGATGATGAAGATTCTTGACGAAATTTGCGATCTAAATGCAACTATATGTTGCCTTTCGGAGCGTGCTTTGATGAGAACTTTAGAGGGGGGTTGTTCCGTTCCTATTGGTGTGGAATCTAAATACAATGAAGAGACTAAAAAATTACTATTAAAGGCCATTGTAGTTGACGTTGAAGGCACAGAAGCAGTAGAAGACGAAATTGAAATGCTAATAGAAAATGTTAAAGAAGATTCCATGGCGTGTGGTAAGATACTAGCTGAAAGAATGATTGCCGATGGCGCAAAGAAAATTCTGGATGAAATTAATTTAGACAGAATCAAATGAGTGCATTGTTGAACTTTCTAATGAAACCACACGTGAATTAAAGGAAAGCGCGCAGCAGCAGCAGCAGCAGCTTTTCTTTCCTTCTTCAAATCATACCTTTTCTCTATTTCAACGTTCTTTTCATTTCACCATGAAATGAGTTGAATTGTTGCTTTGAGTTTACGACGATACATAAATATTAATTAAAATACGAAAAATACACATTTTATTTACGACACTTGCCTCTCCCCCTAGATGTTACGCTGCTCAGGTTCGAGCCGCTCGATCCCTAGCATATCGAGAACTTCGTACTCGTCCCCCATTTTCAATTTCCCAAAAAAACAAGCCGCGGGAGAGCCTGACCTATTGCATCTCCCGCCGTGCACAGGGTGATTTAAAT

LHZ1112: P_ENO2_- *HEM4_Km_* -T_ENO2_

GCGGCCGCGCGCTCCCGATTCCGGAAGTGCTTGACATTGGGGAATTCAGAGCTCATTTTCAATTTCCCAAAAAAACAAGAAGCCGGAATCCACCGGAATCCACTAATACGCAAAAAAGACGACGCCAGCTTCGCCGTTGGGCATTGGCACCATATTCCATCGTTCGTTTGACTTTGGTTTTGGTTTTAGGTTCTGGCTTCGTGTTTTCCCCTCACTGCCCTTGTCCTTGCCGTGCTCCCCCTCGTTCTCTGCGCAAAGTCTGGTGACCCTCCGGCTGCCACAAAATCCCGAGGCACTGAAAGGGTGGGGCCTCGGGGGTCTCGGGGCAGAAGCCTTGAGCTTCGTGTTTATTCACTCTGTACGGGCGGTTCTAGCGTTTCTGGGAATCCAAAGAGGAAGGGCCGTGTTTCGTGCAGGCATTTTAGTGGATGCCCGGGATGTAAGCTCTTCGGTTACAGAAAATCAGGTTGTGATTCCAGTATGAGGAATAGTGGATGCCAGGTTGTGCGAGCATCACCAATATCATGGGAAATGCGAAATGGGCATGAGCCCAACCAACACAACACAATACCATACGCCAAATGGTAGCCTGAAAAAAAAAAAAAAGGTCTTGCCAGACCACTAAACTCGAGGTAGAACAAGACTGAGAAAGAGTGTGTGATCCCTTTGGTGGTAGTAATTTTTTTTTTTTTTTTTCAAGTTTCCAGCATCCCAAACCGAAACCCAACATCATCATGATCCCATCCCATGCCTATGCCCATGCCACATTCATTCATTGCACAACACACAACGTAGTGGACGACAGCATAGCATCAGTTAACTAACGGCCACTTGTTTTTTTTTCTTCCATTTTTTCGTTTTGTTCTAGCAACAATGAGTTCTAATTTTTTTGTTATAAAAGGGACAGTTAAGGTATATGTAAGGTTTCTTGTTCCATTTGGGTTAGAGTTTTGTAGTATTAGTTTTGGTTCTTAGTTCTTTTGGCCTAAGAAACCAGTAAGAATTCTTCTGATTTTCTTTACAAACACATCAACAACAACAAACACAAACTACAATGTCCAGAATAATTTTTCTTAAGAACAAAACAACACCTACAGACCCGTACGAAACCACTTTTACAGATGGAGGGTTTGAACCTGTATTTGTTCCACTAATACGCCATTTCCATGTTCCAGACGAAGCGTTGACGCTATTCAGAAACAGGAGCTACCTAACGAAATTGAAATACATTATAGTTACTTCACAACGAACAGTCGAATGTCTAAATGAGTCAATACTTCCTAAAATGACTACGGAAGAGCAATCTCTTTTAAAGGAAAAGACTATATACACAGTTGGACCGGCCACTTCGGAGTTCTTGAGAAATTCTGGTTTTCAAAACATAAGAGGAGGAGTCGAAGTTGGAAATGGCGGATTACTTGCTGATCTGATTGTTAAATCGCATTCTGAGGAAGAGATTGATCACTTTTTGTTTTTAGTTGGAGAAATAAGAAGAGATATCATTCCCAAGAAACTTAAAGCTAATGGCTACAAGGTCAACGAAATTGTAACTTACAAAACCGAGAATCTGTTAGACAATATTGATCGTTTTATTTTGCACTATAAGAATGATGAGAATAACCAAGCGGTTATGAACAAGCCCTCCACATGGATTGTGTTCTTTAGTCCCCAGGGTACGGAAGATATAATTGAGTACCTAAAAGAGAACAAGGGCTATAAAATTGCTAGCATAGGTCCTACAACGGAAAAGTACCTCCTAGAAAAAGGTTTGAAACCAGACACTGTTAGTTTTAAGCCGTCTCCAATATTCTTACTGAATGCCATTAATAGTTACAAATAAATCTAGCTGACGCGACGCTTTTTTTCTCCAACTAAGGGAATCCAGGGAAACCCCCCTCGAGCCCCAGTTTTGGTTAGAAATTGTCCTTTTTTATACTATTGAATGACCTAACTAAATCTATATAAACCTTTTTAACGTCTAACGACTCTTTATTATTGAAGTGGGAAGTCTTGGGATGGGAAGTCTTGGGATATTTCAGGCCTTTGGCCAGGCCAAAGGCCAGGCCTTGGCCCAAGGTACCACCCCCTTATATATGCTCAGCCAATTGACTCAGCTGTCCCTCCACACTTTATTCCTGTAATCCGAAAAGGTAAACAGACACAAACCGCGGGAGAGCCTGACCTATTGCATCTCCCGCCGTGCACAGGGTGATTTAAAT

LHZ1113: P_INU1_- *HEM12_Sc_* -T_INU1_

GCGGCCGCGCGCTCCCGATTCCGGAAGTGCTTGACATTGGGGAATTCAGAGCTCATCCGAAAAGGTAAACAGACACAAAAACGACAAGAGAAGCAAACACAAAAAAAAAAAAAAAAAAAAACAAAAAAAAAAAACACAAACACAAACACAAACACAAAAACGCTAAATTATGCACACAAGGGCCGGCGGGGCTGCCGGAAAAAAAAAGGGAAAAATACACAGACGAGCGCGCACAGATGGGGTTACCACTGCAAGTTACAAGTTGCAAGTTGCACGCTGGAATCAGAATTGGAATCAGAATTGGAATTGGAATTAGAATTAGAATTAAACTTGGGGTAGCCACGGGAACGGGATAACTCAGGAATCGCTCGCAGGCGTCTCCGTCTAGGCAATCCCAAGGTAAGCCTAGGCACTCCCACAGGGGAAAGAACGGTTGAAGGCAAAGTAGTGCTAACAATTGGTAACGAATGGTAACAAGTGTGTCCGTCTCCACCTGACATTTGCTAGAGCTGGGGATTCCACATTCTTGTGCTCTGAATTCTCAAACCGAAATGGGGCGTTGTTACCCCAGGTATCCGGTTGTAGTTGGCACTGGGGATGGAAAAAAATGATGTTGATGTTGAGTTAGTTGGGTTGAGTCAATTAGTGCGTGAAAGTATCACCACTTTTGTCATCCGGCGTTTCTGTGCGAATCACACACACACACACAGTTTATTGGAGCACTTGTTTCTGGCGTATTCGTAATTGTTCTGCGGTGCGGTTCTGTGTGCATTTTTCCTGGGGTGTCTGCCGCACCTACTCATCACCCACGCCGTGGGTTTGAGCCATGGCGGAGGTACGACTGACTGGCTGCCTGCCTGCCTGACTGACTGCCTGACTGCAGGAAAAGAGGGTTTCGAAGGAAAAACTTTTCCTGTGTTAATCCGGCCGTGCGCCGCTGCTCCAAAATCCACCTTCATGAGAAGGAGTTTGAAAAAACAAAAAAATTCACATATAAAAAGCGTATCTCGAGATCTCAAAGTCTCCCTTGAATCGTGTTTGCCAGTTGTAACTCATCCTTTATTCTTCTATTCTATCTCTCTCTTTCCTTCCCCTAATCAGCAATTAAATCCGGGGTAAGGAAGAATTACTACTGTGTGTAACGGTTATATTTCGTTTTTTATTTTTTTTTTCCATTGCCATAGAGAAAGAAAAAAAAAAAAAAGAGAGTTTGTGAAGATCTTCCATTCGAATCCCATAAGTGACACATTTAATTTTTTTTTTGTTAGATATGGGTAACTTTCCAGCTCCAAAAAACGATTTGATATTGAGAGCCGCAAAGGGTGAAAAAGTCGAGAGACCGCCATGCTGGATAATGCGCCAGGCAGGTCGTTACCTGCCGGAATATCACGAGGTGAAAAACAATCGTGATTTCTTTCAAACTTGCAGGGATGCGGAAATTGCTTCTGAGATTACTATCCAGCCGGTAAGACGCTATAGAGGCCTCATTGATGCTGCTATTATTTTTAGTGATATCTTAGTTATTCCGCAAGCCATGGGTATGAGGGTCGAGATGCTCGAAGGTAAAGGTCCACATTTCCCAGAACCTTTAAGAAATCCGGAAGACCTCCAAACGGTATTAGACTACAAGGTTGATGTTTTGAAAGAGTTAGATTGGGCTTTCAAGGCAATCACCATGACAAGGATCAAGTTGGATGGTGAGGTTCCCTTATTTGGCTTTTGCGGGGGACCTTGGACTCTAATGGTTTATATGACGGAAGGCGGTGGATCCCGTCTTTTCAGATTTGCCAAACAATGGATTAACATGTATCCAGAGCTTTCTCACAAATTATTACAAAAAATCACTGATGTGGCCGTGGAGTTTCTGAGTCAGCAAGTCGTGGCGGGTGCTCAAATACTACAAGTTTTTGAAAGTTGGGGTGGTGAGCTTTCGTCTGTAGATTTTGATGAGTTTTCCCTACCATATTTAAGACAAATTGCCGAAAGAGTGCCTAAAAGATTGCAAGAATTAGGTATCATGGAACAGATTCCTATGATCGTTTTTGCGAAAGGGTCGTGGTATGCTTTGGACAAGCTATGCTGTTCAGGATTTGACGTTGTTTCGTTGGACTGGTCCTGGGACCCAAGAGAAGCGGTAAAAATAAACAAGAACCGTGTCACCTTGCAGGGCAACCTGGATCCTGGCGTCATGTATGGTTCTAAAGAGGTAATAACAAAGAAAGTTAAACAGATGATTGAGGCTTTTGGAGGTGGGAAGTCCCGCTACATTGTTAATTTCGGTCACGGTACCCACCCTTTCATGGATCCAGACGTCATCAAGTTTTTCTTGGAGGAGTGCCACAGAATTGGTTCGAAGTAAGGCCGCAAGCTTTGATCTGATCTGCTTACTTTACTAACGACAAAAAAAAATCAAAAAAAAAAAAACAATCAGTCCTTCTCTTCTTACGATATGATATGATTAAATGATGCTATGAAATCATCTTCTTCTTAACTTTCTTAAATCTTACGCGTCACTTACTCTATATACCCGTTTAGCTTTGCCTGGTCACAGCGACATTTTATATAAGTGTACGTATTTTCTTTTTTTTTTTAAAAATTTCTATTCTAACCTTAGAAAAGTGCCCTTTAAACCAGCTGTCCTGGCACTATATCTTTATCATGTGCCGGTCGCTTTCCCTTTCCGTTTCCCTTTTCCTTTCAATTGGTGGCCTGGAATTCCGAACTCATTTTCGCATCTGAAACTAATTCTCGAAACCTTTAACATCAAACAATTGAAAAGATCATCATCACCAGAAATAAGAAAAAGATCAACACAACAGCTAATAACAGTACGAAAGAAAGATCGCTCGAGTGAAAAGGCAGCCAAGAAAGGTCATTCGATTTGGGTCTAGACTGATTATAGACATACCAATTGCACTCAGTAAGAAAATGAGTTTCAAATTTGACGATGACGGTGTGGTAAAAGAATTTCACGGCAACACCATCATATGCCATATTCCTCAACAAACCGAATTCTTCAACAAATTGTTGGACTTCTACCGTTTTGCGAAACGACTTTCCTTCTACGACAAGATCACCCTACTTCCTCCTTCAAGCTACCACGTTACGATCATGAATTGCTGCCACGAACACGATCGTTCTGAGGGCCACTGGCCCAAAGGAATCGATCCGGACACAAGCATGCTGCGGTGTACATCACATCTGACCAACATTCTAGGATCGGTGCCAGGTGAATGTACGTAATGGAGTCCGCGGGAGAGCCTGACCTATTGCATCTCCCGCCGTGCACAGGGTGATTTAAAT

LHZ1114: P_AFT1_- *HEM13_Km_* -T_AFT1_

GCGGCCGCGCGCTCCCGATTCCGGAAGTGCTTGACATTGGGGAATTCAGAGCTCGCCAGGTGAATGTACGTAATGGAGTTTGGATAGATTCGATACAGTTCATTACCAAGAATGGACAGCAGTCCCCCATATACGGGAATGCAACCGGAGGATCATTGAACCAGATTAGCATTGTCGCTCCATACAACCTTAAAGGGTTCTTTGGTAATTGTTCGAATTGGATGGACAGTCTTGGAGTGTTGTACGGGTAATTGGAGCCTGTTTGAAGCGTTTGTTTCCCTTTGGAGCGGTTTCCGTGTCATCAAGATGTCGGGGTCCGTCAAAAAAAATTTAGCGGGCACTCCGGGGTCCGTGGGGTCCGTGTGGTAGGGTGTGCCAGACCCGGTACTCTAAATCCATCCCAAAGAGCAAAGAAAGGGTTGCATGAATAATGGACCCTTGTTGCGAGCAGTTACTGTTGCAAGCAGTTACTGTTGATAGAGATGGTGTTCGCTAGTATACAGGTCAAATACTTAAGGTTATCCTTTGTGTGGGAGGATTTTAGATTACCGAGTCTTTTTTTAGTGGAGTGCAGTGGGATGAGAAAAGTTGGCCCGACCTCGGAGAATTTCTCGTAATATGTTGCTCAATCTATTAAAAAAAAACTCGGATACTTCGAAAGCACCCCGTGTGGTTATCCCATGCGCGTAAAATTCACCCTCTGCGAATCGAGGGCGCCCCTTCAGAAACGGGATAACCGGGTGTTTTAGCCCAGGGCAATATCCAATAGGGCTAACCTAGTCGCTTTCCGAGCCCAATTTGTTAGTCTTGGCGCACCCCGATTGCTTGACACGGAGTCCGTCAATACCTATAAAATTGGGATTTAGAGGTTAATTATTTTTTAGGAATCTGTAACCACACCCCTCTTCGGACCCCGCCTAGTATTTTCTTCTCATGCCCGAAATAAATAAAAGGACAATTCTCCCCCAGAATATTACTACTTGTTCATTGTTTTAAAATTCCAACCAGGCTTCGTTACTCCTAAAAAATTTTCGAACCACGTACACCTTCAGCAATGACAGACTCTGCTGTGCCAATGAGAAAGAAGATGGAAGAGTTGATTCGCCGTAAGCAAAAGGAAATCACTAAAGGGTTGGAGGAATTGGACACTGTGCAGTTCCGTGCAGACTCCTGGACCCGTGGTAACGATGGTGGTGGTGGTACCTCTATGGTTTTGCAAAACGGTTCTACTTTCGAAAAAGGTGGTGTCAATGTGAGTGTTGTTCACGGTACTTTGACTCCTCCAGCTATTAGAGCGATGAAAAACGATCACAAGAACTTGCACTTGCCAATTGACCCAGAAACTGGCGAGCCAGATGCTTCTGGTGTCAGATTTTTCGCTTGTGGTTTGTCGATGGTCATTCACCCAATCAACCCACACGCGCCAACAACGCATTTGAACTACAGATACTTCGAGACGATGCATGCGGACGGGACACCTCAGGCCTGGTGGTTTGGTGGTGGTGCCGATCTTACCCCATCGTATTTGTACGAGGAAGATGCAAAGTTGTTCCATCAATTGCACAAGGACGCTCTAGACAAGACAGACGTTACCCTATATCCAAAGTTCAAGAAATGGTGCGATGAGTACTTCTACATCAAGCACAGAGAAGAAACCAGAGGTATTGGTGGTATCTTTTTCGATGACTTGGACGACCGTGATCCAGACGTTTTGTTGAACATGGTCGAAAACTGTTTCGATGCGTTCTTGCCCTCTTACACGGAAATCATCAAGAGAAGAATGAACATGCCATACACCGAGGAGGAAAGACAATGGCAGCAGATTAGACGTGGTAGATACGTCGAATTCAACTTGGTCTTGGATAGAGGTACTCAATTTGGTTTGAGAACTCCTGGCTCTCGTGTTGAGAGTATCTTGATGTCCTTGCCTGTTACCGCCTCCTGGTTGTACGACCACCACCCTGAACCTGGCTCCAGAGAAGACAAATTGCTACAGGTCTTGAAAAACCCAATCGAATGGGTGTAATGGGTGCTTAAGATCTGTCTTTTTTATTTATAATCGGCCCTTTTATCACTTTATTTTTTAGTTTATTTATTACATCTTTCGTCTGTATACTAGTTTGGGTCAAAGAAGTCAAAAATCGCACAATTTGGGTGTAGAACGTAAACGTAGTTAACACCTTGCGCAGCCTCTGCATCAGCGTCTTCGAGATCTTGAACATCTTGTTCGTTGGGTGCAACTTTCATCAAGATCTTGTCATGTACCAATTTACTGAGAACCTTGTGCACCAACTTTCTTTCAGCCCAATATTCTTCCTCTGTATGAAGCTTGCCTTGTCCCCGCCGGGTCACCGCGGGAGAGCCTGACCTATTGCATCTCCCGCCGTGCACAGGGTGATTTAAAT

LHZ1115: P_TEF_- *HEM14_Sc_* -T_TEF_

GCGGCCGCGCGCTCCCGATTCCGGAAGTGCTTGACATTGGGGAATTCAGAGCTCAGCTTGCCTTGTCCCCGCCGGGTCACCCGGCCAGCGACATGGAGGCCCAGAATACCCTCCTTGACAGTCTTGACGTGCGCAGCTCAGGGGCATGATGTGACTGTCGCCCGTACATTTAGCCCATACATCCCCATGTATAATCATTTGCATCCATACATTTTGATGGCCGCACGGCGCGAAGCAAAAATTACGGCTCCTCGCTGCAGACCTGCGAGCAGGGAAACGCTCCCCTCACAGACGCGTTGAATTGTCCCCACGCCGCGCCCCTGTAGAGAAATATAAAAGGTTAGGATTTGCCACTGAGGTTCTTCTTTCATATACTTCCTTTTAAAATCTTGCTAGGATACAGTTCTCACATCACATCCGAACATAAACAACCATGTTATTACCATTAACAAAGCTAAAACCGAGAGCAAAAGTTGCTGTTGTAGGGGGTGGCGTTTCTGGACTATGTTTTACTTATTTTTTAAGCAAGTTGAGACCGGATGTTGAAATCACACTGTTCGAATCGCAGAACAGAACTGGGGGTTGGATATATTCTTGTAACACAAGAGATATGAGTGGGAACCCAATTATGTTGGAGAAGGGACCCAGAACATTGAGGGGCGTATCAGACGGGACCGTTCTGATTATGGATACCCTTAAAGACTTGGGCAAGGAAGCAGTTATTCAAAGCATTGATAAAGGTTGCATTGCAGACAAAAAGTTTCTACTAGACCCCAGTGATAAACTCGTGCAGGTTCCTAATTCGATATCTACAACAGTAAAATTTCTGCTGAATCCGTTGGGAAAAGGACTCATCACGGGTATGATGGGAGAATGGTTCAGAAAAAAATCTCCACATCCTGGCCAAGACGAAAGTGTCGAATCCATTTGTGACAGAAGGTTTGGGAATAACTACATATCAAACAATATGATCAGTGCCTTACTAAGAGGTATCTATGGGGATGACGTTTCCCTATTAAGTGCCAAGAGAACGTTTAAGAAAATATACTACAATGAACTAAAGCACGGATCTAACACACAAGCTATGATTGATAATATGCGCGGAAAGTCAAGAAGTAAAAAAACTGAGAACCTGCACCAATCTTTAACTGGCTGCCTTAACGACTACTCAAATGCGTTTGGAAAAGACAGGTCAAAGTTATTAGACTTATCCAACACGCTAAAGAAATATCCCATGTTGGGTCTTGCTGGGGGACTAGAAACGTTTCCCAAGATAGTCAGAAATGCTTTGAACGAATTTAAAAACGTCAAAATAGTTACTGGGAACCCGGTTACGCAAATAATGAAACGCCCTGCTAACGAAACGACAATCGGATTGAAAGCGAAATCTGGCGACCAATACGAAACATTTGACCATTTAAGACTTACGATAACACCTCCCAAAATCGCTAAATTGCTACCGAAGGATCAAAATTCATTATCCAAGTTATTAGATGAGATACAATCAAACACAATAATTTTAGTTAATTATTATTTGCCAAACAAAGATGTAATAGATGCCGATCTACAAGGCTTTGGATACTTGGTTCCCAAATCCAATAAGAATCCAGGGAAATTGCTTGGTGTAATTTTCGATTCTGTTATCGAAAGGAATTTCAAACCACTTTTTGACAAACTCTCCACAAACCCAAACGCCCTCAACAAATATACAAAAGTGACTGCGATGATAGGAGGTTGTATGCTCAATGAACACGGTGTTCCTGTAGTGCCATCCAGGGAGGTAACCATTAATGCAGTCAAAGATGCGCTGAACAACCACCTCGGCATCAGTAACAAGGATCTGGAAGCTGGTCAGTGGGAATTCACTATCGCCGATAGATGTCTGCCAAGATTTCATGTAGGTTATGACGCATGGCAAGAAAGAGCTGAAAGGAAGTTGCAAGAATCTTACGGCCAAACAGTTTCTGTGGGGGGAATGGGATTTTCTAGAAGTCCCGGTGTCCCTGACGTTATTGTAGACGGCTTTAACGACGCCTTACAGCTAAGCAAATAATCAGTACTGACAATAAAAAGATTCTTGTTTTCAAGAACTTGTCATTTGTATAGTTTTTTTATATTGTAGTTGTTCTATTTTAATCAAATGTTAGCGTGATTTATATTTTTTTTCGCCTCGACATCATCTGCCCAGATGCGAAGTTAAGTGCGCAGAAAGTAATATCATGCGTCAATCGTATGTGAATGCTGGTCGCTATACTGCTGTCGATTCGATACTAACGCCGCCATCCAGTTTTTAGTTTGTTTAGAGATGGACCACCGCGGGAGAGCCTGACCTATTGCATCTCCCGCCGTGCACAGGGTGATTTAAAT

LHZ1116: P_OM45_- *HEM15_Sc_* -T_OM45_

GCGGCCGCGCGCTCCCGATTCCGGAAGTGCTTGACATTGGGGAATTCAGAGCTCTTTTAGTTTGTTTAGAGATGGACCATGGACCATGGACGGTTAATGGTTTATATACAGCATGCACCCTTTGCCTTAAAACGGTAAAATGAGATAATGCCTTTCCGTTGTATCCCAGCCAGGCCAACCTCCCAGAGTATGTGCGGAGGGTAACGTCACGTAATGTAGAAAGTATTTCTGTATAGGTAGGGTACCCACCCGCATATGAAATTACTCTGTAGTTCCTGGGTCCCGTCGCCTCCGATATTCTTCTGCTTGATTTCTTTTCTGCCGTTTGCTAGTTTTCTTCAACCCCGGTTACGTTTATGCTTTTTCCTTTTGCTCTAGAAGACGGGTTTTTTTGCTCTTAACAAAGGCCAATCAATGCGATGGACTGGTCAATCTCGGCCGGACAGAGACAAACCCATCGTTCTTGACGATTGGGTTACAGTTCGCAGAGAGAAATGTAGTGGAATGGAAAGAGATCGCAGCAGTATATAGTCAGTTTATTCAGGTTACGCTATGGCATTTTTTTTTTTTGTTACGTAACTTGAGTACGGTGTTAGGTTTCTCTTTCTTTAGAAATTAGGGATGCTTCGGACAATTGGGGTTTTAGAAGAAGGGATACAAAGGAGTAGCATGCGTCGGGTGGTAGAGCTAATGTTAGCTGGTTGGGCTGGCCTGCCGGCTAGAATGGTTAATTGGTAGACCAGGTTTTTTTTTTTTCCCTTACTTCATTCCCTTACGTCTTTTGGGGCGACACAGGGTTTTGTATGAAGAGAATTGCATTTGCTATTAGTTAAGGCTTCTCCGAACCTGTATAAAAGTGGAGAGTTCGCAAGGTATGTAATTATATATACAGGATATATTTCCCTGCAATGCTAGTATTTAATGATGATAGACAGTTGGTTTTACAGGACAAAGATTCGTTTGGTCCATTGATTGAATAGCACACACTTCTGACTACGTAGTTGTTTAACAAGATCGAAGTGAATCTGACTCTAAGAATACAGTATACGAAACAATGCTTTCCAGAACAATCCGTACACAAGGTTCCTTCCTAAGAAGATCACAACTGACCATTACAAGATCATTTTCGGTTACATTCAACATGCAGAATGCACAAAAGAGATCACCCACAGGAATTGTTTTGATGAACATGGGTGGCCCCTCTAAAGTTGAGGAAACATATGATTTTTTGTATCAATTATTTGCCGATAATGACCTAATTCCCATTAGTGCTAAGTATCAGAAGACAATTGCTAAATATATTGCTAAGTTTCGTACCCCCAAGATAGAGAAGCAATATAGGGAAATTGGTGGGGGCTCCCCAATCCGGAAATGGTCTGAGTATCAAGCCACTGAGGTCTGTAAAATCTTAGATAAAACCTGTCCAGAAACGGCGCCTCATAAGCCTTACGTGGCGTTTCGTTACGCAAAGCCGCTAACCGCAGAAACTTATAAACAAATGCTAAAAGATGGCGTGAAGAAGGCAGTGGCCTTTTCTCAATATCCTCATTTCTCTTATTCCACTACCGGGTCATCCATCAACGAATTGTGGAGACAGATTAAGGCATTGGACTCCGAGAGATCTATATCTTGGTCGGTTATTGATCGTTGGCCTACAAATGAAGGTCTAATCAAGGCCTTCTCCGAAAATATCACCAAAAAACTACAAGAGTTTCCGCAACCTGTCAGAGACAAGGTTGTTTTATTGTTTTCCGCACATTCTCTACCCATGGATGTTGTTAACACCGGTGATGCCTACCCAGCTGAGGTAGCTGCGACGGTTTACAACATCATGCAAAAATTAAAGTTTAAAAACCCTTATAGGTTGGTTTGGCAATCCCAAGTTGGACCAAAACCATGGTTGGGAGCGCAGACAGCTGAAATTGCGGAATTTTTAGGCCCCAAAGTTGATGGCCTAATGTTTATTCCTATCGCCTTTACCTCTGATCATATTGAAACATTGCATGAAATTGACTTAGGCGTCATTGGGGAATCGGAATATAAGGATAAATTTAAGAGATGCGAATCTTTAAATGGCAACCAGACCTTTATTGAAGGCATGGCAGATCTCGTCAAAAGCCACTTACAGAGTAACCAACTCTATTCTAATCAACTACCTCTTGATTTTGCACTTGGCAAGTCCAATGATCCTGTAAAGGACCTTTCATTGGTATTTGGCAATCACGAATCTACTTGAAGATAAGACAGGGGAGAGGGATTCTTCTTCTTACCGACTCAACGCACCACACACCCTCTTTCCCAATTCTAATCATTCGATCTATGTAAACTTATCGTAACTAAACTAACTAACTACCAAGATTCTTCTTTACTTTACAGAGGTGTATTTCTTTAGTTGTCACATACAAAAACGCACACACACACACACATTTTGTCCTGTCCAATGACAAAATGTGACAGGAAACCCGAACGTCGGAGGTTTTCGGGTGGCAGTCATGTCGCCCAGTACTACTCGTAGCAGTAGTACTACTACCATTACACAAGGGCCAAACGTCAATGCTTTGCCGCGGGAGAGCCTGACCTATTGCATCTCCCGCCGTGCACAGGGTGATTTAAAT

LHZ1082: P_FBA1_- *HEMA_Sty_* -T_FBA1_,P_HXT4_- *HEML_Eco_* -T_HXT4_ ,P_OM45_- *GLTX_Km_* -T_OM45_,P_ADH1_- *HEM1_Km_* -T_ADH1_

GCGGCCGCGCGCTCCCGATTCCGGAAGTGCTTGACATTGGGGAATTCAGAGCTCACAAGGGCCAAACGTCAATGCTTTGACGTCTCCCGGAGGTATCCCGTTCCAGTGCGTGAGGGGCGTGGAGCGACAAACACACTCCCACATACATCTTTCCCGGAGGCAGAAACAAAATAAGAACAACAACAACACACCAACACGCCAACCATAGTAACCCACACGCTTAAACAACCAGTATCAGACCGGTACCCAACTTCTACGGCCGGCGGCGGCTGGCAGCGGCCTCGAGCATCCTCTATTCTCTCACCTCTTCTCTCGCTGTTGTACAACTTACAGGAATTCCCCTCTCTCCTCGAACTGGAAGCCGTGTTTCACGTGAGTGGTGGTACGATACCCGGTGGGAACCCTGTGTTGTGATATTTCGCCTTCTGGTTTTTGTTTTTGTCTCTCCCCTCTGGCTTCCAATACCGGAAAATTTGAAATTTTGGAAAAACAGGAAATCACAAAAAAACAGGAGAACCTGGAAAACGTAACGAAACCAAATAGTGGAACCCAACTGAATCCGCCGTAGACCAGATCAACCCACATGATTAAACGTCGCCCTCGACTTCCAGTGGTACTTCCAGTACCAATTCCAGTACCACTTTGAACAACTCTCCATAACACTTCCAATTCCACTTACAGTGTATGTGTGTGTATGTGTGTGCGTGTGTGGACAGGTTTCTTGCGCTTCTTGTACTTCTGGTGATGATAGATGGTGGATTCTCGGTACAGGAATTGGCTCAGGCTTTTTTTTTTTTCACATCGATTTCAGTTTGGGCTTTCCCTATGCATTCATTATATTGTGATTGCATTGAGCTTTGGAAATTTTTCATACTCTTCGAAATCATATATAATAAGTTACAATGTGAGTTATTCTCTCCTTCCTGGTTGTTGGTGTAAGCATCATTTAACTAGTCCCTTTTTTTTAAAGTTATTTAATATATATTTTTATTTTGTCCCAAACACCAAGAAGTAATCTTTACTTTTAGCACACAAATAACCAATAAATTCTAAAATGACCAAGAAGCTTTTAGCGCTCGGTATTAACCATAAAACGGCACCTGTATCGCTGCGAGAACGCGTAACGTTTTCGCCGGACACGCTTGATCAGGCGCTGGACAGCCTGCTTGCGCAGCCAATGGTGCAGGGCGGGGTCGTGCTGTCAACCTGTAACCGTACAGAGCTGTATCTGAGCGTGGAAGAGCAGGATAACCTGCAAGAAGCGCTGATCCGCTGGTTATGCGATTACCATAACCTGAACGAGGACGATCTGCGCAACAGTCTGTACTGGCATCAGGACAATGACGCCGTCAGCCACCTGATGCGCGTCGCCAGCGGTCTGGATTCACTGGTGCTGGGCGAACCGCAAATCCTCGGTCAGGTGAAAAAAGCGTTTGCGGATTCGCAAAAAGGCCACCTTAACGCCAGCGCGCTGGAGCGAATGTTTCAGAAGTCTTTTTCCGTCGCTAAGCGAGTGCGGACTGAAACCGATATCGGCGCTAGCGCCGTCTCCGTCGCGTTTGCCGCCTGTACGCTCGCCCGCCAAATCTTTGAATCGCTCTCGACGGTCACCGTACTGTTAGTTGGCGCGGGCGAAACCATTGAACTGGTGGCGCGTCACCTGCGCGAGCATAAAGTACAAAAGATGATTATCGCCAACCGAACCCGCGAGCGCGCGCAAGCCCTGGCGGATGAGGTAGGCGCTGAGGTTATCTCGCTCAGCGATATCGACGCCCGTTTGCAGGATGCCGATATTATTATCAGTTCGACCGCCAGCCCGCTGCCGATTATCGGTAAAGGCATGGTGGAGCGCGCATTAAAAAGCCGTCGCAACCAGCCGATGCTGCTGGTGGATATTGCCGTACCGCGCGACGTTGAACCGGAAGTCGGCAAACTGGCGAACGCTTATCTTTATAGCGTCGATGATTTACAGAGCATCATTTCGCATAATCTGGCGCAGCGTCAGGCTGCGGCAGTAGAAGCGGAAACGATTGTTGAGCAGGAAGCCAGCGAGTTTATGGCCTGGCTACGCGCCCAGGGGGCCAGCGAGACCATTCGGGAATACCGTAGTCAGTCGGAGCAGATTCGTGACGAACTGACTACCAAAGCGCTGTCGGCCCTTCAACAGGGCGGTGATGCGCAAGCCATCTTGCAGGATCTGGCATGGAAACTGACCAACCGCCTGATTCATGCGCCAACGAAATCACTTCAACAGGCTGCCCGTGACGGGGATGACGAACGCCTGAATATTCTGCGCGACAGCCTCGGGCTGGAGTAGGTGTTTCCTCCTTTGGAATGACGCGCTTACTTTACGAAAGTATTAATTTATATATAACAAATTTTATATACTAGAAACGCATGAAATGAATTATGTGAATGGTCTCTTTATTATACCGAAATTGGGCCGCTACCAGCTGTTGCTCCCCTTGCCTATTAATTTATTGGTTTGGTTTGGAAACGGTTGGTGAATTGATTGATTTGTTCAAAACTTTCCCAAAGTGACAGTCACGTGCCGCATATCAGTCGTGGAGAAGTTCTTATAAGCGTCCAACCAGCCAAATAGTCAATTTTAAGTTTGTATTTTTCTGGGGAAAATTCGGATGGGGTGATGAATTCGAGGTTTCTCCACATTGTGTTGTTGTTTTGGTGCCGAAGATGGTAATTACAGCAAAATTAAAAAAAAAAAATGCATTAGATAAAATTTATTATTTTTGTTTGAGCTTTTTTAAGGCACAGATTCTGCCGCACAGGGCGCACACAGGGCGCGCCCGCGCTTACGGTAATAGCCCTGGAAAAGTCCAGGAATAGCCACGGATGGAGAAAAAGAAACAACGGAAAATCTTTGGGGGTGGTTTTAGTAGGCGGTGCACGGGTGGGATCATGCGTTTTTGTCGAGCGATGCCGGAAGAATCTGGGGAAAGCAGAGTTCCCGGCGTGAGATATGGAGTGGAAGCGCGCGTAGACGCGGGCGCAGTGAAGAAAGAGGGAGAGCAAGTGCTGCAGTAAAATAGGAAGTAGCAGGTAACAAGATCTGAGCTGGATTGGTTAGGGTAGTTGTTCCGATACGGGGGTGTCTGCGGGCGAAAAAAAAAACTGGGACCATTACATTGTCCAACAATAGTCACATCACACTATGAAATTTTTTTAACTAATTAGTGTATTGTTCCGGAAATAGTGTGTATGTGTGAGAGTATTTTCGCTTGGCTTACTTGGCTACTTGGCACCGGCTGCTAGATTCTGGTGCCAAGGTGGTGCCAAGGTGTCCAAGAGAAGAAGCGTTAGCAGACGCTAAAGGTGAACGGGTTTTCGCCATGAATTTTCTGATATAAAAGGAAGTGGTTTGGAACTGAAATTTGAAATTGGAATCGTATCTCAATCTATCTCTCTCTGTTATATTTATTATTATTATCTTTTGAATGCTCAGACCCCATCAAACAATAAGAATCACACACACTTAATATTATTATTACTATTAATTTTTTGCGACCACTATTAAAAGTGTCATTATAGTGAAACTGTTATAGGAATAAGAAGACTATAATATTTCCAAACAAAAAAACTCAAAAAATTGATTAAAAATGAGTAAGTCTGAAAATCTTTACAGCGCAGCGCGCGAGCTGATCCCTGGCGGTGTGAACTCCCCTGTTCGCGCCTTTACTGGCGTGGGCGGCACTCCACTGTTTATCGAAAAAGCGGACGGCGCTTATCTGTACGATGTTGATGGCAAAGCCTATATCGATTATGTCGGTTCCTGGGGGCCGATGGTGCTGGGCCATAACCATCCGGCAATCCGCAATGCCGTGATTGAAGCCGCCGAGCGTGGTTTAAGCTTTGGTGCACCAACCGAAATGGAAGTGAAAATGGCGCAACTGGTGACCGAACTGGTCCCGACCATGGATATGGTGCGCATGGTGAACTCCGGCACTGAAGCGACCATGAGCGCCATCCGCCTGGCCCGTGGTTTTACCGGTCGCGACAAAATTATTAAATTTGAAGGGTGTTACCATGGTCACGCTGACTGCCTGCTGGTGAAAGCCGGTTCTGGCGCACTCACGTTAGGCCAGCCAAACTCGCCGGGCGTTCCGGCAGATTTCGCCAAATATACCTTAACCTGTACTTATAATGATCTGGCTTCTGTACGCGCCGCATTTGAGCAATACCCGCAAGAGATTGCCTGTATTATCGTCGAGCCGGTGGCAGGCAATATGAACTGTGTTCCGCCGCTGCCAGAGTTCCTGCCAGGTCTGCGCGCGCTGTGCGACGAATTTGGCGCGTTGCTGATCATCGATGAAGTGATGACCGGTTTCCGCGTAGCGCTAGCTGGCGCACAGGATTATTACGGCGTAGTGCCAGATTTAACCTGCCTCGGCAAAATCATCGGCGGTGGAATGCCGGTAGGCGCATTCGGTGGTCGTCGTGATGTAATGGATGCGCTGGCCCCGACGGGTCCGGTCTATCAGGCGGGTACGCTTTCCGGTAACCCGATTGCGATGGCAGCGGGTTTCGCCTGTCTGAATGAAGTCGCGCAGCCGGGCGTTCACGAAACGCTGGATGAGCTGACAACACGTCTGGCAGAAGGTCTGCTGGAAGCGGCAGAAGAAGCCGGAATTCCGCTGGTCGTTAACCACGTTGGCGGCATGTTCGGTATTTTCTTTACCGACGCCGAGTCCGTGACGTGCTATCAGGATGTGATGGCCTGTGACGTGGAACGCTTTAAGCGTTTCTTCCATATGATGCTGGACGAAGGTGTTTACCTGGCACCGTCAGCGTTTGAAGCGGGCTTTATGTCCGTGGCGCACAGCATGGAAGATATCAATAACACCATCGATGCTGCACGTCGGGTGTTTGCGAAGTTGTGATTTTTCCAACATGCTTAATTCTGTTCTAACTCGTAACTTGTTATTGACTTTTTTCTCTTAACCAAATTAACACATTCATTTCAGAAGATATGCCTTACTGGGATGCTTCATGTCAGTGCTCCTTAGCTTTCTTTTGCTGGAACACGAACGAATAATAATGATAATATCTTTTTATCGTCCTAATATTAATGCTAATTGCAATAAAACTAATGAATGATAATGAACGACTTTTATAAACTGAACATACATTTTATAATATAGACTCTAAAATATATTTCAGTTACTTTCAAGCCACACGAATTTTAGTTTGTTTAGAGATGGACCATGGACCATGGACGGTTAATGGTTTATATACAGCATGCACCCTTTGCCTTAAAACGGTAAAATGAGATAATGCCTTTCCGTTGTATCCCAGCCAGGCCAACCTCCCAGAGTATGTGCGGAGGGTAACGTCACGTAATGTAGAAAGTATTTCTGTATAGGTAGGGTACCCACCCGCATATGAAATTACTCTGTAGTTCCTGGGTCCCGTCGCCTCCGATATTCTTCTGCTTGATTTCTTTTCTGCCGTTTGCTAGTTTTCTTCAACCCCGGTTACGTTTATGCTTTTTCCTTTTGCTCTAGAAGACGGGTTTTTTTGCTCTTAACAAAGGCCAATCAATGCGATGGACTGGTCAATCTCGGCCGGACAGAGACAAACCCATCGTTCTTGACGATTGGGTTACAGTTCGCAGAGAGAAATGTAGTGGAATGGAAAGAGATCGCAGCAGTATATAGTCAGTTTATTCAGGTTACGCTATGGCATTTTTTTTTTTTGTTACGTAACTTGAGTACGGTGTTAGGTTTCTCTTTCTTTAGAAATTAGGGATGCTTCGGACAATTGGGGTTTTAGAAGAAGGGATACAAAGGAGTAGCATGCGTCGGGTGGTAGAGCTAATGTTAGCTGGTTGGGCTGGCCTGCCGGCTAGAATGGTTAATTGGTAGACCAGGTTTTTTTTTTTTCCCTTACTTCATTCCCTTACGTCTTTTGGGGCGACACAGGGTTTTGTATGAAGAGAATTGCATTTGCTATTAGTTAAGGCTTCTCCGAACCTGTATAAAAGTGGAGAGTTCGCAAGGTATGTAATTATATATACAGGATATATTTCCCTGCAATGCTAGTATTTAATGATGATAGACAGTTGGTTTTACAGGACAAAGATTCGTTTGGTCCATTGATTGAATAGCACACACTTCTGACTACGTAGTTGTTTAACAAGATCGAAGTGAATCTGACTCTAAGAATACAGTATACGAAACAATGTCTGTTTCGCTAGTTATTGGTGCTAAGGCACCAGTTGTCGCTTATCCAGAGTTGATTGCAGCTCGTTTGGTTAATGCTTCTCATCAAAACGCCATTGATATCTCTTTTGTTGAGGATAAGAAAGCCCCAGCTGCCTCTTTCCAAGGCAAAACTGAAAATGTTTTAGCCGAGATTGCTGCTGCATATCCAGAAGTGTTGAAGAATGTGGCTGGTTTCGAAGAATGGGTCGAATTTGGTGCCAACCAGCTTGTAATTAAGAACTTCCAACAACTTGCCTCATCACTTGAAAAGTTGGACGCCCACTTAAATTTGAGAACCTACATTTTGAACACTGTTGAACTAACTTTGGCTGATATTGCTGTCTGGGGTTACTTGAGATCAAACGGTATGGTGGGCTCTATCATCAAGAATAAAGTCTACATCAATGTTTCTCGTTGGTACTCAACCTTGGAATCCATCCCAGAGTTTGGCCAAGCTCATGAATTTTTGACCAAATCTTTGCAAGAAATGAAGAAAGCTGCTAATGTAAACAAGAAGAAAGAAACACACAAGGCCAACTTTGAAATCGATTTACCTGATGCCAAGATTGGCGAAGTCGTTACGCGTTTCCCACCTGAACCATCTGGATACCTACACATCGGACATGCCAAGGCTGCTTTGTTGAATCAATACTTCGCTCAAGCTTACAAAGGTAAATTGATTATTAGATTTGATGACACAAATCCTTCGAAAGAAAAGGAAGAATTCCAGGACTCTATTTTAGAGGATTTGGAATTATTGGGCATCAAGGGTGACAGAATTACTTACTCTTCTGACTACTTCCAAGAAATGTATGATTACTGTGTTCAAATGATCAAGGATGGTAAGGCATATTGTGATGATACTCCAACCGAAAGAATGAGAGAGGAACGTTCTGAAGGTATTCCATCTTGCAGAAGAGAAAGATCAGTTGAAGAAAACTTGAAGATTTTCACGGAAGAAATGAAGAACGGAACCGAAGAAGGCTTGAAGAACTGTGTTCGTGCTAAGATCGACTATCAAGCTTTAAACAAGGCTCTAAGAGATCCAGTTATTTACAGATGCAACTTGACTCCTCACCATAGAACTGGAACTGCTTGGAAGATTTATCCAACTTATGATTTCTGTGTTCCTATTGTCGACTCTTTGGAAGGTGTTACCCATGCATTGCGTACAATTGAGTACAGAGACCGTAACCCACAATACGAGTGGATGCTAAACGCTCTTAACCTAAGAAAGGTCCATATCTGGGATTTCGCTCGTGTTAACTTCGTCAGAACCTTGCTATCAAAGAGAAAGTTGCAATGGTTGGTTGACAAGGATATTGTTTCCAACTGGGACGATCCAAGATTCCCAACTGTAAGAGGTGTAAGAAGAAGAGGTATGACTATTGAGGGCCTAAGAAACTTTGTTTTGTCTCAAGGTCCATCTAGAAATGTTATCAATTTAGAATGGAACCTCATTTGGTCTTTTAACAAGAAGGTTATTGACCCAATCGCTCCAAGACACACTGCTATTGTTTCACCAGTTAAGTTGCACTTGGAAGGTAGCGAAGTTCCTCAAACTCCAAAAATTGAAATGAAATTGAAACATAAGAAGAATCCAGATGTTGGTGAAAAGAAGGTTATATATTACAAGGATATTCTCATCGACGAAGAAGACGCTAAGTTGCTTTCTGAAGGGGAAGAAGTTACTTTGATGGACTGGGGTAATGCTATTATTACAAAGAAAAATGAAGATGGATCTTTGGTTGCAAAGCTACACTTGGAAGGTGATTTCAAAAAGACCAAATTTAAGTTAACCTGGTTAGCCGATACCGATGATAAGGTCGAAGCTGATCTTGTCGACTTTGACCACTTGATTTCCAAAGACAAGTTGGAAGAAGGTGACAACTTTGAGGATTTCTTGACTCCAAAGACCGAATTTCACACCAGGGCTATTGCCGACTTGAATGTCAAGGATATGAAGGTTGGTGATATTATTCAATTTGAAAGAAAGGGTTACTACAGATTGGATTCCTTACCAAAGGATGGAAAGCCTTACGTATTCTTCACCATTCCAGATGGTAAGTCTGTTAACAAATATGGTGCTAAGAAATAAAGATAAGACAGGGGAGAGGGATTCTTCTTCTTACCGACTCAACGCACCACACACCCTCTTTCCCAATTCTAATCATTCGATCTATGTAAACTTATCGTAACTAAACTAACTAACTACCAAGATTCTTCTTTACTTTACAGAGGTGTATTTCTTTAGTTGTCACATACAAAAACGCACACACACACACACATTTTGTCCTGTCCAATGACAAAATGTGACAGGAAACCCGAACGTCGGAGGTTTTCGGGTGGCAGTCATGTCGCCCAGTACTACTCGTAGCAGTAGTACTACTACCATTACGaagaaatgatggtaaatgaaataggaaatcaaggagcatgaaggcaaaagacaaatataagggtcgaacgaaaaataaagtgaaaagtgttgatatgatgtatttggctttgcggcgccgaaaaaacgagtttacgcaattgcacaatcatgctgactctgtggcggacccgcgctcttgccggcccggcgataacgctgggcgtgaggctgtgcccggcggagttttttgcgcctgcattttccaaggtttaccctgcgctaaggggcgagattggagaagcaataagaatgccggttggggttgcgatgatgacgaccacgacaactggtgtcattatttaagttgccgaaagaacctgagtgcatttgcaacatgagtatactagaagaatgagccaagacttgcgagacgcgagtttgccggtggtgcgaacaatagagcgaccatgaccttgaaggtgagacgcgcataaccgctagagtactttgaagaggaaacagcaatagggttgctaccagtataaatagacaggtacatacaacactggaaatggttgtctgtttgagtacgctttcaattcatttgggtgtgcactttattatgttacaatatggaagggaactttacacttctcctatgcacatatattaattaaagtccaatgctagtagagaaggggggtaacacccctccgcgctcttttccgatttttttctaaaccgtggaatatttcggatatccttttgttgtttccgggtgtacaatatggacttcctcttttctggcaaccaaacccatacatcgggattcctataataccttcgttggtctccctaacatgtaggtggcggaggggagatatacaatagaacagataccagacaagacataatgggctaaacaagactacaccaattacactgcctcattgatggtggtacataacgaactaatactgtagccctagacttgatagccatcatcatatcgaagtttcactaccctttttccatttgccatctattgaagtaataataggcgcatgcaacttcttttctttttttttcttttctctctcccccgttgttgtctcaccatatccgcaatgacaaaaaaaatgatggaagacactaaaggaaaaaattaacgacaaagacagcaccaacagatgtcgttgttccagagctgatgaggggtatctcgaagcacacgaaactttttccttccttcattcacgcacactactctctaatgagcaacggtatacggccttccttccagttacttgaatttgaaataaaaaaaagtttgctgtcttgctatcaagtataaatagacctgcaattattaatcttttgtttcctcgtcattgttctcgttccctttcttccttgtttctttttctgcacaatatttcaagctataccaagcatacaatcaactATGGAGTCTGTTGTTCGTCAATCTGCAAGGGTTTGTCCCTTCATGAAGTCTGCTACAGGGTCTGTGCAGAATGTGAAGGCTTTGAAAAACGCCAACTTGCCAGCTATTGCGCAAAAGTGTCCGTTTATGGGTCGTGCTATGGAGCAGCGTAGAGCATATGCGTCAGCATCCGGGTCAGGAGCTGGGTCAGGAGCTGGTGCAGCAGCAGCGGCTTCTCCAGCAGCCGTTGAAGCGAGGAACGCAAGTACTGCTTCTGCAGATGCTACTGTTTTGGACCATGCTACCAATGAAGCTTCCTTTGACTACCAAGGATTATTTGAGTCTGAGTTGGCCAGAAAGAGAATGGACAAGTCGTATCGTTACTTCAACAACATCAACCGTTTGGCAAAGGAATTCCCAATGGCCCACAGACAACAAGAGGACGATAAGGTCACTGTGTGGTGCTCCAACGATTACTTGGCTCTTTCCAAGAACCAAGAAGTCATCGATGTGATGAAGAAAACTTTGGACAAGTACGGTGCAGGTGCTGGTGGGACCAGAAATATCGCAGGTCACAACAAGCACGCGTTGCAGCTAGAAGCAGAAATCGCAGCCTTGCACAAAAAAGAGGGTGCGTTGGTGTTCTCCTCTTGTTTTGTCGCTAACGATGCCGTCATCTCGTTGCTCGGCCAGAAGATTAAGGATCTAGTCATCTTTTCCGATGAGCTGAACCATGCCTCCATGATCGTGGGTATTAAACACGCCTACACCAAGAAGCATATCTTCAAGCACAATAACTTGGAAGAATTGGAAAAATTGCTAGCCATGTACCCAAAGTCCACTCCGAAATTAATCGCCTTTGAATCTGTTTACTCTATGTCTGGGTCCGTCTCTGATATCAATAAAATTTGCGACTTGGCAGAAAAATACGGTGCCTTGACTTTCTTGGATGAAGTTCACGCAGTTGGTTTGTACGGTCCACATGGTGCCGGTGTTGCAGAGCATTGTAACTTTGAAGCTCACCGCAAAGCTGGTATTGCATCCCCAGAATTCCGCACTGTCATGGACCGTGTCGACATGATCACCGGTACCTTGGGTAAATCTTTCGGTACTGTCGGTGGTTACGTTGCTGCTTCCTTAAAATTAATCGATTGGCTCAGATCTTACGCACCAGGATTCATCTTTACCACATCTCTACCACCATCCGTTATGGCGGGTGCCGCAGAGGCTATCAGATACCAACGTTCTCACTTGGACCTAAGACAAGACCAACAAAAACATACAACTTACGTCAAGGAGGGATTATCCGATCTGGGTATTCCAGTGATGCCAAACCCATCCCACATTGTCCCAGTTTTGGTTGGTAACCCTCATTTGGCTAAAGAGGCTTCGGATATCTTAATGCATAAACATCGTATCTACGTTCAAGCAATCAACTTCCCAACGGTTTCCAGAGGTACCGAACGTTTAAGAATTACTCCAACTCCTGGTCATACTAATGATTTGTCTGACATCTTGTTGGATGCTATGGAAGACGTTTGGTCAACCCTACAATTACCAAGAGTTCGTGACTGGGAAGCCCAGGGCGGCTTATTGGGTGTTGGTGATCCAAACCACATCCCTGAACCAAACTTGTGGACTGAAGAACAATTATCTTTGTCTAACGATGACTTGAACCCAAATGTCAAACATCCAATAATAGAACAATTGGAAGTTTCTTCTGGTATTAGATATTGAgcgaatttcttatgatttatgatttttattattaaataagttataaaaaaaataagtgtatacaaattttaaagtgactcttaggttttaaaacgaaaattcttattcttgagtaactctttcctgtaggtcaggttgctttctcaggtatagtatgaggtcgctcttattgaccacacctctaccggcaTGAATAATGAATGGCCTTGTATTCGCCGCGGGAGAGCCTGACCTATTGCATCTCCCGCCGTGCACAGGGTGATTTAAAT

LHZ1083: P_PDC1_- *HEM2_Km_*-T_PDC1_,P_PGK1_- *HEM3_Sc_* -T_PGK1_

GCGGCCGCGCGCTCCCGATTCCGGAAGTGCTTGACATTGGGGAATTCAGAGCTCTGAATAATGAATGGCCTTGTATTCGTTTTTTTCCGAGAGAAAATTAACAAGAGCGAAAAAAAAAACGGGCTTCGGTGAAAATCGGGTGAATATGCAACTAGCGGGACGAATGCTCTGGAAATGCATATCCTATGCAACTAGCGGGATGAACAAATCTCACCCCAGAATTCGCAGGAAAAAACAGGAAAAAAAAAAAGAAGGCCACCACGGCCACAAAGACCACAAAGGCCACAAAAAGAACAAAAAAACAACCGTCCCAGCTTCCAGTGTTTGGAATACTGGAACACAGGAAGCCGCATAAGAGTGGGCGTTGCACAGGAAGCCAGGCCCAGAAGCCCCAGAGTTACTTTTTTTTTTTTGTTTTTTCCTTCTGTTCGCTGTGCCCGCATCAGATGATGCGCCTTTATTTACGATGCCAATGCGAATAGCACCAGTGAGAGCACCAGTAAAAGCATACGCATACACATACACACATAGAGCAAGCAAGCAGGCTAGCAACCAGGAAAGGCTGCCAGTGACTGCTACTGGGTGTCTAAGAACCGTAGGGCGGATTATTGTTGCGGTGGTTGGTTGCGGGTGGTTATGCGATGGTACGGTGCAGAATCGTACGGTGTTGGTTATGGAATTAGTATGGGTATGTGATATGTGGTAATATGTGATATTGGGTTATTGTGATTTGGAATACTGAATATCGAATATGGGATATGGAATATGGCCATGGCATGGTATGGTATGGGATGGGAGTATTCTATTTTATTTTATTTTATTCTGGTTCCTGCGTTTAGGGTAGGGTAGGAAGAAGGTGAGTGCTTTTGTATATAAGTGGAGTGTCTGGATCAGTTTTGTGGATTGTGAATGTTAGTTTCCCCTTTAATGTATATTTGTATTATTTGCTTTTGAGTACTCAATAACCAAGCACAACTACTAGTTTTAAAGGATCCATCCTCTTAAACAGTACAAATCGCAAAGAAAAGCTCCACACCCAAACCAAATAATTGCAATGGTTCACGTTGCAAAATTTTTAGACGAACGTCCTACTGAAATTCCTTCCATCCTTGCTGGTGGATACAACCACCCTCTATTGAGAGAATGGCAGAATGAAAGACAGTTGACTAAGAGCATGTTCATTTTCCCGCTATTCATTAGTGACCAGGACGAGGAAGAGACCCTGATACCATCGCTACCAAACATCAAGAGATTTGGTATCAACAAATTGAAGGATTATGTTGCTGGTTTGGTTGCCAAAGGTTTACGTTCGGTGATCTTGTTTGGTGTTCCATTGAAGCCTGGTGCTAAGGACGAGGTTGCAACCGCAGCTGACGATCCAGATGGGCCTGTCATCAAGGCTATCAAGTTGTTGCGTAAGGAATTCCCAGATTTGTACATTATCTGCGATGTGTGTTTGTGTGAATACACTAGTCATGGTCACTGTGGGATCTTGTATGAAGATGGTACTATCAACAGAGAAAAATCTGTGCAAAGAATTGCTGCTGTTGCTGTGAACTATGCGATAGCTGGTGCGCACTGTGTTGCCCCAAGTGATATGATTGATGGTAGAATCAGAGAGATCAAAATGGGATTGATCGAACACGGATTAGCCCACAAGACGTTTGTTATGAGTTACTCTGCCAAGTTTAGTGGTAATTTGTACGGTCCATTCAGAGATGCAGCATGCTCGCAACCAGGTCAAGGTGATCGTAAGTGCTACCAGTTGCCTCCAGGTGGTCGTGGTTTAGCCAGACGTGCATTGAAAAGAGATTTGGCTGAGGGTTCCGATGGTATTATCGTCAAGCCTTCGACCTTTTACTTGGACGTTATGTCCGATGCTGCTGAAATTTGTCGCGACATTCCAGTTTGCGCATACCACGTCAGTGGTGAATACGCAATGTTGCATGCTGCTGCTGAAAAGGGTGTTGTTGACTTGAAGAGCATTGCTTTTGAATCCCACTACGGTTTCTTGAGAGCTGGTGCTCGTTTAATCATCAGTTACCTCACCCCTGAGTTTTTGGAATGGTTAGATGAAGTGAACTAAAGAGGGAGAGGATAAAGAGATAAATTACGATTTTGGATTTTAATGATTTTATAAACAACAACAACCAACCAGCCTTTTACTTTATTTGGCATATACACAAGCTTACTCCATTTCATTGATTATCTATGTGTATATATATAAGTGATGTATAACAATTATTATTATACATAGATAATATTTTTATGATATGTTTTTTCTGAGTTTTGATATTATTTATTACAAGTTACAAGTTACAAGTTACAAGTTACCAGGAAGAATTAAATAAAGGTAAATTGGGGGAATTATAAGCGTATGGGCATAGCCTTGTCCAAGGCCTTGGCCTTGGTCAATGCCCTTATTCTCTTGTTTCCATTGCCTGTCGATATACGACTTCAAGGCCATGTACCTGAGATCCTCATCTGGGGCCTGAAGATCCATTGGTGAGTGCCACTGGCTGCTGTGCCGTTGTGAAAAGGCCCTGTGTAGCAGAGAGGGAGGTGTGTATAGGTATATATATGTATATATATGTATATAAGTGTGGATTTAACACACTCTCTCTTCTTTCCCATGCCATGCCAGTCACGTGTTCCACACGTGACTGTACTTACCCTCACATTACCCTCACTCTTTCACATTACCCTCCCCATCCCATCTTCTTTGGTTTTCCCCCCCAGCTTCCTGTCTTCCACTATCCCTGCAACCACCACCACCACACAACAAACATCCCCCAGCAGCCCCCCAGCAGCCCGGAAAAATCCACGCCTCCCCCCGAACACACGAAGCCCGCCCCGGCTTCCACTCCAACACTGCCAACACTGGAACCCCGCACCACGTAACCACCCACTTTAGTGGCTGCCCGCCCCTCCTGCGCTTCCCTGGGGCTTCCCTTGCGACCACACTCCGGTGGCATGGCCCTCGCTTGCTACATGCTACCTTTTTTTTGCCCTTTTCTGGCCTTTTCTGGCTTTTCTGGCCTCACCACAGTGTACCACAGTGTAGTAGTAGTGTAGCATTGTCGAGTGGTATTGTCGAATGGTATTGTAGCCTGTTGTAGCCTGTTGTAGCCTGGTGTAATGTCTGGCATAGTCTGGTGTCTGGTGTCTGGTGTCTTTTGGAAACTGAACAGTGGAAAGTGGAAGTGAAAAATTGTATAAATATAGGTGTCCCATTCGTGGTTTGGTGGATGTGTCCTTGACATTGCAGGTTTCTCTGTTTCGCTGTGTTAAGACTTGTTAACACTTGATAAGTATTTCCCTAGTAATACCCCACCAACCATCGGTTGATACATTTTAATACACACTACCTATATAAAGATACAAAAATGGGCCCTGAAACTCTACATATTGGTGGGAGAAAATCGAAATTGGCGGTAATACAATCCAACCATGTTTTAAAACTGATCGAAGAAAAGTATCCGGACTACGACTGCAAGGTTTTCACTTTGCAAACTCTTGGTGACCAGATTCAATTCAAACCTTTGTACTCATTTGGCGGTAAAGCTTTATGGACAAAGGAGTTGGAAGACCATCTTTACCATGACGATCCCTCAAAGAAGCTTGACTTGATCGTTCATTCTCTGAAGGACATGCCCACTTTACTACCAGAGGGTTTCGAGCTGGGGGGTATCACTAAGCGGGTCGATCCAACAGATTGTCTTGTCATGCCCTTTTACTCTGCTTATAAGTCTCTGGATGACCTTCCAGACGGGGGGATTGTGGGAACCTCATCCGTGAGAAGATCTGCTCAGCTAAAAAGAAAATACCCACATTTGAAATTTGAAAGTGTCAGAGGAAATATACAAACTAGATTACAAAAACTAGACGACCCAAAATCTCCGTACCAATGCATCATCTTGGCGTCTGCTGGGTTGATGCGTATGGGGTTGGAAAACAGAATTACGCAGCGATTCCATTCGGATACAATGTACCATGCAGTTGGACAAGGCGCCCTGGGTATAGAAATTAGAAAGGGTGACACCAAGATGATGAAGATTCTTGACGAAATTTGCGATCTAAATGCAACTATATGTTGCCTTTCGGAGCGTGCTTTGATGAGAACTTTAGAGGGGGGTTGTTCCGTTCCTATTGGTGTGGAATCTAAATACAATGAAGAGACTAAAAAATTACTATTAAAGGCCATTGTAGTTGACGTTGAAGGCACAGAAGCAGTAGAAGACGAAATTGAAATGCTAATAGAAAATGTTAAAGAAGATTCCATGGCGTGTGGTAAGATACTAGCTGAAAGAATGATTGCCGATGGCGCAAAGAAAATTCTGGATGAAATTAATTTAGACAGAATCAAATGAGTGCATTGTTGAACTTTCTAATGAAACCACACGTGAATTAAAGGAAAGCGCGCAGCAGCAGCAGCAGCAGCTTTTCTTTCCTTCTTCAAATCATACCTTTTCTCTATTTCAACGTTCTTTTCATTTCACCATGAAATGAGTTGAATTGTTGCTTTGAGTTTACGACGATACATAAATATTAATTAAAATACGAAAAATACACATTTTATTTACGACACTTGCCTCTCCCCCTAGATGTTACGCTGCTCAGGTTCGAGCCGCTCGATCCCTAGCATATCGAGAACTTCGTACTCGTCCCCCATTTTCAATTTCCCAAAAAAACAAGCCGCGGGAGAGCCTGACCTATTGCATCTCCCGCCGTGCACAGGGTGATTTAAAT

LHZ1084: P_ENO2_- *HEM4_Km_* -T_ENO2_, P_INU1_- *HEM12_Sc_* -T_INU1_ , P_AFT1_- *HEM13_Km_* -T_AFT1_, P_TEF_- *HEM14_Sc_* -T_TEF_, P_OM45_- *HEM15_Sc_* -T_OM45_

GCGGCCGCGCGCTCCCGATTCCGGAAGTGCTTGACATTGGGGAATTCAGAGCTCATTTTCAATTTCCCAAAAAAACAAGAAGCCGGAATCCACCGGAATCCACTAATACGCAAAAAAGACGACGCCAGCTTCGCCGTTGGGCATTGGCACCATATTCCATCGTTCGTTTGACTTTGGTTTTGGTTTTAGGTTCTGGCTTCGTGTTTTCCCCTCACTGCCCTTGTCCTTGCCGTGCTCCCCCTCGTTCTCTGCGCAAAGTCTGGTGACCCTCCGGCTGCCACAAAATCCCGAGGCACTGAAAGGGTGGGGCCTCGGGGGTCTCGGGGCAGAAGCCTTGAGCTTCGTGTTTATTCACTCTGTACGGGCGGTTCTAGCGTTTCTGGGAATCCAAAGAGGAAGGGCCGTGTTTCGTGCAGGCATTTTAGTGGATGCCCGGGATGTAAGCTCTTCGGTTACAGAAAATCAGGTTGTGATTCCAGTATGAGGAATAGTGGATGCCAGGTTGTGCGAGCATCACCAATATCATGGGAAATGCGAAATGGGCATGAGCCCAACCAACACAACACAATACCATACGCCAAATGGTAGCCTGAAAAAAAAAAAAAAGGTCTTGCCAGACCACTAAACTCGAGGTAGAACAAGACTGAGAAAGAGTGTGTGATCCCTTTGGTGGTAGTAATTTTTTTTTTTTTTTTTCAAGTTTCCAGCATCCCAAACCGAAACCCAACATCATCATGATCCCATCCCATGCCTATGCCCATGCCACATTCATTCATTGCACAACACACAACGTAGTGGACGACAGCATAGCATCAGTTAACTAACGGCCACTTGTTTTTTTTTCTTCCATTTTTTCGTTTTGTTCTAGCAACAATGAGTTCTAATTTTTTTGTTATAAAAGGGACAGTTAAGGTATATGTAAGGTTTCTTGTTCCATTTGGGTTAGAGTTTTGTAGTATTAGTTTTGGTTCTTAGTTCTTTTGGCCTAAGAAACCAGTAAGAATTCTTCTGATTTTCTTTACAAACACATCAACAACAACAAACACAAACTACAATGTCCAGAATAATTTTTCTTAAGAACAAAACAACACCTACAGACCCGTACGAAACCACTTTTACAGATGGAGGGTTTGAACCTGTATTTGTTCCACTAATACGCCATTTCCATGTTCCAGACGAAGCGTTGACGCTATTCAGAAACAGGAGCTACCTAACGAAATTGAAATACATTATAGTTACTTCACAACGAACAGTCGAATGTCTAAATGAGTCAATACTTCCTAAAATGACTACGGAAGAGCAATCTCTTTTAAAGGAAAAGACTATATACACAGTTGGACCGGCCACTTCGGAGTTCTTGAGAAATTCTGGTTTTCAAAACATAAGAGGAGGAGTTGAAGTTGGAAATGGCGGATTACTTGCTGATCTGATTGTTAAATCGCATTCTGAGGAAGAGATTGATCACTTTTTGTTTTTAGTTGGAGAAATAAGAAGAGATATCATTCCCAAGAAACTTAAAGCTAATGGCTACAAGGTCAACGAAATTGTAACTTACAAAACCGAGAATCTGTTAGACAATATTGATCGTTTTATTTTGCACTATAAGAATGATGAGAATAACCAAGCGGTTATGAACAAGCCCTCCACATGGATTGTGTTCTTTAGTCCCCAGGGTACGGAAGATATAATTGAGTACCTAAAAGAGAACAAGGGCTATAAAATTGCTAGCATAGGTCCTACAACGGAAAAGTACCTCCTAGAAAAAGGTTTGAAACCAGACACTGTTAGTTTTAAGCCGTCTCCAATATTCTTACTGAATGCCATTAATAGTTACAAATAAATCTAGCTGACGCGACGCTTTTTTTCTCCAACTAAGGGAATCCAGGGAAACCCCCCTCGAGCCCCAGTTTTGGTTAGAAATTGTCCTTTTTTATACTATTGAATGACCTAACTAAATCTATATAAACCTTTTTAACGTCTAACGACTCTTTATTATTGAAGTGGGAAGTCTTGGGATGGGAAGTCTTGGGATATTTCAGGCCTTTGGCCAGGCCAAAGGCCAGGCCTTGGCCCAAGGTACCACCCCCTTATATATGCTCAGCCAATTGACTCAGCTGTCCCTCCACACTTTATTCCTGTAATCCGAAAAGGTAAACAGACACAAAAACGACAAGAGAAGCAAACACAAAAAAAAAAAAAAAAAAAAACAAAAAAAAAAAACACAAACACAAACACAAACACAAAAACGCTAAATTATGCACACAAGGGCCGGCGGGGCTGCCGGAAAAAAAAAGGGAAAAATACACAGACGAGCGCGCACAGATGGGGTTACCACTGCAAGTTACAAGTTGCAAGTTGCACGCTGGAATCAGAATTGGAATCAGAATTGGAATTGGAATTAGAATTAGAATTAAACTTGGGGTAGCCACGGGAACGGGATAACTCAGGAATCGCTCGCAGGCGTCTCCGTCTAGGCAATCCCAAGGTAAGCCTAGGCACTCCCACAGGGGAAAGAACGGTTGAAGGCAAAGTAGTGCTAACAATTGGTAACGAATGGTAACAAGTGTGTCCGTCTCCACCTGACATTTGCTAGAGCTGGGGATTCCACATTCTTGTGCTCTGAATTCTCAAACCGAAATGGGGCGTTGTTACCCCAGGTATCCGGTTGTAGTTGGCACTGGGGATGGAAAAAAATGATGTTGATGTTGAGTTAGTTGGGTTGAGTCAATTAGTGCGTGAAAGTATCACCACTTTTGTCATCCGGCGTTTCTGTGCGAATCACACACACACACACAGTTTATTGGAGCACTTGTTTCTGGCGTATTCGTAATTGTTCTGCGGTGCGGTTCTGTGTGCATTTTTCCTGGGGTGTCTGCCGCACCTACTCATCACCCACGCCGTGGGTTTGAGCCATGGCGGAGGTACGACTGACTGGCTGCCTGCCTGCCTGACTGACTGCCTGACTGCAGGAAAAGAGGGTTTCGAAGGAAAAACTTTTCCTGTGTTAATCCGGCCGTGCGCCGCTGCTCCAAAATCCACCTTCATGAGAAGGAGTTTGAAAAAACAAAAAAATTCACATATAAAAAGCGTATCTCGAGATCTCAAAGTCTCCCTTGAATCGTGTTTGCCAGTTGTAACTCATCCTTTATTCTTCTATTCTATCTCTCTCTTTCCTTCCCCTAATCAGCAATTAAATCCGGGGTAAGGAAGAATTACTACTGTGTGTAACGGTTATATTTCGTTTTTTATTTTTTTTTTCCATTGCCATAGAGAAAGAAAAAAAAAAAAAAGAGAGTTTGTGAAGATCTTCCATTCGAATCCCATAAGTGACACATTTAATTTTTTTTTTGTTAGATATGGGTAACTTTCCAGCTCCAAAAAACGATTTGATATTGAGAGCCGCAAAGGGTGAAAAAGTCGAGAGACCGCCATGCTGGATAATGCGCCAGGCAGGTCGTTACCTGCCGGAATATCACGAGGTGAAAAACAATCGTGATTTCTTTCAAACTTGCAGGGATGCGGAAATTGCTTCTGAGATTACTATCCAGCCGGTAAGACGCTATAGAGGCCTCATTGATGCTGCTATTATTTTTAGTGATATCTTAGTTATTCCGCAAGCCATGGGTATGAGGGTCGAGATGCTCGAAGGTAAAGGTCCACATTTCCCAGAACCTTTAAGAAATCCGGAAGACCTCCAAACGGTATTAGACTACAAGGTTGATGTTTTGAAAGAGTTAGATTGGGCTTTCAAGGCAATCACCATGACAAGGATCAAGTTGGATGGTGAGGTTCCCTTATTTGGCTTTTGCGGGGGACCTTGGACTCTAATGGTTTATATGACGGAAGGCGGTGGATCCCGTCTTTTCAGATTTGCCAAACAATGGATTAACATGTATCCAGAGCTTTCTCACAAATTATTACAAAAAATCACTGATGTGGCCGTGGAGTTTCTGAGTCAGCAAGTCGTGGCGGGTGCTCAAATACTACAAGTTTTTGAAAGTTGGGGTGGTGAGCTTTCGTCTGTAGATTTTGATGAGTTTTCCCTACCATATTTAAGACAAATTGCCGAAAGAGTGCCTAAAAGATTGCAAGAATTAGGTATCATGGAACAGATTCCTATGATCGTTTTTGCGAAAGGGTCGTGGTATGCTTTGGACAAGCTATGCTGTTCAGGATTTGACGTTGTTTCGTTGGACTGGTCCTGGGACCCAAGAGAAGCGGTAAAAATAAACAAGAACCGTGTCACCTTGCAGGGCAACCTGGATCCTGGCGTCATGTATGGTTCTAAAGAGGTAATAACAAAGAAAGTTAAACAGATGATTGAGGCTTTTGGAGGTGGGAAGTCCCGCTACATTGTTAATTTCGGTCACGGTACCCACCCTTTCATGGATCCAGACGTCATCAAGTTTTTCTTGGAGGAGTGCCACAGAATTGGTTCGAAGTAAGGCCGCAAGCTTTGATCTGATCTGCTTACTTTACTAACGACAAAAAAAAATCAAAAAAAAAAAAACAATCAGTCCTTCTCTTCTTACGATATGATATGATTAAATGATGCTATGAAATCATCTTCTTCTTAACTTTCTTAAATCTTACGCGTCACTTACTCTATATACCCGTTTAGCTTTGCCTGGTCACAGCGACATTTTATATAAGTGTACGTATTTTCTTTTTTTTTTTAAAAATTTCTATTCTAACCTTAGAAAAGTGCCCTTTAAACCAGCTGTCCTGGCACTATATCTTTATCATGTGCCGGTCGCTTTCCCTTTCCGTTTCCCTTTTCCTTTCAATTGGTGGCCTGGAATTCCGAACTCATTTTCGCATCTGAAACTAATTCTCGAAACCTTTAACATCAAACAATTGAAAAGATCATCATCACCAGAAATAAGAAAAAGATCAACACAACAGCTAATAACAGTACGAAAGAAAGATCGCTCGAGTGAAAAGGCAGCCAAGAAAGGTCATTCGATTTGGGTCTAGACTGATTATAGACATACCAATTGCACTCAGTAAGAAAATGAGTTTCAAATTTGACGATGACGGTGTGGTAAAAGAATTTCACGGCAACACCATCATATGCCATATTCCTCAACAAACCGAATTCTTCAACAAATTGTTGGACTTCTACCGTTTTGCGAAACGACTTTCCTTCTACGACAAGATCACCCTACTTCCTCCTTCAAGCTACCACGTTACGATCATGAATTGCTGCCACGAACACGATCGTTCTGAGGGCCACTGGCCCAAAGGAATCGATCCGGACACAAGCATGCTGCGGTGTACATCACATCTGACCAACATTCTAGGATCGGTGCCAGGTGAATGTACGTAATGGAGTTTGGATAGATTCGATACAGTTCATTACCAAGAATGGACAGCAGTCCCCCATATACGGGAATGCAACCGGAGGATCATTGAACCAGATTAGCATTGTCGCTCCATACAACCTTAAAGGGTTCTTTGGTAATTGTTCGAATTGGATGGACAGTCTTGGAGTGTTGTACGGGTAATTGGAGCCTGTTTGAAGCGTTTGTTTCCCTTTGGAGCGGTTTCCGTGTCATCAAGATGTCGGGGTCCGTCAAAAAAAATTTAGCGGGCACTCCGGGGTCCGTGGGGTCCGTGTGGTAGGGTGTGCCAGACCCGGTACTCTAAATCCATCCCAAAGAGCAAAGAAAGGGTTGCATGAATAATGGACCCTTGTTGCGAGCAGTTACTGTTGCAAGCAGTTACTGTTGATAGAGATGGTGTTCGCTAGTATACAGGTCAAATACTTAAGGTTATCCTTTGTGTGGGAGGATTTTAGATTACCGAGTCTTTTTTTAGTGGAGTGCAGTGGGATGAGAAAAGTTGGCCCGACCTCGGAGAATTTCTCGTAATATGTTGCTCAATCTATTAAAAAAAAACTCGGATACTTCGAAAGCACCCCGTGTGGTTATCCCATGCGCGTAAAATTCACCCTCTGCGAATCGAGGGCGCCCCTTCAGAAACGGGATAACCGGGTGTTTTAGCCCAGGGCAATATCCAATAGGGCTAACCTAGTCGCTTTCCGAGCCCAATTTGTTAGTCTTGGCGCACCCCGATTGCTTGACACGGAGTCCGTCAATACCTATAAAATTGGGATTTAGAGGTTAATTATTTTTTAGGAATCTGTAACCACACCCCTCTTCGGACCCCGCCTAGTATTTTCTTCTCATGCCCGAAATAAATAAAAGGACAATTCTCCCCCAGAATATTACTACTTGTTCATTGTTTTAAAATTCCAACCAGGCTTCGTTACTCCTAAAAAATTTTCGAACCACGTACACCTTCAGCAATGACAGACTCTGCTGTGCCAATGAGAAAGAAGATGGAAGAGTTGATTCGCCGTAAGCAAAAGGAAATCACTAAAGGGTTGGAGGAATTGGACACTGTGCAGTTCCGTGCAGACTCCTGGACCCGTGGTAACGATGGTGGTGGTGGTACCTCTATGGTTTTGCAAAACGGTTCTACTTTCGAAAAAGGTGGTGTCAATGTGAGTGTTGTTCACGGTACTTTGACTCCTCCAGCTATTAGAGCGATGAAAAACGATCACAAGAACTTGCACTTGCCAATTGACCCAGAAACTGGCGAGCCAGATGCTTCTGGTGTCAGATTTTTCGCTTGTGGTTTGTCGATGGTCATTCACCCAATCAACCCACACGCGCCAACAACGCATTTGAACTACAGATACTTCGAGACGATGCATGCGGACGGGACACCTCAGGCCTGGTGGTTTGGTGGTGGTGCCGATCTTACCCCATCGTATTTGTACGAGGAAGATGCAAAGTTGTTCCATCAATTGCACAAGGACGCTCTAGACAAGACAGACGTTACCCTATATCCAAAGTTCAAGAAATGGTGCGATGAGTACTTCTACATCAAGCACAGAGAAGAAACCAGAGGTATTGGTGGTATCTTTTTCGATGACTTGGACGACCGTGATCCAGACGTTTTGTTGAACATGGTCGAAAACTGTTTCGATGCGTTCTTGCCCTCTTACACGGAAATCATCAAGAGAAGAATGAACATGCCATACACCGAGGAGGAAAGACAATGGCAGCAGATTAGACGTGGTAGATACGTCGAATTCAACTTGGTCTTGGATAGAGGTACTCAATTTGGTTTGAGAACTCCTGGCTCTCGTGTTGAGAGTATCTTGATGTCCTTGCCTGTTACCGCCTCCTGGTTGTACGACCACCACCCTGAACCTGGCTCCAGAGAAGACAAATTGCTACAGGTCTTGAAAAACCCAATCGAATGGGTGTAATGGGTGCTTAAGATCTGTCTTTTTTATTTATAATCGGCCCTTTTATCACTTTATTTTTTAGTTTATTTATTACATCTTTCGTCTGTATACTAGTTTGGGTCAAAGAAGTCAAAAATCGCACAATTTGGGTGTAGAACGTAAACGTAGTTAACACCTTGCGCAGCCTCTGCATCAGCGTCTTCGAGATCTTGAACATCTTGTTCGTTGGGTGCAACTTTCATCAAGATCTTGTCATGTACCAATTTACTGAGAACCTTGTGCACCAACTTTCTTTCAGCCCAATATTCTTCCTCTGTATGAAGCTTGCCTTGTCCCCGCCGGGTCACCCGGCCAGCGACATGGAGGCCCAGAATACCCTCCTTGACAGTCTTGACGTGCGCAGCTCAGGGGCATGATGTGACTGTCGCCCGTACATTTAGCCCATACATCCCCATGTATAATCATTTGCATCCATACATTTTGATGGCCGCACGGCGCGAAGCAAAAATTACGGCTCCTCGCTGCGGACCTGCGAGCAGGGAAACGCTCCCCTCACAGACGCGTTGAATTGTCCCCACGCCGCGCCCCTGTAGAGAAATATAAAAGGTTAGGATTTGCCACTGAGGTTCTTCTTTCATATACTTCCTTTTAAAATCTTGCTAGGATACAGTTCTCACATCACATCCGAACATAAACAACCATGTTATTACCATTAACAAAGCTAAAACCGAGAGCAAAAGTTGCTGTTGTAGGGGGTGGCGTTTCTGGACTATGTTTTACTTATTTTTTAAGCAAGTTGAGACCGGATGTTGAAATCACACTGTTCGAATCGCAGAACAGAACTGGGGGTTGGATATATTCTTGTAACACAAGAGATATGAGTGGGAACCCAATTATGTTGGAGAAGGGACCCAGAACATTGAGGGGCGTATCAGACGGGACCGTTCTGATTATGGATACCCTTAAAGACTTGGGCAAGGAAGCAGTTATTCAAAGCATTGATAAAGGTTGCATTGCAGACAAAAAGTTTCTACTAGACCCCAGTGATAAACTCGTGCAGGTTCCTAATTCGATATCTACAACAGTAAAATTTCTGCTGAATCCGTTGGGAAAAGGACTCATCACGGGTATGATGGGAGAATGGTTCAGAAAAAAATCTCCACATCCTGGCCAAGACGAAAGTGTCGAATCCATTTGTGACAGAAGGTTTGGGAATAACTACATATCAAACAATATGATCAGTGCCTTACTAAGAGGTATCTATGGGGATGACGTTTCCCTATTAAGTGCCAAGAGAACGTTTAAGAAAATATACTACAATGAACTAAAGCACGGATCTAACACACAAGCTATGATTGATAATATGCGCGGAAAGTCAAGAAGTAAAAAAACTGAGAACCTGCACCAATCTTTAACTGGCTGCCTTAACGACTACTCAAATGCGTTTGGAAAAGACAGGTCAAAGTTATTAGACTTATCCAACACGCTAAAGAAATATCCCATGTTGGGTCTTGCTGGGGGACTAGAAACGTTTCCCAAGATAGTCAGAAATGCTTTGAACGAATTTAAAAACGTCAAAATAGTTACTGGGAACCCGGTTACGCAAATAATGAAACGCCCTGCTAACGAAACGACAATCGGATTGAAAGCGAAATCTGGCGACCAATACGAAACATTTGACCATTTAAGACTTACGATAACACCTCCCAAAATCGCTAAATTGCTACCGAAGGATCAAAATTCATTATCCAAGTTATTAGATGAGATACAATCAAACACAATAATTTTAGTTAATTATTATTTGCCAAACAAAGATGTAATAGATGCCGATCTACAAGGCTTTGGATACTTGGTTCCCAAATCCAATAAGAATCCAGGGAAATTGCTTGGTGTAATTTTCGATTCTGTTATCGAAAGGAATTTCAAACCACTTTTTGACAAACTCTCCACAAACCCAAACGCCCTCAACAAATATACAAAAGTGACTGCGATGATAGGAGGTTGTATGCTCAATGAACACGGTGTTCCTGTAGTGCCATCCAGGGAGGTAACCATTAATGCAGTCAAAGATGCGCTGAACAACCACCTCGGCATCAGTAACAAGGATCTGGAAGCTGGTCAGTGGGAATTCACTATCGCCGATAGATGTCTGCCAAGATTTCATGTAGGTTATGACGCATGGCAAGAAAGAGCTGAAAGGAAGTTGCAAGAATCTTACGGCCAAACAGTTTCTGTGGGGGGAATGGGATTTTCTAGAAGTCCCGGTGTCCCTGACGTTATTGTAGACGGCTTTAACGACGCCTTACAGCTAAGCAAATAATCAGTACTGACAATAAAAAGATTCTTGTTTTCAAGAACTTGTCATTTGTATAGTTTTTTTATATTGTAGTTGTTCTATTTTAATCAAATGTTAGCGTGATTTATATTTTTTTTCGCCTCGACATCATCTGCCCAGATGCGAAGTTAAGTGCGCAGAAAGTAATATCATGCGTCAATCGTATGTGAATGCTGGTCGCTATACTGCTGTCGATTCGATACTAACGCCGCCATCCAGTTTTTAGTTTGTTTAGAGATGGACCATGGACCATGGACGGTTAATGGTTTATATACAGCATGCACCCTTTGCCTTAAAACGGTAAAATGAGATAATGCCTTTCCGTTGTATCCCAGCCAGGCCAACCTCCCAGAGTATGTGCGGAGGGTAACGTCACGTAATGTAGAAAGTATTTCTGTATAGGTAGGGTACCCACCCGCATATGAAATTACTCTGTAGTTCCTGGGTCCCGTCGCCTCCGATATTCTTCTGCTTGATTTCTTTTCTGCCGTTTGCTAGTTTTCTTCAACCCCGGTTACGTTTATGCTTTTTCCTTTTGCTCTAGAAGACGGGTTTTTTTGCTCTTAACAAAGGCCAATCAATGCGATGGACTGGTCAATCTCGGCCGGACAGAGACAAACCCATCGTTCTTGACGATTGGGTTACAGTTCGCAGAGAGAAATGTAGTGGAATGGAAAGAGATCGCAGCAGTATATAGTCAGTTTATTCAGGTTACGCTATGGCATTTTTTTTTTTTGTTACGTAACTTGAGTACGGTGTTAGGTTTCTCTTTCTTTAGAAATTAGGGATGCTTCGGACAATTGGGGTTTTAGAAGAAGGGATACAAAGGAGTAGCATGCGTCGGGTGGTAGAGCTAATGTTAGCTGGTTGGGCTGGCCTGCCGGCTAGAATGGTTAATTGGTAGACCAGGTTTTTTTTTTTTCCCTTACTTCATTCCCTTACGTCTTTTGGGGCGACACAGGGTTTTGTATGAAGAGAATTGCATTTGCTATTAGTTAAGGCTTCTCCGAACCTGTATAAAAGTGGAGAGTTCGCAAGGTATGTAATTATATATACAGGATATATTTCCCTGCAATGCTAGTATTTAATGATGATAGACAGTTGGTTTTACAGGACAAAGATTCGTTTGGTCCATTGATTGAATAGCACACACTTCTGACTACGTAGTTGTTTAACAAGATCGAAGTGAATCTGACTCTAAGAATACAGTATACGAAACAATGCTTTCCAGAACAATCCGTACACAAGGTTCCTTCCTAAGAAGATCACAACTGACCATTACAAGATCATTTTCGGTTACATTCAACATGCAGAATGCACAAAAGAGATCACCCACAGGAATTGTTTTGATGAACATGGGTGGCCCCTCTAAAGTTGAGGAAACATATGATTTTTTGTATCAATTATTTGCCGATAATGACCTAATTCCCATTAGTGCTAAGTATCAGAAGACAATTGCTAAATATATTGCTAAGTTTCGTACCCCCAAGATAGAGAAGCAATATAGGGAAATTGGTGGGGGCTCCCCAATCCGGAAATGGTCTGAGTATCAAGCCACTGAGGTCTGTAAAATCTTAGATAAAACCTGTCCAGAAACGGCGCCTCATAAGCCTTACGTGGCGTTTCGTTACGCAAAGCCGCTAACCGCAGAAACTTATAAACAAATGCTAAAAGATGGCGTGAAGAAGGCAGTGGCCTTTTCTCAATATCCTCATTTCTCTTATTCCACTACCGGGTCATCCATCAACGAATTGTGGAGACAGATTAAGGCATTGGACTCCGAGAGATCTATATCTTGGTCGGTTATTGATCGTTGGCCTACAAATGAAGGTCTAATCAAGGCCTTCTCCGAAAATATCACCAAAAAACTACAAGAGTTTCCGCAACCTGTCAGAGACAAGGTTGTTTTATTGTTTTCCGCACATTCTCTACCCATGGATGTTGTTAACACCGGTGATGCCTACCCAGCTGAGGTAGCTGCGACGGTTTACAACATCATGCAAAAATTAAAGTTTAAAAACCCTTATAGGTTGGTTTGGCAATCCCAAGTTGGACCAAAACCATGGTTGGGAGCGCAGACAGCTGAAATTGCGGAATTTTTAGGCCCCAAAGTTGATGGCCTAATGTTTATTCCTATCGCCTTTACCTCTGATCATATTGAAACATTGCATGAAATTGACTTAGGCGTCATTGGGGAATCGGAATATAAGGATAAATTTAAGAGATGCGAATCTTTAAATGGCAACCAGACCTTTATTGAAGGCATGGCAGATCTCGTCAAAAGCCACTTACAGAGTAACCAACTCTATTCTAATCAACTACCTCTTGATTTTGCACTTGGCAAGTCCAATGATCCTGTAAAGGACCTTTCATTGGTATTTGGCAATCACGAATCTACTTGAAGATAAGACAGGGGAGAGGGATTCTTCTTCTTACCGACTCAACGCACCACACACCCTCTTTCCCAATTCTAATCATTCGATCTATGTAAACTTATCGTAACTAAACTAACTAACTACCAAGATTCTTCTTTACTTTACAGAGGTGTATTTCTTTAGTTGTCACATACAAAAACGCACACACACACACACATTTTGTCCTGTCCAATGACAAAATGTGACAGGAAACCCGAACGTCGGAGGTTTTCGGGTGGCAGTCATGTCGCCCAGTACTACTCGTAGCAGTAGTACTACTACCATTACACAAGGGCCAAACGTCAATGCTTTGCCGCGGGAGAGCCTGACCTATTGCATCTCCCGCCGTGCACAGGGTGATTTAAAT

[C4+Down] module :P_ADH1_- *HEM1_Km_* -T_ADH1_, P_PDC1_- *HEM2_Km_*-T_PDC1_, P_PGK1_- *HEM3_Sc_* -T_PGK1_,

P_ENO2_- *HEM4_Km_* -T_ENO2_, P_INU1_- *HEM12_Sc_* -T_INU1_ , P_AFT1_- *HEM13_Km_* -T_AFT1_, P_TEF_- *HEM14_Sc_* -T_TEF_, P_OM45_- *HEM15_Sc_* -T_OM45_

GAGCTCGaagaaatgatggtaaatgaaataggaaatcaaggagcatgaaggcaaaagacaaatataagggtcgaacgaaaaataaagtgaaaagtgttgatatgatgtatttggctttgcggcgccgaaaaaacgagtttacgcaattgcacaatcatgctgactctgtggcggacccgcgctcttgccggcccggcgataacgctgggcgtgaggctgtgcccggcggagttttttgcgcctgcattttccaaggtttaccctgcgctaaggggcgagattggagaagcaataagaatgccggttggggttgcgatgatgacgaccacgacaactggtgtcattatttaagttgccgaaagaacctgagtgcatttgcaacatgagtatactagaagaatgagccaagacttgcgagacgcgagtttgccggtggtgcgaacaatagagcgaccatgaccttgaaggtgagacgcgcataaccgctagagtactttgaagaggaaacagcaatagggttgctaccagtataaatagacaggtacatacaacactggaaatggttgtctgtttgagtacgctttcaattcatttgggtgtgcactttattatgttacaatatggaagggaactttacacttctcctatgcacatatattaattaaagtccaatgctagtagagaaggggggtaacacccctccgcgctcttttccgatttttttctaaaccgtggaatatttcggatatccttttgttgtttccgggtgtacaatatggacttcctcttttctggcaaccaaacccatacatcgggattcctataataccttcgttggtctccctaacatgtaggtggcggaggggagatatacaatagaacagataccagacaagacataatgggctaaacaagactacaccaattacactgcctcattgatggtggtacataacgaactaatactgtagccctagacttgatagccatcatcatatcgaagtttcactaccctttttccatttgccatctattgaagtaataataggcgcatgcaacttcttttctttttttttcttttctctctcccccgttgttgtctcaccatatccgcaatgacaaaaaaaatgatggaagacactaaaggaaaaaattaacgacaaagacagcaccaacagatgtcgttgttccagagctgatgaggggtatctcgaagcacacgaaactttttccttccttcattcacgcacactactctctaatgagcaacggtatacggccttccttccagttacttgaatttgaaataaaaaaaagtttgctgtcttgctatcaagtataaatagacctgcaattattaatcttttgtttcctcgtcattgttctcgttccctttcttccttgtttctttttctgcacaatatttcaagctataccaagcatacaatcaactATGGAGTCTGTTGTTCGTCAATCTGCAAGGGTTTGTCCCTTCATGAAGTCTGCTACAGGGTCTGTGCAGAATGTGAAGGCTTTGAAAAACGCCAACTTGCCAGCTATTGCGCAAAAGTGTCCGTTTATGGGTCGTGCTATGGAGCAGCGTAGAGCATATGCGTCAGCATCCGGGTCAGGAGCTGGGTCAGGAGCTGGTGCAGCAGCAGCGGCTTCTCCAGCAGCCGTTGAAGCGAGGAACGCAAGTACTGCTTCTGCAGATGCTACTGTTTTGGACCATGCTACCAATGAAGCTTCCTTTGACTACCAAGGATTATTTGAGTCTGAGTTGGCCAGAAAGAGAATGGACAAGTCGTATCGTTACTTCAACAACATCAACCGTTTGGCAAAGGAATTCCCAATGGCCCACAGACAACAAGAGGACGATAAGGTCACTGTGTGGTGCTCCAACGATTACTTGGCTCTTTCCAAGAACCAAGAAGTCATCGATGTGATGAAGAAAACTTTGGACAAGTACGGTGCAGGTGCTGGTGGGACCAGAAATATCGCAGGTCACAACAAGCACGCGTTGCAGCTAGAAGCAGAAATCGCAGCCTTGCACAAAAAAGAGGGTGCGTTGGTGTTCTCCTCTTGTTTTGTCGCTAACGATGCCGTCATCTCGTTGCTCGGCCAGAAGATTAAGGATCTAGTCATCTTTTCCGATGAGCTGAACCATGCCTCCATGATCGTGGGTATTAAACACGCCTACACCAAGAAGCATATCTTCAAGCACAATAACTTGGAAGAATTGGAAAAATTGCTAGCCATGTACCCAAAGTCCACTCCGAAATTAATCGCCTTTGAATCTGTTTACTCTATGTCTGGGTCCGTCTCTGATATCAATAAAATTTGCGACTTGGCAGAAAAATACGGTGCCTTGACTTTCTTGGATGAAGTTCACGCAGTTGGTTTGTACGGTCCACATGGTGCCGGTGTTGCAGAGCATTGTAACTTTGAAGCTCACCGCAAAGCTGGTATTGCATCCCCAGAATTCCGCACTGTCATGGACCGTGTCGACATGATCACCGGTACCTTGGGTAAATCTTTCGGTACTGTCGGTGGTTACGTTGCTGCTTCCTTAAAATTAATCGATTGGCTCAGATCTTACGCACCAGGATTCATCTTTACCACATCTCTACCACCATCCGTTATGGCGGGTGCCGCAGAGGCTATCAGATACCAACGTTCTCACTTGGACCTAAGACAAGACCAACAAAAACATACAACTTACGTCAAGGAGGGATTATCCGATCTGGGTATTCCAGTGATGCCAAACCCATCCCACATTGTCCCAGTTTTGGTTGGTAACCCTCATTTGGCTAAAGAGGCTTCGGATATCTTAATGCATAAACATCGTATCTACGTTCAAGCAATCAACTTCCCAACGGTTTCCAGAGGTACCGAACGTTTAAGAATTACTCCAACTCCTGGTCATACTAATGATTTGTCTGACATCTTGTTGGATGCTATGGAAGACGTTTGGTCAACCCTACAATTACCAAGAGTTCGTGACTGGGAAGCCCAGGGCGGCTTATTGGGTGTTGGTGATCCAAACCACATCCCTGAACCAAACTTGTGGACTGAAGAACAATTATCTTTGTCTAACGATGACTTGAACCCAAATGTCAAACATCCAATAATAGAACAATTGGAAGTTTCTTCTGGTATTAGATATTGAgcgaatttcttatgatttatgatttttattattaaataagttataaaaaaaataagtgtatacaaattttaaagtgactcttaggttttaaaacgaaaattcttattcttgagtaactctttcctgtaggtcaggttgctttctcaggtatagtatgaggtcgctcttattgaccacacctctaccggcaTGAATAATGAATGGCCTTGTATTCGTTTTTTTCCGAGAGAAAATTAACAAGAGCGAAAAAAAAAACGGGCTTCGGTGAAAATCGGGTGAATATGCAACTAGCGGGACGAATGCTCTGGAAATGCATATCCTATGCAACTAGCGGGATGAACAAATCTCACCCCAGAATTCGCAGGAAAAAACAGGAAAAAAAAAAAGAAGGCCACCACGGCCACAAAGACCACAAAGGCCACAAAAAGAACAAAAAAACAACCGTCCCAGCTTCCAGTGTTTGGAATACTGGAACACAGGAAGCCGCATAAGAGTGGGCGTTGCACAGGAAGCCAGGCCCAGAAGCCCCAGAGTTACTTTTTTTTTTTTGTTTTTTCCTTCTGTTCGCTGTGCCCGCATCAGATGATGCGCCTTTATTTACGATGCCAATGCGAATAGCACCAGTGAGAGCACCAGTAAAAGCATACGCATACACATACACACATAGAGCAAGCAAGCAGGCTAGCAACCAGGAAAGGCTGCCAGTGACTGCTACTGGGTGTCTAAGAACCGTAGGGCGGATTATTGTTGCGGTGGTTGGTTGCGGGTGGTTATGCGATGGTACGGTGCAGAATCGTACGGTGTTGGTTATGGAATTAGTATGGGTATGTGATATGTGGTAATATGTGATATTGGGTTATTGTGATTTGGAATACTGAATATCGAATATGGGATATGGAATATGGCCATGGCATGGTATGGTATGGGATGGGAGTATTCTATTTTATTTTATTTTATTCTGGTTCCTGCGTTTAGGGTAGGGTAGGAAGAAGGTGAGTGCTTTTGTATATAAGTGGAGTGTCTGGATCAGTTTTGTGGATTGTGAATGTTAGTTTCCCCTTTAATGTATATTTGTATTATTTGCTTTTGAGTACTCAATAACCAAGCACAACTACTAGTTTTAAAGGATCCATCCTCTTAAACAGTACAAATCGCAAAGAAAAGCTCCACACCCAAACCAAATAATTGCAATGGTTCACGTTGCAAAATTTTTAGACGAACGTCCTACTGAAATTCCTTCCATCCTTGCTGGTGGATACAACCACCCTCTATTGAGAGAATGGCAGAATGAAAGACAGTTGACTAAGAGCATGTTCATTTTCCCGCTATTCATTAGTGACCAGGACGAGGAAGAGACCCTGATACCATCGCTACCAAACATCAAGAGATTTGGTATCAACAAATTGAAGGATTATGTTGCTGGTTTGGTTGCCAAAGGTTTACGTTCGGTGATCTTGTTTGGTGTTCCATTGAAGCCTGGTGCTAAGGACGAGGTTGCAACCGCAGCTGACGATCCAGATGGGCCTGTCATCAAGGCTATCAAGTTGTTGCGTAAGGAATTCCCAGATTTGTACATTATCTGCGATGTGTGTTTGTGTGAATACACTAGTCATGGTCACTGTGGGATCTTGTATGAAGATGGTACTATCAACAGAGAAAAATCTGTGCAAAGAATTGCTGCTGTTGCTGTGAACTATGCGATAGCTGGTGCGCACTGTGTTGCCCCAAGTGATATGATTGATGGTAGAATCAGAGAGATCAAAATGGGATTGATCGAACACGGATTAGCCCACAAGACGTTTGTTATGAGTTACTCTGCCAAGTTTAGTGGTAATTTGTACGGTCCATTCAGAGATGCAGCATGCTCGCAACCAGGTCAAGGTGATCGTAAGTGCTACCAGTTGCCTCCAGGTGGTCGTGGTTTAGCCAGACGTGCATTGAAAAGAGATTTGGCTGAGGGTTCCGATGGTATTATCGTCAAGCCTTCGACCTTTTACTTGGACGTTATGTCCGATGCTGCTGAAATTTGTCGCGACATTCCAGTTTGCGCATACCACGTCAGTGGTGAATACGCAATGTTGCATGCTGCTGCTGAAAAGGGTGTTGTTGACTTGAAGAGCATTGCTTTTGAATCCCACTACGGTTTCTTGAGAGCTGGTGCTCGTTTAATCATCAGTTACCTCACCCCTGAGTTTTTGGAATGGTTAGATGAAGTGAACTAAAGAGGGAGAGGATAAAGAGATAAATTACGATTTTGGATTTTAATGATTTTATAAACAACAACAACCAACCAGCCTTTTACTTTATTTGGCATATACACAAGCTTACTCCATTTCATTGATTATCTATGTGTATATATATAAGTGATGTATAACAATTATTATTATACATAGATAATATTTTTATGATATGTTTTTTCTGAGTTTTGATATTATTTATTACAAGTTACAAGTTACAAGTTACAAGTTACCAGGAAGAATTAAATAAAGGTAAATTGGGGGAATTATAAGCGTATGGGCATAGCCTTGTCCAAGGCCTTGGCCTTGGTCAATGCCCTTATTCTCTTGTTTCCATTGCCTGTCGATATACGACTTCAAGGCCATGTACCTGAGATCCTCATCTGGGGCCTGAAGATCCATTGGTGAGTGCCACTGGCTGCTGTGCCGTTGTGAAAAGGCCCTGTGTAGCAGAGAGGGAGGTGTGTATAGGTATATATATGTATATATATGTATATAAGTGTGGATTTAACACACTCTCTCTTCTTTCCCATGCCATGCCAGTCACGTGTTCCACACGTGACTGTACTTACCCTCACATTACCCTCACTCTTTCACATTACCCTCCCCATCCCATCTTCTTTGGTTTTCCCCCCCAGCTTCCTGTCTTCCACTATCCCTGCAACCACCACCACCACACAACAAACATCCCCCAGCAGCCCCCCAGCAGCCCGGAAAAATCCACGCCTCCCCCCGAACACACGAAGCCCGCCCCGGCTTCCACTCCAACACTGCCAACACTGGAACCCCGCACCACGTAACCACCCACTTTAGTGGCTGCCCGCCCCTCCTGCGCTTCCCTGGGGCTTCCCTTGCGACCACACTCCGGTGGCATGGCCCTCGCTTGCTACATGCTACCTTTTTTTTGCCCTTTTCTGGCCTTTTCTGGCTTTTCTGGCCTCACCACAGTGTACCACAGTGTAGTAGTAGTGTAGCATTGTCGAGTGGTATTGTCGAATGGTATTGTAGCCTGTTGTAGCCTGTTGTAGCCTGGTGTAATGTCTGGCATAGTCTGGTGTCTGGTGTCTGGTGTCTTTTGGAAACTGAACAGTGGAAAGTGGAAGTGAAAAATTGTATAAATATAGGTGTCCCATTCGTGGTTTGGTGGATGTGTCCTTGACATTGCAGGTTTCTCTGTTTCGCTGTGTTAAGACTTGTTAACACTTGATAAGTATTTCCCTAGTAATACCCCACCAACCATCGGTTGATACATTTTAATACACACTACCTATATAAAGATACAAAAATGGGCCCTGAAACTCTACATATTGGTGGGAGAAAATCGAAATTGGCGGTAATACAATCCAACCATGTTTTAAAACTGATCGAAGAAAAGTATCCGGACTACGACTGCAAGGTTTTCACTTTGCAAACTCTTGGTGACCAGATTCAATTCAAACCTTTGTACTCATTTGGCGGTAAAGCTTTATGGACAAAGGAGTTGGAAGACCATCTTTACCATGACGATCCCTCAAAGAAGCTTGACTTGATCGTTCATTCTCTGAAGGACATGCCCACTTTACTACCAGAGGGTTTCGAGCTGGGGGGTATCACTAAGCGGGTCGATCCAACAGATTGTCTTGTCATGCCCTTTTACTCTGCTTATAAGTCTCTGGATGACCTTCCAGACGGGGGGATTGTGGGAACCTCATCCGTGAGAAGATCTGCTCAGCTAAAAAGAAAATACCCACATTTGAAATTTGAAAGTGTCAGAGGAAATATACAAACTAGATTACAAAAACTAGACGACCCAAAATCTCCGTACCAATGCATCATCTTGGCGTCTGCTGGGTTGATGCGTATGGGGTTGGAAAACAGAATTACGCAGCGATTCCATTCGGATACAATGTACCATGCAGTTGGACAAGGCGCCCTGGGTATAGAAATTAGAAAGGGTGACACCAAGATGATGAAGATTCTTGACGAAATTTGCGATCTAAATGCAACTATATGTTGCCTTTCGGAGCGTGCTTTGATGAGAACTTTAGAGGGGGGTTGTTCCGTTCCTATTGGTGTGGAATCTAAATACAATGAAGAGACTAAAAAATTACTATTAAAGGCCATTGTAGTTGACGTTGAAGGCACAGAAGCAGTAGAAGACGAAATTGAAATGCTAATAGAAAATGTTAAAGAAGATTCCATGGCGTGTGGTAAGATACTAGCTGAAAGAATGATTGCCGATGGCGCAAAGAAAATTCTGGATGAAATTAATTTAGACAGAATCAAATGAGTGCATTGTTGAACTTTCTAATGAAACCACACGTGAATTAAAGGAAAGCGCGCAGCAGCAGCAGCAGCAGCTTTTCTTTCCTTCTTCAAATCATACCTTTTCTCTATTTCAACGTTCTTTTCATTTCACCATGAAATGAGTTGAATTGTTGCTTTGAGTTTACGACGATACATAAATATTAATTAAAATACGAAAAATACACATTTTATTTACGACACTTGCCTCTCCCCCTAGATGTTACGCTGCTCAGGTTCGAGCCGCTCGATCCCTAGCATATCGAGAACTTCGTACTCGTCCCCCATTTTCAATTTCCCAAAAAAACAAGAAGCCGGAATCCACCGGAATCCACTAATACGCAAAAAAGACGACGCCAGCTTCGCCGTTGGGCATTGGCACCATATTCCATCGTTCGTTTGACTTTGGTTTTGGTTTTAGGTTCTGGCTTCGTGTTTTCCCCTCACTGCCCTTGTCCTTGCCGTGCTCCCCCTCGTTCTCTGCGCAAAGTCTGGTGACCCTCCGGCTGCCACAAAATCCCGAGGCACTGAAAGGGTGGGGCCTCGGGGGTCTCGGGGCAGAAGCCTTGAGCTTCGTGTTTATTCACTCTGTACGGGCGGTTCTAGCGTTTCTGGGAATCCAAAGAGGAAGGGCCGTGTTTCGTGCAGGCATTTTAGTGGATGCCCGGGATGTAAGCTCTTCGGTTACAGAAAATCAGGTTGTGATTCCAGTATGAGGAATAGTGGATGCCAGGTTGTGCGAGCATCACCAATATCATGGGAAATGCGAAATGGGCATGAGCCCAACCAACACAACACAATACCATACGCCAAATGGTAGCCTGAAAAAAAAAAAAAAGGTCTTGCCAGACCACTAAACTCGAGGTAGAACAAGACTGAGAAAGAGTGTGTGATCCCTTTGGTGGTAGTAATTTTTTTTTTTTTTTTTCAAGTTTCCAGCATCCCAAACCGAAACCCAACATCATCATGATCCCATCCCATGCCTATGCCCATGCCACATTCATTCATTGCACAACACACAACGTAGTGGACGACAGCATAGCATCAGTTAACTAACGGCCACTTGTTTTTTTTTCTTCCATTTTTTCGTTTTGTTCTAGCAACAATGAGTTCTAATTTTTTTGTTATAAAAGGGACAGTTAAGGTATATGTAAGGTTTCTTGTTCCATTTGGGTTAGAGTTTTGTAGTATTAGTTTTGGTTCTTAGTTCTTTTGGCCTAAGAAACCAGTAAGAATTCTTCTGATTTTCTTTACAAACACATCAACAACAACAAACACAAACTACAATGTCCAGAATAATTTTTCTTAAGAACAAAACAACACCTACAGACCCGTACGAAACCACTTTTACAGATGGAGGGTTTGAACCTGTATTTGTTCCACTAATACGCCATTTCCATGTTCCAGACGAAGCGTTGACGCTATTCAGAAACAGGAGCTACCTAACGAAATTGAAATACATTATAGTTACTTCACAACGAACAGTCGAATGTCTAAATGAGTCAATACTTCCTAAAATGACTACGGAAGAGCAATCTCTTTTAAAGGAAAAGACTATATACACAGTTGGACCGGCCACTTCGGAGTTCTTGAGAAATTCTGGTTTTCAAAACATAAGAGGAGGAGTTGAAGTTGGAAATGGCGGATTACTTGCTGATCTGATTGTTAAATCGCATTCTGAGGAAGAGATTGATCACTTTTTGTTTTTAGTTGGAGAAATAAGAAGAGATATCATTCCCAAGAAACTTAAAGCTAATGGCTACAAGGTCAACGAAATTGTAACTTACAAAACCGAGAATCTGTTAGACAATATTGATCGTTTTATTTTGCACTATAAGAATGATGAGAATAACCAAGCGGTTATGAACAAGCCCTCCACATGGATTGTGTTCTTTAGTCCCCAGGGTACGGAAGATATAATTGAGTACCTAAAAGAGAACAAGGGCTATAAAATTGCTAGCATAGGTCCTACAACGGAAAAGTACCTCCTAGAAAAAGGTTTGAAACCAGACACTGTTAGTTTTAAGCCGTCTCCAATATTCTTACTGAATGCCATTAATAGTTACAAATAAATCTAGCTGACGCGACGCTTTTTTTCTCCAACTAAGGGAATCCAGGGAAACCCCCCTCGAGCCCCAGTTTTGGTTAGAAATTGTCCTTTTTTATACTATTGAATGACCTAACTAAATCTATATAAACCTTTTTAACGTCTAACGACTCTTTATTATTGAAGTGGGAAGTCTTGGGATGGGAAGTCTTGGGATATTTCAGGCCTTTGGCCAGGCCAAAGGCCAGGCCTTGGCCCAAGGTACCACCCCCTTATATATGCTCAGCCAATTGACTCAGCTGTCCCTCCACACTTTATTCCTGTAATCCGAAAAGGTAAACAGACACAAAAACGACAAGAGAAGCAAACACAAAAAAAAAAAAAAAAAAAAACAAAAAAAAAAAACACAAACACAAACACAAACACAAAAACGCTAAATTATGCACACAAGGGCCGGCGGGGCTGCCGGAAAAAAAAAGGGAAAAATACACAGACGAGCGCGCACAGATGGGGTTACCACTGCAAGTTACAAGTTGCAAGTTGCACGCTGGAATCAGAATTGGAATCAGAATTGGAATTGGAATTAGAATTAGAATTAAACTTGGGGTAGCCACGGGAACGGGATAACTCAGGAATCGCTCGCAGGCGTCTCCGTCTAGGCAATCCCAAGGTAAGCCTAGGCACTCCCACAGGGGAAAGAACGGTTGAAGGCAAAGTAGTGCTAACAATTGGTAACGAATGGTAACAAGTGTGTCCGTCTCCACCTGACATTTGCTAGAGCTGGGGATTCCACATTCTTGTGCTCTGAATTCTCAAACCGAAATGGGGCGTTGTTACCCCAGGTATCCGGTTGTAGTTGGCACTGGGGATGGAAAAAAATGATGTTGATGTTGAGTTAGTTGGGTTGAGTCAATTAGTGCGTGAAAGTATCACCACTTTTGTCATCCGGCGTTTCTGTGCGAATCACACACACACACACAGTTTATTGGAGCACTTGTTTCTGGCGTATTCGTAATTGTTCTGCGGTGCGGTTCTGTGTGCATTTTTCCTGGGGTGTCTGCCGCACCTACTCATCACCCACGCCGTGGGTTTGAGCCATGGCGGAGGTACGACTGACTGGCTGCCTGCCTGCCTGACTGACTGCCTGACTGCAGGAAAAGAGGGTTTCGAAGGAAAAACTTTTCCTGTGTTAATCCGGCCGTGCGCCGCTGCTCCAAAATCCACCTTCATGAGAAGGAGTTTGAAAAAACAAAAAAATTCACATATAAAAAGCGTATCTCGAGATCTCAAAGTCTCCCTTGAATCGTGTTTGCCAGTTGTAACTCATCCTTTATTCTTCTATTCTATCTCTCTCTTTCCTTCCCCTAATCAGCAATTAAATCCGGGGTAAGGAAGAATTACTACTGTGTGTAACGGTTATATTTCGTTTTTTATTTTTTTTTTCCATTGCCATAGAGAAAGAAAAAAAAAAAAAAGAGAGTTTGTGAAGATCTTCCATTCGAATCCCATAAGTGACACATTTAATTTTTTTTTTGTTAGATATGGGTAACTTTCCAGCTCCAAAAAACGATTTGATATTGAGAGCCGCAAAGGGTGAAAAAGTCGAGAGACCGCCATGCTGGATAATGCGCCAGGCAGGTCGTTACCTGCCGGAATATCACGAGGTGAAAAACAATCGTGATTTCTTTCAAACTTGCAGGGATGCGGAAATTGCTTCTGAGATTACTATCCAGCCGGTAAGACGCTATAGAGGCCTCATTGATGCTGCTATTATTTTTAGTGATATCTTAGTTATTCCGCAAGCCATGGGTATGAGGGTCGAGATGCTCGAAGGTAAAGGTCCACATTTCCCAGAACCTTTAAGAAATCCGGAAGACCTCCAAACGGTATTAGACTACAAGGTTGATGTTTTGAAAGAGTTAGATTGGGCTTTCAAGGCAATCACCATGACAAGGATCAAGTTGGATGGTGAGGTTCCCTTATTTGGCTTTTGCGGGGGACCTTGGACTCTAATGGTTTATATGACGGAAGGCGGTGGATCCCGTCTTTTCAGATTTGCCAAACAATGGATTAACATGTATCCAGAGCTTTCTCACAAATTATTACAAAAAATCACTGATGTGGCCGTGGAGTTTCTGAGTCAGCAAGTCGTGGCGGGTGCTCAAATACTACAAGTTTTTGAAAGTTGGGGTGGTGAGCTTTCGTCTGTAGATTTTGATGAGTTTTCCCTACCATATTTAAGACAAATTGCCGAAAGAGTGCCTAAAAGATTGCAAGAATTAGGTATCATGGAACAGATTCCTATGATCGTTTTTGCGAAAGGGTCGTGGTATGCTTTGGACAAGCTATGCTGTTCAGGATTTGACGTTGTTTCGTTGGACTGGTCCTGGGACCCAAGAGAAGCGGTAAAAATAAACAAGAACCGTGTCACCTTGCAGGGCAACCTGGATCCTGGCGTCATGTATGGTTCTAAAGAGGTAATAACAAAGAAAGTTAAACAGATGATTGAGGCTTTTGGAGGTGGGAAGTCCCGCTACATTGTTAATTTCGGTCACGGTACCCACCCTTTCATGGATCCAGACGTCATCAAGTTTTTCTTGGAGGAGTGCCACAGAATTGGTTCGAAGTAAGGCCGCAAGCTTTGATCTGATCTGCTTACTTTACTAACGACAAAAAAAAATCAAAAAAAAAAAAACAATCAGTCCTTCTCTTCTTACGATATGATATGATTAAATGATGCTATGAAATCATCTTCTTCTTAACTTTCTTAAATCTTACGCGTCACTTACTCTATATACCCGTTTAGCTTTGCCTGGTCACAGCGACATTTTATATAAGTGTACGTATTTTCTTTTTTTTTTTAAAAATTTCTATTCTAACCTTAGAAAAGTGCCCTTTAAACCAGCTGTCCTGGCACTATATCTTTATCATGTGCCGGTCGCTTTCCCTTTCCGTTTCCCTTTTCCTTTCAATTGGTGGCCTGGAATTCCGAACTCATTTTCGCATCTGAAACTAATTCTCGAAACCTTTAACATCAAACAATTGAAAAGATCATCATCACCAGAAATAAGAAAAAGATCAACACAACAGCTAATAACAGTACGAAAGAAAGATCGCTCGAGTGAAAAGGCAGCCAAGAAAGGTCATTCGATTTGGGTCTAGACTGATTATAGACATACCAATTGCACTCAGTAAGAAAATGAGTTTCAAATTTGACGATGACGGTGTGGTAAAAGAATTTCACGGCAACACCATCATATGCCATATTCCTCAACAAACCGAATTCTTCAACAAATTGTTGGACTTCTACCGTTTTGCGAAACGACTTTCCTTCTACGACAAGATCACCCTACTTCCTCCTTCAAGCTACCACGTTACGATCATGAATTGCTGCCACGAACACGATCGTTCTGAGGGCCACTGGCCCAAAGGAATCGATCCGGACACAAGCATGCTGCGGTGTACATCACATCTGACCAACATTCTAGGATCGGTGCCAGGTGAATGTACGTAATGGAGTTTGGATAGATTCGATACAGTTCATTACCAAGAATGGACAGCAGTCCCCCATATACGGGAATGCAACCGGAGGATCATTGAACCAGATTAGCATTGTCGCTCCATACAACCTTAAAGGGTTCTTTGGTAATTGTTCGAATTGGATGGACAGTCTTGGAGTGTTGTACGGGTAATTGGAGCCTGTTTGAAGCGTTTGTTTCCCTTTGGAGCGGTTTCCGTGTCATCAAGATGTCGGGGTCCGTCAAAAAAAATTTAGCGGGCACTCCGGGGTCCGTGGGGTCCGTGTGGTAGGGTGTGCCAGACCCGGTACTCTAAATCCATCCCAAAGAGCAAAGAAAGGGTTGCATGAATAATGGACCCTTGTTGCGAGCAGTTACTGTTGCAAGCAGTTACTGTTGATAGAGATGGTGTTCGCTAGTATACAGGTCAAATACTTAAGGTTATCCTTTGTGTGGGAGGATTTTAGATTACCGAGTCTTTTTTTAGTGGAGTGCAGTGGGATGAGAAAAGTTGGCCCGACCTCGGAGAATTTCTCGTAATATGTTGCTCAATCTATTAAAAAAAAACTCGGATACTTCGAAAGCACCCCGTGTGGTTATCCCATGCGCGTAAAATTCACCCTCTGCGAATCGAGGGCGCCCCTTCAGAAACGGGATAACCGGGTGTTTTAGCCCAGGGCAATATCCAATAGGGCTAACCTAGTCGCTTTCCGAGCCCAATTTGTTAGTCTTGGCGCACCCCGATTGCTTGACACGGAGTCCGTCAATACCTATAAAATTGGGATTTAGAGGTTAATTATTTTTTAGGAATCTGTAACCACACCCCTCTTCGGACCCCGCCTAGTATTTTCTTCTCATGCCCGAAATAAATAAAAGGACAATTCTCCCCCAGAATATTACTACTTGTTCATTGTTTTAAAATTCCAACCAGGCTTCGTTACTCCTAAAAAATTTTCGAACCACGTACACCTTCAGCAATGACAGACTCTGCTGTGCCAATGAGAAAGAAGATGGAAGAGTTGATTCGCCGTAAGCAAAAGGAAATCACTAAAGGGTTGGAGGAATTGGACACTGTGCAGTTCCGTGCAGACTCCTGGACCCGTGGTAACGATGGTGGTGGTGGTACCTCTATGGTTTTGCAAAACGGTTCTACTTTCGAAAAAGGTGGTGTCAATGTGAGTGTTGTTCACGGTACTTTGACTCCTCCAGCTATTAGAGCGATGAAAAACGATCACAAGAACTTGCACTTGCCAATTGACCCAGAAACTGGCGAGCCAGATGCTTCTGGTGTCAGATTTTTCGCTTGTGGTTTGTCGATGGTCATTCACCCAATCAACCCACACGCGCCAACAACGCATTTGAACTACAGATACTTCGAGACGATGCATGCGGACGGGACACCTCAGGCCTGGTGGTTTGGTGGTGGTGCCGATCTTACCCCATCGTATTTGTACGAGGAAGATGCAAAGTTGTTCCATCAATTGCACAAGGACGCTCTAGACAAGACAGACGTTACCCTATATCCAAAGTTCAAGAAATGGTGCGATGAGTACTTCTACATCAAGCACAGAGAAGAAACCAGAGGTATTGGTGGTATCTTTTTCGATGACTTGGACGACCGTGATCCAGACGTTTTGTTGAACATGGTCGAAAACTGTTTCGATGCGTTCTTGCCCTCTTACACGGAAATCATCAAGAGAAGAATGAACATGCCATACACCGAGGAGGAAAGACAATGGCAGCAGATTAGACGTGGTAGATACGTCGAATTCAACTTGGTCTTGGATAGAGGTACTCAATTTGGTTTGAGAACTCCTGGCTCTCGTGTTGAGAGTATCTTGATGTCCTTGCCTGTTACCGCCTCCTGGTTGTACGACCACCACCCTGAACCTGGCTCCAGAGAAGACAAATTGCTACAGGTCTTGAAAAACCCAATCGAATGGGTGTAATGGGTGCTTAAGATCTGTCTTTTTTATTTATAATCGGCCCTTTTATCACTTTATTTTTTAGTTTATTTATTACATCTTTCGTCTGTATACTAGTTTGGGTCAAAGAAGTCAAAAATCGCACAATTTGGGTGTAGAACGTAAACGTAGTTAACACCTTGCGCAGCCTCTGCATCAGCGTCTTCGAGATCTTGAACATCTTGTTCGTTGGGTGCAACTTTCATCAAGATCTTGTCATGTACCAATTTACTGAGAACCTTGTGCACCAACTTTCTTTCAGCCCAATATTCTTCCTCTGTATGAAGCTTGCCTTGTCCCCGCCGGGTCACCCGGCCAGCGACATGGAGGCCCAGAATACCCTCCTTGACAGTCTTGACGTGCGCAGCTCAGGGGCATGATGTGACTGTCGCCCGTACATTTAGCCCATACATCCCCATGTATAATCATTTGCATCCATACATTTTGATGGCCGCACGGCGCGAAGCAAAAATTACGGCTCCTCGCTGCGGACCTGCGAGCAGGGAAACGCTCCCCTCACAGACGCGTTGAATTGTCCCCACGCCGCGCCCCTGTAGAGAAATATAAAAGGTTAGGATTTGCCACTGAGGTTCTTCTTTCATATACTTCCTTTTAAAATCTTGCTAGGATACAGTTCTCACATCACATCCGAACATAAACAACCATGTTATTACCATTAACAAAGCTAAAACCGAGAGCAAAAGTTGCTGTTGTAGGGGGTGGCGTTTCTGGACTATGTTTTACTTATTTTTTAAGCAAGTTGAGACCGGATGTTGAAATCACACTGTTCGAATCGCAGAACAGAACTGGGGGTTGGATATATTCTTGTAACACAAGAGATATGAGTGGGAACCCAATTATGTTGGAGAAGGGACCCAGAACATTGAGGGGCGTATCAGACGGGACCGTTCTGATTATGGATACCCTTAAAGACTTGGGCAAGGAAGCAGTTATTCAAAGCATTGATAAAGGTTGCATTGCAGACAAAAAGTTTCTACTAGACCCCAGTGATAAACTCGTGCAGGTTCCTAATTCGATATCTACAACAGTAAAATTTCTGCTGAATCCGTTGGGAAAAGGACTCATCACGGGTATGATGGGAGAATGGTTCAGAAAAAAATCTCCACATCCTGGCCAAGACGAAAGTGTCGAATCCATTTGTGACAGAAGGTTTGGGAATAACTACATATCAAACAATATGATCAGTGCCTTACTAAGAGGTATCTATGGGGATGACGTTTCCCTATTAAGTGCCAAGAGAACGTTTAAGAAAATATACTACAATGAACTAAAGCACGGATCTAACACACAAGCTATGATTGATAATATGCGCGGAAAGTCAAGAAGTAAAAAAACTGAGAACCTGCACCAATCTTTAACTGGCTGCCTTAACGACTACTCAAATGCGTTTGGAAAAGACAGGTCAAAGTTATTAGACTTATCCAACACGCTAAAGAAATATCCCATGTTGGGTCTTGCTGGGGGACTAGAAACGTTTCCCAAGATAGTCAGAAATGCTTTGAACGAATTTAAAAACGTCAAAATAGTTACTGGGAACCCGGTTACGCAAATAATGAAACGCCCTGCTAACGAAACGACAATCGGATTGAAAGCGAAATCTGGCGACCAATACGAAACATTTGACCATTTAAGACTTACGATAACACCTCCCAAAATCGCTAAATTGCTACCGAAGGATCAAAATTCATTATCCAAGTTATTAGATGAGATACAATCAAACACAATAATTTTAGTTAATTATTATTTGCCAAACAAAGATGTAATAGATGCCGATCTACAAGGCTTTGGATACTTGGTTCCCAAATCCAATAAGAATCCAGGGAAATTGCTTGGTGTAATTTTCGATTCTGTTATCGAAAGGAATTTCAAACCACTTTTTGACAAACTCTCCACAAACCCAAACGCCCTCAACAAATATACAAAAGTGACTGCGATGATAGGAGGTTGTATGCTCAATGAACACGGTGTTCCTGTAGTGCCATCCAGGGAGGTAACCATTAATGCAGTCAAAGATGCGCTGAACAACCACCTCGGCATCAGTAACAAGGATCTGGAAGCTGGTCAGTGGGAATTCACTATCGCCGATAGATGTCTGCCAAGATTTCATGTAGGTTATGACGCATGGCAAGAAAGAGCTGAAAGGAAGTTGCAAGAATCTTACGGCCAAACAGTTTCTGTGGGGGGAATGGGATTTTCTAGAAGTCCCGGTGTCCCTGACGTTATTGTAGACGGCTTTAACGACGCCTTACAGCTAAGCAAATAATCAGTACTGACAATAAAAAGATTCTTGTTTTCAAGAACTTGTCATTTGTATAGTTTTTTTATATTGTAGTTGTTCTATTTTAATCAAATGTTAGCGTGATTTATATTTTTTTTCGCCTCGACATCATCTGCCCAGATGCGAAGTTAAGTGCGCAGAAAGTAATATCATGCGTCAATCGTATGTGAATGCTGGTCGCTATACTGCTGTCGATTCGATACTAACGCCGCCATCCAGTTTTTAGTTTGTTTAGAGATGGACCATGGACCATGGACGGTTAATGGTTTATATACAGCATGCACCCTTTGCCTTAAAACGGTAAAATGAGATAATGCCTTTCCGTTGTATCCCAGCCAGGCCAACCTCCCAGAGTATGTGCGGAGGGTAACGTCACGTAATGTAGAAAGTATTTCTGTATAGGTAGGGTACCCACCCGCATATGAAATTACTCTGTAGTTCCTGGGTCCCGTCGCCTCCGATATTCTTCTGCTTGATTTCTTTTCTGCCGTTTGCTAGTTTTCTTCAACCCCGGTTACGTTTATGCTTTTTCCTTTTGCTCTAGAAGACGGGTTTTTTTGCTCTTAACAAAGGCCAATCAATGCGATGGACTGGTCAATCTCGGCCGGACAGAGACAAACCCATCGTTCTTGACGATTGGGTTACAGTTCGCAGAGAGAAATGTAGTGGAATGGAAAGAGATCGCAGCAGTATATAGTCAGTTTATTCAGGTTACGCTATGGCATTTTTTTTTTTTGTTACGTAACTTGAGTACGGTGTTAGGTTTCTCTTTCTTTAGAAATTAGGGATGCTTCGGACAATTGGGGTTTTAGAAGAAGGGATACAAAGGAGTAGCATGCGTCGGGTGGTAGAGCTAATGTTAGCTGGTTGGGCTGGCCTGCCGGCTAGAATGGTTAATTGGTAGACCAGGTTTTTTTTTTTTCCCTTACTTCATTCCCTTACGTCTTTTGGGGCGACACAGGGTTTTGTATGAAGAGAATTGCATTTGCTATTAGTTAAGGCTTCTCCGAACCTGTATAAAAGTGGAGAGTTCGCAAGGTATGTAATTATATATACAGGATATATTTCCCTGCAATGCTAGTATTTAATGATGATAGACAGTTGGTTTTACAGGACAAAGATTCGTTTGGTCCATTGATTGAATAGCACACACTTCTGACTACGTAGTTGTTTAACAAGATCGAAGTGAATCTGACTCTAAGAATACAGTATACGAAACAATGCTTTCCAGAACAATCCGTACACAAGGTTCCTTCCTAAGAAGATCACAACTGACCATTACAAGATCATTTTCGGTTACATTCAACATGCAGAATGCACAAAAGAGATCACCCACAGGAATTGTTTTGATGAACATGGGTGGCCCCTCTAAAGTTGAGGAAACATATGATTTTTTGTATCAATTATTTGCCGATAATGACCTAATTCCCATTAGTGCTAAGTATCAGAAGACAATTGCTAAATATATTGCTAAGTTTCGTACCCCCAAGATAGAGAAGCAATATAGGGAAATTGGTGGGGGCTCCCCAATCCGGAAATGGTCTGAGTATCAAGCCACTGAGGTCTGTAAAATCTTAGATAAAACCTGTCCAGAAACGGCGCCTCATAAGCCTTACGTGGCGTTTCGTTACGCAAAGCCGCTAACCGCAGAAACTTATAAACAAATGCTAAAAGATGGCGTGAAGAAGGCAGTGGCCTTTTCTCAATATCCTCATTTCTCTTATTCCACTACCGGGTCATCCATCAACGAATTGTGGAGACAGATTAAGGCATTGGACTCCGAGAGATCTATATCTTGGTCGGTTATTGATCGTTGGCCTACAAATGAAGGTCTAATCAAGGCCTTCTCCGAAAATATCACCAAAAAACTACAAGAGTTTCCGCAACCTGTCAGAGACAAGGTTGTTTTATTGTTTTCCGCACATTCTCTACCCATGGATGTTGTTAACACCGGTGATGCCTACCCAGCTGAGGTAGCTGCGACGGTTTACAACATCATGCAAAAATTAAAGTTTAAAAACCCTTATAGGTTGGTTTGGCAATCCCAAGTTGGACCAAAACCATGGTTGGGAGCGCAGACAGCTGAAATTGCGGAATTTTTAGGCCCCAAAGTTGATGGCCTAATGTTTATTCCTATCGCCTTTACCTCTGATCATATTGAAACATTGCATGAAATTGACTTAGGCGTCATTGGGGAATCGGAATATAAGGATAAATTTAAGAGATGCGAATCTTTAAATGGCAACCAGACCTTTATTGAAGGCATGGCAGATCTCGTCAAAAGCCACTTACAGAGTAACCAACTCTATTCTAATCAACTACCTCTTGATTTTGCACTTGGCAAGTCCAATGATCCTGTAAAGGACCTTTCATTGGTATTTGGCAATCACGAATCTACTTGAAGATAAGACAGGGGAGAGGGATTCTTCTTCTTACCGACTCAACGCACCACACACCCTCTTTCCCAATTCTAATCATTCGATCTATGTAAACTTATCGTAACTAAACTAACTAACTACCAAGATTCTTCTTTACTTTACAGAGGTGTATTTCTTTAGTTGTCACATACAAAAACGCACACACACACACACATTTTGTCCTGTCCAATGACAAAATGTGACAGGAAACCCGAACGTCGGAGGTTTTCGGGTGGCAGTCATGTCGCCCAGTACTACTCGTAGCAGTAGTACTACTACCATTACACAAGGGCCAAACGTCAATGCTTTGCCGCGG

[C4+C5+Down] module:P_FBA1_-*HEMA_Sty_*-T_FBA1_, P_HXT4_-*HEML_Eco_*-T_HXT4_, P_OM45_-*GLTX_Km_*-T_OM45_, P_ADH1_-*HEM1_Km_*-T_ADH1_, P_PDC1_-*HEM2_Km_*-T_PDC1_, P_PGK1_-*HEM3_Sc_*-T_PGK1_,P_ENO2_-*HEM4_Km_*-T_ENO2_, P_INU1_- *HEM12_Sc_* -T_INU1_ , P_AFT1_-*HEM13_Km_*-T_AFT1_, P_TEF_-*HEM14_Sc_* -T_TEF_, P_OM45_-*HEM15_Sc_*-T_OM45_

GAGCTCACAAGGGCCAAACGTCAATGCTTTGACGTCTCCCGGAGGTATCCCGTTCCAGTGCGTGAGGGGCGTGGAGCGACAAACACACTCCCACATACATCTTTCCCGGAGGCAGAAACAAAATAAGAACAACAACAACACACCAACACGCCAACCATAGTAACCCACACGCTTAAACAACCAGTATCAGACCGGTACCCAACTTCTACGGCCGGCGGCGGCTGGCAGCGGCCTCGAGCATCCTCTATTCTCTCACCTCTTCTCTCGCTGTTGTACAACTTACAGGAATTCCCCTCTCTCCTCGAACTGGAAGCCGTGTTTCACGTGAGTGGTGGTACGATACCCGGTGGGAACCCTGTGTTGTGATATTTCGCCTTCTGGTTTTTGTTTTTGTCTCTCCCCTCTGGCTTCCAATACCGGAAAATTTGAAATTTTGGAAAAACAGGAAATCACAAAAAAACAGGAGAACCTGGAAAACGTAACGAAACCAAATAGTGGAACCCAACTGAATCCGCCGTAGACCAGATCAACCCACATGATTAAACGTCGCCCTCGACTTCCAGTGGTACTTCCAGTACCAATTCCAGTACCACTTTGAACAACTCTCCATAACACTTCCAATTCCACTTACAGTGTATGTGTGTGTATGTGTGTGCGTGTGTGGACAGGTTTCTTGCGCTTCTTGTACTTCTGGTGATGATAGATGGTGGATTCTCGGTACAGGAATTGGCTCAGGCTTTTTTTTTTTTCACATCGATTTCAGTTTGGGCTTTCCCTATGCATTCATTATATTGTGATTGCATTGAGCTTTGGAAATTTTTCATACTCTTCGAAATCATATATAATAAGTTACAATGTGAGTTATTCTCTCCTTCCTGGTTGTTGGTGTAAGCATCATTTAACTAGTCCCTTTTTTTTAAAGTTATTTAATATATATTTTTATTTTGTCCCAAACACCAAGAAGTAATCTTTACTTTTAGCACACAAATAACCAATAAATTCTAAAATGACCAAGAAGCTTTTAGCGCTCGGTATTAACCATAAAACGGCACCTGTATCGCTGCGAGAACGCGTAACGTTTTCGCCGGACACGCTTGATCAGGCGCTGGACAGCCTGCTTGCGCAGCCAATGGTGCAGGGCGGGGTCGTGCTGTCAACCTGTAACCGTACAGAGCTGTATCTGAGCGTGGAAGAGCAGGATAACCTGCAAGAAGCGCTGATCCGCTGGTTATGCGATTACCATAACCTGAACGAGGACGATCTGCGCAACAGTCTGTACTGGCATCAGGACAATGACGCCGTCAGCCACCTGATGCGCGTCGCCAGCGGTCTGGATTCACTGGTGCTGGGCGAACCGCAAATCCTCGGTCAGGTGAAAAAAGCGTTTGCGGATTCGCAAAAAGGCCACCTTAACGCCAGCGCGCTGGAGCGAATGTTTCAGAAGTCTTTTTCCGTCGCTAAGCGAGTGCGGACTGAAACCGATATCGGCGCTAGCGCCGTCTCCGTCGCGTTTGCCGCCTGTACGCTCGCCCGCCAAATCTTTGAATCGCTCTCGACGGTCACCGTACTGTTAGTTGGCGCGGGCGAAACCATTGAACTGGTGGCGCGTCACCTGCGCGAGCATAAAGTACAAAAGATGATTATCGCCAACCGAACCCGCGAGCGCGCGCAAGCCCTGGCGGATGAGGTAGGCGCTGAGGTTATCTCGCTCAGCGATATCGACGCCCGTTTGCAGGATGCCGATATTATTATCAGTTCGACCGCCAGCCCGCTGCCGATTATCGGTAAAGGCATGGTGGAGCGCGCATTAAAAAGCCGTCGCAACCAGCCGATGCTGCTGGTGGATATTGCCGTACCGCGCGACGTTGAACCGGAAGTCGGCAAACTGGCGAACGCTTATCTTTATAGCGTCGATGATTTACAGAGCATCATTTCGCATAATCTGGCGCAGCGTCAGGCTGCGGCAGTAGAAGCGGAAACGATTGTTGAGCAGGAAGCCAGCGAGTTTATGGCCTGGCTACGCGCCCAGGGGGCCAGCGAGACCATTCGGGAATACCGTAGTCAGTCGGAGCAGATTCGTGACGAACTGACTACCAAAGCGCTGTCGGCCCTTCAACAGGGCGGTGATGCGCAAGCCATCTTGCAGGATCTGGCATGGAAACTGACCAACCGCCTGATTCATGCGCCAACGAAATCACTTCAACAGGCTGCCCGTGACGGGGATGACGAACGCCTGAATATTCTGCGCGACAGCCTCGGGCTGGAGTAGGTGTTTCCTCCTTTGGAATGACGCGCTTACTTTACGAAAGTATTAATTTATATATAACAAATTTTATATACTAGAAACGCATGAAATGAATTATGTGAATGGTCTCTTTATTATACCGAAATTGGGCCGCTACCAGCTGTTGCTCCCCTTGCCTATTAATTTATTGGTTTGGTTTGGAAACGGTTGGTGAATTGATTGATTTGTTCAAAACTTTCCCAAAGTGACAGTCACGTGCCGCATATCAGTCGTGGAGAAGTTCTTATAAGCGTCCAACCAGCCAAATAGTCAATTTTAAGTTTGTATTTTTCTGGGGAAAATTCGGATGGGGTGATGAATTCGAGGTTTCTCCACATTGTGTTGTTGTTTTGGTGCCGAAGATGGTAATTACAGCAAAATTAAAAAAAAAAAATGCATTAGATAAAATTTATTATTTTTGTTTGAGCTTTTTTAAGGCACAGATTCTGCCGCACAGGGCGCACACAGGGCGCGCCCGCGCTTACGGTAATAGCCCTGGAAAAGTCCAGGAATAGCCACGGATGGAGAAAAAGAAACAACGGAAAATCTTTGGGGGTGGTTTTAGTAGGCGGTGCACGGGTGGGATCATGCGTTTTTGTCGAGCGATGCCGGAAGAATCTGGGGAAAGCAGAGTTCCCGGCGTGAGATATGGAGTGGAAGCGCGCGTAGACGCGGGCGCAGTGAAGAAAGAGGGAGAGCAAGTGCTGCAGTAAAATAGGAAGTAGCAGGTAACAAGATCTGAGCTGGATTGGTTAGGGTAGTTGTTCCGATACGGGGGTGTCTGCGGGCGAAAAAAAAAACTGGGACCATTACATTGTCCAACAATAGTCACATCACACTATGAAATTTTTTTAACTAATTAGTGTATTGTTCCGGAAATAGTGTGTATGTGTGAGAGTATTTTCGCTTGGCTTACTTGGCTACTTGGCACCGGCTGCTAGATTCTGGTGCCAAGGTGGTGCCAAGGTGTCCAAGAGAAGAAGCGTTAGCAGACGCTAAAGGTGAACGGGTTTTCGCCATGAATTTTCTGATATAAAAGGAAGTGGTTTGGAACTGAAATTTGAAATTGGAATCGTATCTCAATCTATCTCTCTCTGTTATATTTATTATTATTATCTTTTGAATGCTCAGACCCCATCAAACAATAAGAATCACACACACTTAATATTATTATTACTATTAATTTTTTGCGACCACTATTAAAAGTGTCATTATAGTGAAACTGTTATAGGAATAAGAAGACTATAATATTTCCAAACAAAAAAACTCAAAAAATTGATTAAAAATGAGTAAGTCTGAAAATCTTTACAGCGCAGCGCGCGAGCTGATCCCTGGCGGTGTGAACTCCCCTGTTCGCGCCTTTACTGGCGTGGGCGGCACTCCACTGTTTATCGAAAAAGCGGACGGCGCTTATCTGTACGATGTTGATGGCAAAGCCTATATCGATTATGTCGGTTCCTGGGGGCCGATGGTGCTGGGCCATAACCATCCGGCAATCCGCAATGCCGTGATTGAAGCCGCCGAGCGTGGTTTAAGCTTTGGTGCACCAACCGAAATGGAAGTGAAAATGGCGCAACTGGTGACCGAACTGGTCCCGACCATGGATATGGTGCGCATGGTGAACTCCGGCACTGAAGCGACCATGAGCGCCATCCGCCTGGCCCGTGGTTTTACCGGTCGCGACAAAATTATTAAATTTGAAGGGTGTTACCATGGTCACGCTGACTGCCTGCTGGTGAAAGCCGGTTCTGGCGCACTCACGTTAGGCCAGCCAAACTCGCCGGGCGTTCCGGCAGATTTCGCCAAATATACCTTAACCTGTACTTATAATGATCTGGCTTCTGTACGCGCCGCATTTGAGCAATACCCGCAAGAGATTGCCTGTATTATCGTCGAGCCGGTGGCAGGCAATATGAACTGTGTTCCGCCGCTGCCAGAGTTCCTGCCAGGTCTGCGCGCGCTGTGCGACGAATTTGGCGCGTTGCTGATCATCGATGAAGTGATGACCGGTTTCCGCGTAGCGCTAGCTGGCGCACAGGATTATTACGGCGTAGTGCCAGATTTAACCTGCCTCGGCAAAATCATCGGCGGTGGAATGCCGGTAGGCGCATTCGGTGGTCGTCGTGATGTAATGGATGCGCTGGCCCCGACGGGTCCGGTCTATCAGGCGGGTACGCTTTCCGGTAACCCGATTGCGATGGCAGCGGGTTTCGCCTGTCTGAATGAAGTCGCGCAGCCGGGCGTTCACGAAACGCTGGATGAGCTGACAACACGTCTGGCAGAAGGTCTGCTGGAAGCGGCAGAAGAAGCCGGAATTCCGCTGGTCGTTAACCACGTTGGCGGCATGTTCGGTATTTTCTTTACCGACGCCGAGTCCGTGACGTGCTATCAGGATGTGATGGCCTGTGACGTGGAACGCTTTAAGCGTTTCTTCCATATGATGCTGGACGAAGGTGTTTACCTGGCACCGTCAGCGTTTGAAGCGGGCTTTATGTCCGTGGCGCACAGCATGGAAGATATCAATAACACCATCGATGCTGCACGTCGGGTGTTTGCGAAGTTGTGATTTTTCCAACATGCTTAATTCTGTTCTAACTCGTAACTTGTTATTGACTTTTTTCTCTTAACCAAATTAACACATTCATTTCAGAAGATATGCCTTACTGGGATGCTTCATGTCAGTGCTCCTTAGCTTTCTTTTGCTGGAACACGAACGAATAATAATGATAATATCTTTTTATCGTCCTAATATTAATGCTAATTGCAATAAAACTAATGAATGATAATGAACGACTTTTATAAACTGAACATACATTTTATAATATAGACTCTAAAATATATTTCAGTTACTTTCAAGCCACACGAATTTTAGTTTGTTTAGAGATGGACCATGGACCATGGACGGTTAATGGTTTATATACAGCATGCACCCTTTGCCTTAAAACGGTAAAATGAGATAATGCCTTTCCGTTGTATCCCAGCCAGGCCAACCTCCCAGAGTATGTGCGGAGGGTAACGTCACGTAATGTAGAAAGTATTTCTGTATAGGTAGGGTACCCACCCGCATATGAAATTACTCTGTAGTTCCTGGGTCCCGTCGCCTCCGATATTCTTCTGCTTGATTTCTTTTCTGCCGTTTGCTAGTTTTCTTCAACCCCGGTTACGTTTATGCTTTTTCCTTTTGCTCTAGAAGACGGGTTTTTTTGCTCTTAACAAAGGCCAATCAATGCGATGGACTGGTCAATCTCGGCCGGACAGAGACAAACCCATCGTTCTTGACGATTGGGTTACAGTTCGCAGAGAGAAATGTAGTGGAATGGAAAGAGATCGCAGCAGTATATAGTCAGTTTATTCAGGTTACGCTATGGCATTTTTTTTTTTTGTTACGTAACTTGAGTACGGTGTTAGGTTTCTCTTTCTTTAGAAATTAGGGATGCTTCGGACAATTGGGGTTTTAGAAGAAGGGATACAAAGGAGTAGCATGCGTCGGGTGGTAGAGCTAATGTTAGCTGGTTGGGCTGGCCTGCCGGCTAGAATGGTTAATTGGTAGACCAGGTTTTTTTTTTTTCCCTTACTTCATTCCCTTACGTCTTTTGGGGCGACACAGGGTTTTGTATGAAGAGAATTGCATTTGCTATTAGTTAAGGCTTCTCCGAACCTGTATAAAAGTGGAGAGTTCGCAAGGTATGTAATTATATATACAGGATATATTTCCCTGCAATGCTAGTATTTAATGATGATAGACAGTTGGTTTTACAGGACAAAGATTCGTTTGGTCCATTGATTGAATAGCACACACTTCTGACTACGTAGTTGTTTAACAAGATCGAAGTGAATCTGACTCTAAGAATACAGTATACGAAACAATGTCTGTTTCGCTAGTTATTGGTGCTAAGGCACCAGTTGTCGCTTATCCAGAGTTGATTGCAGCTCGTTTGGTTAATGCTTCTCATCAAAACGCCATTGATATCTCTTTTGTTGAGGATAAGAAAGCCCCAGCTGCCTCTTTCCAAGGCAAAACTGAAAATGTTTTAGCCGAGATTGCTGCTGCATATCCAGAAGTGTTGAAGAATGTGGCTGGTTTCGAAGAATGGGTCGAATTTGGTGCCAACCAGCTTGTAATTAAGAACTTCCAACAACTTGCCTCATCACTTGAAAAGTTGGACGCCCACTTAAATTTGAGAACCTACATTTTGAACACTGTTGAACTAACTTTGGCTGATATTGCTGTCTGGGGTTACTTGAGATCAAACGGTATGGTGGGCTCTATCATCAAGAATAAAGTCTACATCAATGTTTCTCGTTGGTACTCAACCTTGGAATCCATCCCAGAGTTTGGCCAAGCTCATGAATTTTTGACCAAATCTTTGCAAGAAATGAAGAAAGCTGCTAATGTAAACAAGAAGAAAGAAACACACAAGGCCAACTTTGAAATCGATTTACCTGATGCCAAGATTGGCGAAGTCGTTACGCGTTTCCCACCTGAACCATCTGGATACCTACACATCGGACATGCCAAGGCTGCTTTGTTGAATCAATACTTCGCTCAAGCTTACAAAGGTAAATTGATTATTAGATTTGATGACACAAATCCTTCGAAAGAAAAGGAAGAATTCCAGGACTCTATTTTAGAGGATTTGGAATTATTGGGCATCAAGGGTGACAGAATTACTTACTCTTCTGACTACTTCCAAGAAATGTATGATTACTGTGTTCAAATGATCAAGGATGGTAAGGCATATTGTGATGATACTCCAACCGAAAGAATGAGAGAGGAACGTTCTGAAGGTATTCCATCTTGCAGAAGAGAAAGATCAGTTGAAGAAAACTTGAAGATTTTCACGGAAGAAATGAAGAACGGAACCGAAGAAGGCTTGAAGAACTGTGTTCGTGCTAAGATCGACTATCAAGCTTTAAACAAGGCTCTAAGAGATCCAGTTATTTACAGATGCAACTTGACTCCTCACCATAGAACTGGAACTGCTTGGAAGATTTATCCAACTTATGATTTCTGTGTTCCTATTGTCGACTCTTTGGAAGGTGTTACCCATGCATTGCGTACAATTGAGTACAGAGACCGTAACCCACAATACGAGTGGATGCTAAACGCTCTTAACCTAAGAAAGGTCCATATCTGGGATTTCGCTCGTGTTAACTTCGTCAGAACCTTGCTATCAAAGAGAAAGTTGCAATGGTTGGTTGACAAGGATATTGTTTCCAACTGGGACGATCCAAGATTCCCAACTGTAAGAGGTGTAAGAAGAAGAGGTATGACTATTGAGGGCCTAAGAAACTTTGTTTTGTCTCAAGGTCCATCTAGAAATGTTATCAATTTAGAATGGAACCTCATTTGGTCTTTTAACAAGAAGGTTATTGACCCAATCGCTCCAAGACACACTGCTATTGTTTCACCAGTTAAGTTGCACTTGGAAGGTAGCGAAGTTCCTCAAACTCCAAAAATTGAAATGAAATTGAAACATAAGAAGAATCCAGATGTTGGTGAAAAGAAGGTTATATATTACAAGGATATTCTCATCGACGAAGAAGACGCTAAGTTGCTTTCTGAAGGGGAAGAAGTTACTTTGATGGACTGGGGTAATGCTATTATTACAAAGAAAAATGAAGATGGATCTTTGGTTGCAAAGCTACACTTGGAAGGTGATTTCAAAAAGACCAAATTTAAGTTAACCTGGTTAGCCGATACCGATGATAAGGTCGAAGCTGATCTTGTCGACTTTGACCACTTGATTTCCAAAGACAAGTTGGAAGAAGGTGACAACTTTGAGGATTTCTTGACTCCAAAGACCGAATTTCACACCAGGGCTATTGCCGACTTGAATGTCAAGGATATGAAGGTTGGTGATATTATTCAATTTGAAAGAAAGGGTTACTACAGATTGGATTCCTTACCAAAGGATGGAAAGCCTTACGTATTCTTCACCATTCCAGATGGTAAGTCTGTTAACAAATATGGTGCTAAGAAATAAAGATAAGACAGGGGAGAGGGATTCTTCTTCTTACCGACTCAACGCACCACACACCCTCTTTCCCAATTCTAATCATTCGATCTATGTAAACTTATCGTAACTAAACTAACTAACTACCAAGATTCTTCTTTACTTTACAGAGGTGTATTTCTTTAGTTGTCACATACAAAAACGCACACACACACACACATTTTGTCCTGTCCAATGACAAAATGTGACAGGAAACCCGAACGTCGGAGGTTTTCGGGTGGCAGTCATGTCGCCCAGTACTACTCGTAGCAGTAGTACTACTACCATTACGaagaaatgatggtaaatgaaataggaaatcaaggagcatgaaggcaaaagacaaatataagggtcgaacgaaaaataaagtgaaaagtgttgatatgatgtatttggctttgcggcgccgaaaaaacgagtttacgcaattgcacaatcatgctgactctgtggcggacccgcgctcttgccggcccggcgataacgctgggcgtgaggctgtgcccggcggagttttttgcgcctgcattttccaaggtttaccctgcgctaaggggcgagattggagaagcaataagaatgccggttggggttgcgatgatgacgaccacgacaactggtgtcattatttaagttgccgaaagaacctgagtgcatttgcaacatgagtatactagaagaatgagccaagacttgcgagacgcgagtttgccggtggtgcgaacaatagagcgaccatgaccttgaaggtgagacgcgcataaccgctagagtactttgaagaggaaacagcaatagggttgctaccagtataaatagacaggtacatacaacactggaaatggttgtctgtttgagtacgctttcaattcatttgggtgtgcactttattatgttacaatatggaagggaactttacacttctcctatgcacatatattaattaaagtccaatgctagtagagaaggggggtaacacccctccgcgctcttttccgatttttttctaaaccgtggaatatttcggatatccttttgttgtttccgggtgtacaatatggacttcctcttttctggcaaccaaacccatacatcgggattcctataataccttcgttggtctccctaacatgtaggtggcggaggggagatatacaatagaacagataccagacaagacataatgggctaaacaagactacaccaattacactgcctcattgatggtggtacataacgaactaatactgtagccctagacttgatagccatcatcatatcgaagtttcactaccctttttccatttgccatctattgaagtaataataggcgcatgcaacttcttttctttttttttcttttctctctcccccgttgttgtctcaccatatccgcaatgacaaaaaaaatgatggaagacactaaaggaaaaaattaacgacaaagacagcaccaacagatgtcgttgttccagagctgatgaggggtatctcgaagcacacgaaactttttccttccttcattcacgcacactactctctaatgagcaacggtatacggccttccttccagttacttgaatttgaaataaaaaaaagtttgctgtcttgctatcaagtataaatagacctgcaattattaatcttttgtttcctcgtcattgttctcgttccctttcttccttgtttctttttctgcacaatatttcaagctataccaagcatacaatcaactATGGAGTCTGTTGTTCGTCAATCTGCAAGGGTTTGTCCCTTCATGAAGTCTGCTACAGGGTCTGTGCAGAATGTGAAGGCTTTGAAAAACGCCAACTTGCCAGCTATTGCGCAAAAGTGTCCGTTTATGGGTCGTGCTATGGAGCAGCGTAGAGCATATGCGTCAGCATCCGGGTCAGGAGCTGGGTCAGGAGCTGGTGCAGCAGCAGCGGCTTCTCCAGCAGCCGTTGAAGCGAGGAACGCAAGTACTGCTTCTGCAGATGCTACTGTTTTGGACCATGCTACCAATGAAGCTTCCTTTGACTACCAAGGATTATTTGAGTCTGAGTTGGCCAGAAAGAGAATGGACAAGTCGTATCGTTACTTCAACAACATCAACCGTTTGGCAAAGGAATTCCCAATGGCCCACAGACAACAAGAGGACGATAAGGTCACTGTGTGGTGCTCCAACGATTACTTGGCTCTTTCCAAGAACCAAGAAGTCATCGATGTGATGAAGAAAACTTTGGACAAGTACGGTGCAGGTGCTGGTGGGACCAGAAATATCGCAGGTCACAACAAGCACGCGTTGCAGCTAGAAGCAGAAATCGCAGCCTTGCACAAAAAAGAGGGTGCGTTGGTGTTCTCCTCTTGTTTTGTCGCTAACGATGCCGTCATCTCGTTGCTCGGCCAGAAGATTAAGGATCTAGTCATCTTTTCCGATGAGCTGAACCATGCCTCCATGATCGTGGGTATTAAACACGCCTACACCAAGAAGCATATCTTCAAGCACAATAACTTGGAAGAATTGGAAAAATTGCTAGCCATGTACCCAAAGTCCACTCCGAAATTAATCGCCTTTGAATCTGTTTACTCTATGTCTGGGTCCGTCTCTGATATCAATAAAATTTGCGACTTGGCAGAAAAATACGGTGCCTTGACTTTCTTGGATGAAGTTCACGCAGTTGGTTTGTACGGTCCACATGGTGCCGGTGTTGCAGAGCATTGTAACTTTGAAGCTCACCGCAAAGCTGGTATTGCATCCCCAGAATTCCGCACTGTCATGGACCGTGTCGACATGATCACCGGTACCTTGGGTAAATCTTTCGGTACTGTCGGTGGTTACGTTGCTGCTTCCTTAAAATTAATCGATTGGCTCAGATCTTACGCACCAGGATTCATCTTTACCACATCTCTACCACCATCCGTTATGGCGGGTGCCGCAGAGGCTATCAGATACCAACGTTCTCACTTGGACCTAAGACAAGACCAACAAAAACATACAACTTACGTCAAGGAGGGATTATCCGATCTGGGTATTCCAGTGATGCCAAACCCATCCCACATTGTCCCAGTTTTGGTTGGTAACCCTCATTTGGCTAAAGAGGCTTCGGATATCTTAATGCATAAACATCGTATCTACGTTCAAGCAATCAACTTCCCAACGGTTTCCAGAGGTACCGAACGTTTAAGAATTACTCCAACTCCTGGTCATACTAATGATTTGTCTGACATCTTGTTGGATGCTATGGAAGACGTTTGGTCAACCCTACAATTACCAAGAGTTCGTGACTGGGAAGCCCAGGGCGGCTTATTGGGTGTTGGTGATCCAAACCACATCCCTGAACCAAACTTGTGGACTGAAGAACAATTATCTTTGTCTAACGATGACTTGAACCCAAATGTCAAACATCCAATAATAGAACAATTGGAAGTTTCTTCTGGTATTAGATATTGAgcgaatttcttatgatttatgatttttattattaaataagttataaaaaaaataagtgtatacaaattttaaagtgactcttaggttttaaaacgaaaattcttattcttgagtaactctttcctgtaggtcaggttgctttctcaggtatagtatgaggtcgctcttattgaccacacctctaccggcaTGAATAATGAATGGCCTTGTATTCGTTTTTTTCCGAGAGAAAATTAACAAGAGCGAAAAAAAAAACGGGCTTCGGTGAAAATCGGGTGAATATGCAACTAGCGGGACGAATGCTCTGGAAATGCATATCCTATGCAACTAGCGGGATGAACAAATCTCACCCCAGAATTCGCAGGAAAAAACAGGAAAAAAAAAAAGAAGGCCACCACGGCCACAAAGACCACAAAGGCCACAAAAAGAACAAAAAAACAACCGTCCCAGCTTCCAGTGTTTGGAATACTGGAACACAGGAAGCCGCATAAGAGTGGGCGTTGCACAGGAAGCCAGGCCCAGAAGCCCCAGAGTTACTTTTTTTTTTTTGTTTTTTCCTTCTGTTCGCTGTGCCCGCATCAGATGATGCGCCTTTATTTACGATGCCAATGCGAATAGCACCAGTGAGAGCACCAGTAAAAGCATACGCATACACATACACACATAGAGCAAGCAAGCAGGCTAGCAACCAGGAAAGGCTGCCAGTGACTGCTACTGGGTGTCTAAGAACCGTAGGGCGGATTATTGTTGCGGTGGTTGGTTGCGGGTGGTTATGCGATGGTACGGTGCAGAATCGTACGGTGTTGGTTATGGAATTAGTATGGGTATGTGATATGTGGTAATATGTGATATTGGGTTATTGTGATTTGGAATACTGAATATCGAATATGGGATATGGAATATGGCCATGGCATGGTATGGTATGGGATGGGAGTATTCTATTTTATTTTATTTTATTCTGGTTCCTGCGTTTAGGGTAGGGTAGGAAGAAGGTGAGTGCTTTTGTATATAAGTGGAGTGTCTGGATCAGTTTTGTGGATTGTGAATGTTAGTTTCCCCTTTAATGTATATTTGTATTATTTGCTTTTGAGTACTCAATAACCAAGCACAACTACTAGTTTTAAAGGATCCATCCTCTTAAACAGTACAAATCGCAAAGAAAAGCTCCACACCCAAACCAAATAATTGCAATGGTTCACGTTGCAAAATTTTTAGACGAACGTCCTACTGAAATTCCTTCCATCCTTGCTGGTGGATACAACCACCCTCTATTGAGAGAATGGCAGAATGAAAGACAGTTGACTAAGAGCATGTTCATTTTCCCGCTATTCATTAGTGACCAGGACGAGGAAGAGACCCTGATACCATCGCTACCAAACATCAAGAGATTTGGTATCAACAAATTGAAGGATTATGTTGCTGGTTTGGTTGCCAAAGGTTTACGTTCGGTGATCTTGTTTGGTGTTCCATTGAAGCCTGGTGCTAAGGACGAGGTTGCAACCGCAGCTGACGATCCAGATGGGCCTGTCATCAAGGCTATCAAGTTGTTGCGTAAGGAATTCCCAGATTTGTACATTATCTGCGATGTGTGTTTGTGTGAATACACTAGTCATGGTCACTGTGGGATCTTGTATGAAGATGGTACTATCAACAGAGAAAAATCTGTGCAAAGAATTGCTGCTGTTGCTGTGAACTATGCGATAGCTGGTGCGCACTGTGTTGCCCCAAGTGATATGATTGATGGTAGAATCAGAGAGATCAAAATGGGATTGATCGAACACGGATTAGCCCACAAGACGTTTGTTATGAGTTACTCTGCCAAGTTTAGTGGTAATTTGTACGGTCCATTCAGAGATGCAGCATGCTCGCAACCAGGTCAAGGTGATCGTAAGTGCTACCAGTTGCCTCCAGGTGGTCGTGGTTTAGCCAGACGTGCATTGAAAAGAGATTTGGCTGAGGGTTCCGATGGTATTATCGTCAAGCCTTCGACCTTTTACTTGGACGTTATGTCCGATGCTGCTGAAATTTGTCGCGACATTCCAGTTTGCGCATACCACGTCAGTGGTGAATACGCAATGTTGCATGCTGCTGCTGAAAAGGGTGTTGTTGACTTGAAGAGCATTGCTTTTGAATCCCACTACGGTTTCTTGAGAGCTGGTGCTCGTTTAATCATCAGTTACCTCACCCCTGAGTTTTTGGAATGGTTAGATGAAGTGAACTAAAGAGGGAGAGGATAAAGAGATAAATTACGATTTTGGATTTTAATGATTTTATAAACAACAACAACCAACCAGCCTTTTACTTTATTTGGCATATACACAAGCTTACTCCATTTCATTGATTATCTATGTGTATATATATAAGTGATGTATAACAATTATTATTATACATAGATAATATTTTTATGATATGTTTTTTCTGAGTTTTGATATTATTTATTACAAGTTACAAGTTACAAGTTACAAGTTACCAGGAAGAATTAAATAAAGGTAAATTGGGGGAATTATAAGCGTATGGGCATAGCCTTGTCCAAGGCCTTGGCCTTGGTCAATGCCCTTATTCTCTTGTTTCCATTGCCTGTCGATATACGACTTCAAGGCCATGTACCTGAGATCCTCATCTGGGGCCTGAAGATCCATTGGTGAGTGCCACTGGCTGCTGTGCCGTTGTGAAAAGGCCCTGTGTAGCAGAGAGGGAGGTGTGTATAGGTATATATATGTATATATATGTATATAAGTGTGGATTTAACACACTCTCTCTTCTTTCCCATGCCATGCCAGTCACGTGTTCCACACGTGACTGTACTTACCCTCACATTACCCTCACTCTTTCACATTACCCTCCCCATCCCATCTTCTTTGGTTTTCCCCCCCAGCTTCCTGTCTTCCACTATCCCTGCAACCACCACCACCACACAACAAACATCCCCCAGCAGCCCCCCAGCAGCCCGGAAAAATCCACGCCTCCCCCCGAACACACGAAGCCCGCCCCGGCTTCCACTCCAACACTGCCAACACTGGAACCCCGCACCACGTAACCACCCACTTTAGTGGCTGCCCGCCCCTCCTGCGCTTCCCTGGGGCTTCCCTTGCGACCACACTCCGGTGGCATGGCCCTCGCTTGCTACATGCTACCTTTTTTTTGCCCTTTTCTGGCCTTTTCTGGCTTTTCTGGCCTCACCACAGTGTACCACAGTGTAGTAGTAGTGTAGCATTGTCGAGTGGTATTGTCGAATGGTATTGTAGCCTGTTGTAGCCTGTTGTAGCCTGGTGTAATGTCTGGCATAGTCTGGTGTCTGGTGTCTGGTGTCTTTTGGAAACTGAACAGTGGAAAGTGGAAGTGAAAAATTGTATAAATATAGGTGTCCCATTCGTGGTTTGGTGGATGTGTCCTTGACATTGCAGGTTTCTCTGTTTCGCTGTGTTAAGACTTGTTAACACTTGATAAGTATTTCCCTAGTAATACCCCACCAACCATCGGTTGATACATTTTAATACACACTACCTATATAAAGATACAAAAATGGGCCCTGAAACTCTACATATTGGTGGGAGAAAATCGAAATTGGCGGTAATACAATCCAACCATGTTTTAAAACTGATCGAAGAAAAGTATCCGGACTACGACTGCAAGGTTTTCACTTTGCAAACTCTTGGTGACCAGATTCAATTCAAACCTTTGTACTCATTTGGCGGTAAAGCTTTATGGACAAAGGAGTTGGAAGACCATCTTTACCATGACGATCCCTCAAAGAAGCTTGACTTGATCGTTCATTCTCTGAAGGACATGCCCACTTTACTACCAGAGGGTTTCGAGCTGGGGGGTATCACTAAGCGGGTCGATCCAACAGATTGTCTTGTCATGCCCTTTTACTCTGCTTATAAGTCTCTGGATGACCTTCCAGACGGGGGGATTGTGGGAACCTCATCCGTGAGAAGATCTGCTCAGCTAAAAAGAAAATACCCACATTTGAAATTTGAAAGTGTCAGAGGAAATATACAAACTAGATTACAAAAACTAGACGACCCAAAATCTCCGTACCAATGCATCATCTTGGCGTCTGCTGGGTTGATGCGTATGGGGTTGGAAAACAGAATTACGCAGCGATTCCATTCGGATACAATGTACCATGCAGTTGGACAAGGCGCCCTGGGTATAGAAATTAGAAAGGGTGACACCAAGATGATGAAGATTCTTGACGAAATTTGCGATCTAAATGCAACTATATGTTGCCTTTCGGAGCGTGCTTTGATGAGAACTTTAGAGGGGGGTTGTTCCGTTCCTATTGGTGTGGAATCTAAATACAATGAAGAGACTAAAAAATTACTATTAAAGGCCATTGTAGTTGACGTTGAAGGCACAGAAGCAGTAGAAGACGAAATTGAAATGCTAATAGAAAATGTTAAAGAAGATTCCATGGCGTGTGGTAAGATACTAGCTGAAAGAATGATTGCCGATGGCGCAAAGAAAATTCTGGATGAAATTAATTTAGACAGAATCAAATGAGTGCATTGTTGAACTTTCTAATGAAACCACACGTGAATTAAAGGAAAGCGCGCAGCAGCAGCAGCAGCAGCTTTTCTTTCCTTCTTCAAATCATACCTTTTCTCTATTTCAACGTTCTTTTCATTTCACCATGAAATGAGTTGAATTGTTGCTTTGAGTTTACGACGATACATAAATATTAATTAAAATACGAAAAATACACATTTTATTTACGACACTTGCCTCTCCCCCTAGATGTTACGCTGCTCAGGTTCGAGCCGCTCGATCCCTAGCATATCGAGAACTTCGTACTCGTCCCCCATTTTCAATTTCCCAAAAAAACAAGAAGCCGGAATCCACCGGAATCCACTAATACGCAAAAAAGACGACGCCAGCTTCGCCGTTGGGCATTGGCACCATATTCCATCGTTCGTTTGACTTTGGTTTTGGTTTTAGGTTCTGGCTTCGTGTTTTCCCCTCACTGCCCTTGTCCTTGCCGTGCTCCCCCTCGTTCTCTGCGCAAAGTCTGGTGACCCTCCGGCTGCCACAAAATCCCGAGGCACTGAAAGGGTGGGGCCTCGGGGGTCTCGGGGCAGAAGCCTTGAGCTTCGTGTTTATTCACTCTGTACGGGCGGTTCTAGCGTTTCTGGGAATCCAAAGAGGAAGGGCCGTGTTTCGTGCAGGCATTTTAGTGGATGCCCGGGATGTAAGCTCTTCGGTTACAGAAAATCAGGTTGTGATTCCAGTATGAGGAATAGTGGATGCCAGGTTGTGCGAGCATCACCAATATCATGGGAAATGCGAAATGGGCATGAGCCCAACCAACACAACACAATACCATACGCCAAATGGTAGCCTGAAAAAAAAAAAAAAGGTCTTGCCAGACCACTAAACTCGAGGTAGAACAAGACTGAGAAAGAGTGTGTGATCCCTTTGGTGGTAGTAATTTTTTTTTTTTTTTTTCAAGTTTCCAGCATCCCAAACCGAAACCCAACATCATCATGATCCCATCCCATGCCTATGCCCATGCCACATTCATTCATTGCACAACACACAACGTAGTGGACGACAGCATAGCATCAGTTAACTAACGGCCACTTGTTTTTTTTTCTTCCATTTTTTCGTTTTGTTCTAGCAACAATGAGTTCTAATTTTTTTGTTATAAAAGGGACAGTTAAGGTATATGTAAGGTTTCTTGTTCCATTTGGGTTAGAGTTTTGTAGTATTAGTTTTGGTTCTTAGTTCTTTTGGCCTAAGAAACCAGTAAGAATTCTTCTGATTTTCTTTACAAACACATCAACAACAACAAACACAAACTACAATGTCCAGAATAATTTTTCTTAAGAACAAAACAACACCTACAGACCCGTACGAAACCACTTTTACAGATGGAGGGTTTGAACCTGTATTTGTTCCACTAATACGCCATTTCCATGTTCCAGACGAAGCGTTGACGCTATTCAGAAACAGGAGCTACCTAACGAAATTGAAATACATTATAGTTACTTCACAACGAACAGTCGAATGTCTAAATGAGTCAATACTTCCTAAAATGACTACGGAAGAGCAATCTCTTTTAAAGGAAAAGACTATATACACAGTTGGACCGGCCACTTCGGAGTTCTTGAGAAATTCTGGTTTTCAAAACATAAGAGGAGGAGTTGAAGTTGGAAATGGCGGATTACTTGCTGATCTGATTGTTAAATCGCATTCTGAGGAAGAGATTGATCACTTTTTGTTTTTAGTTGGAGAAATAAGAAGAGATATCATTCCCAAGAAACTTAAAGCTAATGGCTACAAGGTCAACGAAATTGTAACTTACAAAACCGAGAATCTGTTAGACAATATTGATCGTTTTATTTTGCACTATAAGAATGATGAGAATAACCAAGCGGTTATGAACAAGCCCTCCACATGGATTGTGTTCTTTAGTCCCCAGGGTACGGAAGATATAATTGAGTACCTAAAAGAGAACAAGGGCTATAAAATTGCTAGCATAGGTCCTACAACGGAAAAGTACCTCCTAGAAAAAGGTTTGAAACCAGACACTGTTAGTTTTAAGCCGTCTCCAATATTCTTACTGAATGCCATTAATAGTTACAAATAAATCTAGCTGACGCGACGCTTTTTTTCTCCAACTAAGGGAATCCAGGGAAACCCCCCTCGAGCCCCAGTTTTGGTTAGAAATTGTCCTTTTTTATACTATTGAATGACCTAACTAAATCTATATAAACCTTTTTAACGTCTAACGACTCTTTATTATTGAAGTGGGAAGTCTTGGGATGGGAAGTCTTGGGATATTTCAGGCCTTTGGCCAGGCCAAAGGCCAGGCCTTGGCCCAAGGTACCACCCCCTTATATATGCTCAGCCAATTGACTCAGCTGTCCCTCCACACTTTATTCCTGTAATCCGAAAAGGTAAACAGACACAAAAACGACAAGAGAAGCAAACACAAAAAAAAAAAAAAAAAAAAACAAAAAAAAAAAACACAAACACAAACACAAACACAAAAACGCTAAATTATGCACACAAGGGCCGGCGGGGCTGCCGGAAAAAAAAAGGGAAAAATACACAGACGAGCGCGCACAGATGGGGTTACCACTGCAAGTTACAAGTTGCAAGTTGCACGCTGGAATCAGAATTGGAATCAGAATTGGAATTGGAATTAGAATTAGAATTAAACTTGGGGTAGCCACGGGAACGGGATAACTCAGGAATCGCTCGCAGGCGTCTCCGTCTAGGCAATCCCAAGGTAAGCCTAGGCACTCCCACAGGGGAAAGAACGGTTGAAGGCAAAGTAGTGCTAACAATTGGTAACGAATGGTAACAAGTGTGTCCGTCTCCACCTGACATTTGCTAGAGCTGGGGATTCCACATTCTTGTGCTCTGAATTCTCAAACCGAAATGGGGCGTTGTTACCCCAGGTATCCGGTTGTAGTTGGCACTGGGGATGGAAAAAAATGATGTTGATGTTGAGTTAGTTGGGTTGAGTCAATTAGTGCGTGAAAGTATCACCACTTTTGTCATCCGGCGTTTCTGTGCGAATCACACACACACACACAGTTTATTGGAGCACTTGTTTCTGGCGTATTCGTAATTGTTCTGCGGTGCGGTTCTGTGTGCATTTTTCCTGGGGTGTCTGCCGCACCTACTCATCACCCACGCCGTGGGTTTGAGCCATGGCGGAGGTACGACTGACTGGCTGCCTGCCTGCCTGACTGACTGCCTGACTGCAGGAAAAGAGGGTTTCGAAGGAAAAACTTTTCCTGTGTTAATCCGGCCGTGCGCCGCTGCTCCAAAATCCACCTTCATGAGAAGGAGTTTGAAAAAACAAAAAAATTCACATATAAAAAGCGTATCTCGAGATCTCAAAGTCTCCCTTGAATCGTGTTTGCCAGTTGTAACTCATCCTTTATTCTTCTATTCTATCTCTCTCTTTCCTTCCCCTAATCAGCAATTAAATCCGGGGTAAGGAAGAATTACTACTGTGTGTAACGGTTATATTTCGTTTTTTATTTTTTTTTTCCATTGCCATAGAGAAAGAAAAAAAAAAAAAAGAGAGTTTGTGAAGATCTTCCATTCGAATCCCATAAGTGACACATTTAATTTTTTTTTTGTTAGATATGGGTAACTTTCCAGCTCCAAAAAACGATTTGATATTGAGAGCCGCAAAGGGTGAAAAAGTCGAGAGACCGCCATGCTGGATAATGCGCCAGGCAGGTCGTTACCTGCCGGAATATCACGAGGTGAAAAACAATCGTGATTTCTTTCAAACTTGCAGGGATGCGGAAATTGCTTCTGAGATTACTATCCAGCCGGTAAGACGCTATAGAGGCCTCATTGATGCTGCTATTATTTTTAGTGATATCTTAGTTATTCCGCAAGCCATGGGTATGAGGGTCGAGATGCTCGAAGGTAAAGGTCCACATTTCCCAGAACCTTTAAGAAATCCGGAAGACCTCCAAACGGTATTAGACTACAAGGTTGATGTTTTGAAAGAGTTAGATTGGGCTTTCAAGGCAATCACCATGACAAGGATCAAGTTGGATGGTGAGGTTCCCTTATTTGGCTTTTGCGGGGGACCTTGGACTCTAATGGTTTATATGACGGAAGGCGGTGGATCCCGTCTTTTCAGATTTGCCAAACAATGGATTAACATGTATCCAGAGCTTTCTCACAAATTATTACAAAAAATCACTGATGTGGCCGTGGAGTTTCTGAGTCAGCAAGTCGTGGCGGGTGCTCAAATACTACAAGTTTTTGAAAGTTGGGGTGGTGAGCTTTCGTCTGTAGATTTTGATGAGTTTTCCCTACCATATTTAAGACAAATTGCCGAAAGAGTGCCTAAAAGATTGCAAGAATTAGGTATCATGGAACAGATTCCTATGATCGTTTTTGCGAAAGGGTCGTGGTATGCTTTGGACAAGCTATGCTGTTCAGGATTTGACGTTGTTTCGTTGGACTGGTCCTGGGACCCAAGAGAAGCGGTAAAAATAAACAAGAACCGTGTCACCTTGCAGGGCAACCTGGATCCTGGCGTCATGTATGGTTCTAAAGAGGTAATAACAAAGAAAGTTAAACAGATGATTGAGGCTTTTGGAGGTGGGAAGTCCCGCTACATTGTTAATTTCGGTCACGGTACCCACCCTTTCATGGATCCAGACGTCATCAAGTTTTTCTTGGAGGAGTGCCACAGAATTGGTTCGAAGTAAGGCCGCAAGCTTTGATCTGATCTGCTTACTTTACTAACGACAAAAAAAAATCAAAAAAAAAAAAACAATCAGTCCTTCTCTTCTTACGATATGATATGATTAAATGATGCTATGAAATCATCTTCTTCTTAACTTTCTTAAATCTTACGCGTCACTTACTCTATATACCCGTTTAGCTTTGCCTGGTCACAGCGACATTTTATATAAGTGTACGTATTTTCTTTTTTTTTTTAAAAATTTCTATTCTAACCTTAGAAAAGTGCCCTTTAAACCAGCTGTCCTGGCACTATATCTTTATCATGTGCCGGTCGCTTTCCCTTTCCGTTTCCCTTTTCCTTTCAATTGGTGGCCTGGAATTCCGAACTCATTTTCGCATCTGAAACTAATTCTCGAAACCTTTAACATCAAACAATTGAAAAGATCATCATCACCAGAAATAAGAAAAAGATCAACACAACAGCTAATAACAGTACGAAAGAAAGATCGCTCGAGTGAAAAGGCAGCCAAGAAAGGTCATTCGATTTGGGTCTAGACTGATTATAGACATACCAATTGCACTCAGTAAGAAAATGAGTTTCAAATTTGACGATGACGGTGTGGTAAAAGAATTTCACGGCAACACCATCATATGCCATATTCCTCAACAAACCGAATTCTTCAACAAATTGTTGGACTTCTACCGTTTTGCGAAACGACTTTCCTTCTACGACAAGATCACCCTACTTCCTCCTTCAAGCTACCACGTTACGATCATGAATTGCTGCCACGAACACGATCGTTCTGAGGGCCACTGGCCCAAAGGAATCGATCCGGACACAAGCATGCTGCGGTGTACATCACATCTGACCAACATTCTAGGATCGGTGCCAGGTGAATGTACGTAATGGAGTTTGGATAGATTCGATACAGTTCATTACCAAGAATGGACAGCAGTCCCCCATATACGGGAATGCAACCGGAGGATCATTGAACCAGATTAGCATTGTCGCTCCATACAACCTTAAAGGGTTCTTTGGTAATTGTTCGAATTGGATGGACAGTCTTGGAGTGTTGTACGGGTAATTGGAGCCTGTTTGAAGCGTTTGTTTCCCTTTGGAGCGGTTTCCGTGTCATCAAGATGTCGGGGTCCGTCAAAAAAAATTTAGCGGGCACTCCGGGGTCCGTGGGGTCCGTGTGGTAGGGTGTGCCAGACCCGGTACTCTAAATCCATCCCAAAGAGCAAAGAAAGGGTTGCATGAATAATGGACCCTTGTTGCGAGCAGTTACTGTTGCAAGCAGTTACTGTTGATAGAGATGGTGTTCGCTAGTATACAGGTCAAATACTTAAGGTTATCCTTTGTGTGGGAGGATTTTAGATTACCGAGTCTTTTTTTAGTGGAGTGCAGTGGGATGAGAAAAGTTGGCCCGACCTCGGAGAATTTCTCGTAATATGTTGCTCAATCTATTAAAAAAAAACTCGGATACTTCGAAAGCACCCCGTGTGGTTATCCCATGCGCGTAAAATTCACCCTCTGCGAATCGAGGGCGCCCCTTCAGAAACGGGATAACCGGGTGTTTTAGCCCAGGGCAATATCCAATAGGGCTAACCTAGTCGCTTTCCGAGCCCAATTTGTTAGTCTTGGCGCACCCCGATTGCTTGACACGGAGTCCGTCAATACCTATAAAATTGGGATTTAGAGGTTAATTATTTTTTAGGAATCTGTAACCACACCCCTCTTCGGACCCCGCCTAGTATTTTCTTCTCATGCCCGAAATAAATAAAAGGACAATTCTCCCCCAGAATATTACTACTTGTTCATTGTTTTAAAATTCCAACCAGGCTTCGTTACTCCTAAAAAATTTTCGAACCACGTACACCTTCAGCAATGACAGACTCTGCTGTGCCAATGAGAAAGAAGATGGAAGAGTTGATTCGCCGTAAGCAAAAGGAAATCACTAAAGGGTTGGAGGAATTGGACACTGTGCAGTTCCGTGCAGACTCCTGGACCCGTGGTAACGATGGTGGTGGTGGTACCTCTATGGTTTTGCAAAACGGTTCTACTTTCGAAAAAGGTGGTGTCAATGTGAGTGTTGTTCACGGTACTTTGACTCCTCCAGCTATTAGAGCGATGAAAAACGATCACAAGAACTTGCACTTGCCAATTGACCCAGAAACTGGCGAGCCAGATGCTTCTGGTGTCAGATTTTTCGCTTGTGGTTTGTCGATGGTCATTCACCCAATCAACCCACACGCGCCAACAACGCATTTGAACTACAGATACTTCGAGACGATGCATGCGGACGGGACACCTCAGGCCTGGTGGTTTGGTGGTGGTGCCGATCTTACCCCATCGTATTTGTACGAGGAAGATGCAAAGTTGTTCCATCAATTGCACAAGGACGCTCTAGACAAGACAGACGTTACCCTATATCCAAAGTTCAAGAAATGGTGCGATGAGTACTTCTACATCAAGCACAGAGAAGAAACCAGAGGTATTGGTGGTATCTTTTTCGATGACTTGGACGACCGTGATCCAGACGTTTTGTTGAACATGGTCGAAAACTGTTTCGATGCGTTCTTGCCCTCTTACACGGAAATCATCAAGAGAAGAATGAACATGCCATACACCGAGGAGGAAAGACAATGGCAGCAGATTAGACGTGGTAGATACGTCGAATTCAACTTGGTCTTGGATAGAGGTACTCAATTTGGTTTGAGAACTCCTGGCTCTCGTGTTGAGAGTATCTTGATGTCCTTGCCTGTTACCGCCTCCTGGTTGTACGACCACCACCCTGAACCTGGCTCCAGAGAAGACAAATTGCTACAGGTCTTGAAAAACCCAATCGAATGGGTGTAATGGGTGCTTAAGATCTGTCTTTTTTATTTATAATCGGCCCTTTTATCACTTTATTTTTTAGTTTATTTATTACATCTTTCGTCTGTATACTAGTTTGGGTCAAAGAAGTCAAAAATCGCACAATTTGGGTGTAGAACGTAAACGTAGTTAACACCTTGCGCAGCCTCTGCATCAGCGTCTTCGAGATCTTGAACATCTTGTTCGTTGGGTGCAACTTTCATCAAGATCTTGTCATGTACCAATTTACTGAGAACCTTGTGCACCAACTTTCTTTCAGCCCAATATTCTTCCTCTGTATGAAGCTTGCCTTGTCCCCGCCGGGTCACCCGGCCAGCGACATGGAGGCCCAGAATACCCTCCTTGACAGTCTTGACGTGCGCAGCTCAGGGGCATGATGTGACTGTCGCCCGTACATTTAGCCCATACATCCCCATGTATAATCATTTGCATCCATACATTTTGATGGCCGCACGGCGCGAAGCAAAAATTACGGCTCCTCGCTGCGGACCTGCGAGCAGGGAAACGCTCCCCTCACAGACGCGTTGAATTGTCCCCACGCCGCGCCCCTGTAGAGAAATATAAAAGGTTAGGATTTGCCACTGAGGTTCTTCTTTCATATACTTCCTTTTAAAATCTTGCTAGGATACAGTTCTCACATCACATCCGAACATAAACAACCATGTTATTACCATTAACAAAGCTAAAACCGAGAGCAAAAGTTGCTGTTGTAGGGGGTGGCGTTTCTGGACTATGTTTTACTTATTTTTTAAGCAAGTTGAGACCGGATGTTGAAATCACACTGTTCGAATCGCAGAACAGAACTGGGGGTTGGATATATTCTTGTAACACAAGAGATATGAGTGGGAACCCAATTATGTTGGAGAAGGGACCCAGAACATTGAGGGGCGTATCAGACGGGACCGTTCTGATTATGGATACCCTTAAAGACTTGGGCAAGGAAGCAGTTATTCAAAGCATTGATAAAGGTTGCATTGCAGACAAAAAGTTTCTACTAGACCCCAGTGATAAACTCGTGCAGGTTCCTAATTCGATATCTACAACAGTAAAATTTCTGCTGAATCCGTTGGGAAAAGGACTCATCACGGGTATGATGGGAGAATGGTTCAGAAAAAAATCTCCACATCCTGGCCAAGACGAAAGTGTCGAATCCATTTGTGACAGAAGGTTTGGGAATAACTACATATCAAACAATATGATCAGTGCCTTACTAAGAGGTATCTATGGGGATGACGTTTCCCTATTAAGTGCCAAGAGAACGTTTAAGAAAATATACTACAATGAACTAAAGCACGGATCTAACACACAAGCTATGATTGATAATATGCGCGGAAAGTCAAGAAGTAAAAAAACTGAGAACCTGCACCAATCTTTAACTGGCTGCCTTAACGACTACTCAAATGCGTTTGGAAAAGACAGGTCAAAGTTATTAGACTTATCCAACACGCTAAAGAAATATCCCATGTTGGGTCTTGCTGGGGGACTAGAAACGTTTCCCAAGATAGTCAGAAATGCTTTGAACGAATTTAAAAACGTCAAAATAGTTACTGGGAACCCGGTTACGCAAATAATGAAACGCCCTGCTAACGAAACGACAATCGGATTGAAAGCGAAATCTGGCGACCAATACGAAACATTTGACCATTTAAGACTTACGATAACACCTCCCAAAATCGCTAAATTGCTACCGAAGGATCAAAATTCATTATCCAAGTTATTAGATGAGATACAATCAAACACAATAATTTTAGTTAATTATTATTTGCCAAACAAAGATGTAATAGATGCCGATCTACAAGGCTTTGGATACTTGGTTCCCAAATCCAATAAGAATCCAGGGAAATTGCTTGGTGTAATTTTCGATTCTGTTATCGAAAGGAATTTCAAACCACTTTTTGACAAACTCTCCACAAACCCAAACGCCCTCAACAAATATACAAAAGTGACTGCGATGATAGGAGGTTGTATGCTCAATGAACACGGTGTTCCTGTAGTGCCATCCAGGGAGGTAACCATTAATGCAGTCAAAGATGCGCTGAACAACCACCTCGGCATCAGTAACAAGGATCTGGAAGCTGGTCAGTGGGAATTCACTATCGCCGATAGATGTCTGCCAAGATTTCATGTAGGTTATGACGCATGGCAAGAAAGAGCTGAAAGGAAGTTGCAAGAATCTTACGGCCAAACAGTTTCTGTGGGGGGAATGGGATTTTCTAGAAGTCCCGGTGTCCCTGACGTTATTGTAGACGGCTTTAACGACGCCTTACAGCTAAGCAAATAATCAGTACTGACAATAAAAAGATTCTTGTTTTCAAGAACTTGTCATTTGTATAGTTTTTTTATATTGTAGTTGTTCTATTTTAATCAAATGTTAGCGTGATTTATATTTTTTTTCGCCTCGACATCATCTGCCCAGATGCGAAGTTAAGTGCGCAGAAAGTAATATCATGCGTCAATCGTATGTGAATGCTGGTCGCTATACTGCTGTCGATTCGATACTAACGCCGCCATCCAGTTTTTAGTTTGTTTAGAGATGGACCATGGACCATGGACGGTTAATGGTTTATATACAGCATGCACCCTTTGCCTTAAAACGGTAAAATGAGATAATGCCTTTCCGTTGTATCCCAGCCAGGCCAACCTCCCAGAGTATGTGCGGAGGGTAACGTCACGTAATGTAGAAAGTATTTCTGTATAGGTAGGGTACCCACCCGCATATGAAATTACTCTGTAGTTCCTGGGTCCCGTCGCCTCCGATATTCTTCTGCTTGATTTCTTTTCTGCCGTTTGCTAGTTTTCTTCAACCCCGGTTACGTTTATGCTTTTTCCTTTTGCTCTAGAAGACGGGTTTTTTTGCTCTTAACAAAGGCCAATCAATGCGATGGACTGGTCAATCTCGGCCGGACAGAGACAAACCCATCGTTCTTGACGATTGGGTTACAGTTCGCAGAGAGAAATGTAGTGGAATGGAAAGAGATCGCAGCAGTATATAGTCAGTTTATTCAGGTTACGCTATGGCATTTTTTTTTTTTGTTACGTAACTTGAGTACGGTGTTAGGTTTCTCTTTCTTTAGAAATTAGGGATGCTTCGGACAATTGGGGTTTTAGAAGAAGGGATACAAAGGAGTAGCATGCGTCGGGTGGTAGAGCTAATGTTAGCTGGTTGGGCTGGCCTGCCGGCTAGAATGGTTAATTGGTAGACCAGGTTTTTTTTTTTTCCCTTACTTCATTCCCTTACGTCTTTTGGGGCGACACAGGGTTTTGTATGAAGAGAATTGCATTTGCTATTAGTTAAGGCTTCTCCGAACCTGTATAAAAGTGGAGAGTTCGCAAGGTATGTAATTATATATACAGGATATATTTCCCTGCAATGCTAGTATTTAATGATGATAGACAGTTGGTTTTACAGGACAAAGATTCGTTTGGTCCATTGATTGAATAGCACACACTTCTGACTACGTAGTTGTTTAACAAGATCGAAGTGAATCTGACTCTAAGAATACAGTATACGAAACAATGCTTTCCAGAACAATCCGTACACAAGGTTCCTTCCTAAGAAGATCACAACTGACCATTACAAGATCATTTTCGGTTACATTCAACATGCAGAATGCACAAAAGAGATCACCCACAGGAATTGTTTTGATGAACATGGGTGGCCCCTCTAAAGTTGAGGAAACATATGATTTTTTGTATCAATTATTTGCCGATAATGACCTAATTCCCATTAGTGCTAAGTATCAGAAGACAATTGCTAAATATATTGCTAAGTTTCGTACCCCCAAGATAGAGAAGCAATATAGGGAAATTGGTGGGGGCTCCCCAATCCGGAAATGGTCTGAGTATCAAGCCACTGAGGTCTGTAAAATCTTAGATAAAACCTGTCCAGAAACGGCGCCTCATAAGCCTTACGTGGCGTTTCGTTACGCAAAGCCGCTAACCGCAGAAACTTATAAACAAATGCTAAAAGATGGCGTGAAGAAGGCAGTGGCCTTTTCTCAATATCCTCATTTCTCTTATTCCACTACCGGGTCATCCATCAACGAATTGTGGAGACAGATTAAGGCATTGGACTCCGAGAGATCTATATCTTGGTCGGTTATTGATCGTTGGCCTACAAATGAAGGTCTAATCAAGGCCTTCTCCGAAAATATCACCAAAAAACTACAAGAGTTTCCGCAACCTGTCAGAGACAAGGTTGTTTTATTGTTTTCCGCACATTCTCTACCCATGGATGTTGTTAACACCGGTGATGCCTACCCAGCTGAGGTAGCTGCGACGGTTTACAACATCATGCAAAAATTAAAGTTTAAAAACCCTTATAGGTTGGTTTGGCAATCCCAAGTTGGACCAAAACCATGGTTGGGAGCGCAGACAGCTGAAATTGCGGAATTTTTAGGCCCCAAAGTTGATGGCCTAATGTTTATTCCTATCGCCTTTACCTCTGATCATATTGAAACATTGCATGAAATTGACTTAGGCGTCATTGGGGAATCGGAATATAAGGATAAATTTAAGAGATGCGAATCTTTAAATGGCAACCAGACCTTTATTGAAGGCATGGCAGATCTCGTCAAAAGCCACTTACAGAGTAACCAACTCTATTCTAATCAACTACCTCTTGATTTTGCACTTGGCAAGTCCAATGATCCTGTAAAGGACCTTTCATTGGTATTTGGCAATCACGAATCTACTTGAAGATAAGACAGGGGAGAGGGATTCTTCTTCTTACCGACTCAACGCACCACACACCCTCTTTCCCAATTCTAATCATTCGATCTATGTAAACTTATCGTAACTAAACTAACTAACTACCAAGATTCTTCTTTACTTTACAGAGGTGTATTTCTTTAGTTGTCACATACAAAAACGCACACACACACACACATTTTGTCCTGTCCAATGACAAAATGTGACAGGAAACCCGAACGTCGGAGGTTTTCGGGTGGCAGTCATGTCGCCCAGTACTACTCGTAGCAGTAGTACTACTACCATTACACAAGGGCCAAACGTCAATGCTTTGCCGCGG

Table S3. Primers used in this study

| **Name** | **Sequence(5’-3’)** | **Application** |
| --- | --- | --- |
| LBA-F | ATGGTTGCTTTCACTGAAAAGCA | Amplify LBA |
| LBA-R | TTATGCTTTCTTAATAGCTGCTGCC |  |
| LBA-6His_6_-F | ATGGTTGCTTTCACTGAAAAGCAA | Amplify LBA-6His_6_ |
| LBA-6His_6_-R | TTAATGGTGATGGTGATGATGTGCTTTCTTAATAGCTGCT GCC |  |
| HEM1-F | ATGGAGTCTGTTGTTCGTCAATCTG | qPCR primer for detecting mRNA level of *HEM1_Km_* |
| HEM1-R | ACGCTGCTCCATAGCACGACC |  |
| HEM2-F | ATGGTTCACGTTGCAAAATTTTTAG | qPCR primer for detecting mRNA level of *HEM2_Km_* |
| HEM2-R | GTCACTAATGAATAGCGGGAAAATGA |  |
| HEM3-F | ATGGGAGAGAAGCGTATAATTCGTAT | qPCR primer for detecting mRNA level of *HEM3_Km_* |
| HEM3-R | TTGAACATGATCACCCAACGTGT |  |
| HEM4-F | ATGTCCAGAATAATTTTTCTTAAGAACAA | qPCR primer for detecting mRNA level of *HEM4_Km_* |
| HEM4-R | CCTGTTTCTGAATAGCGTCAACG |  |
| HEM12-F | ATGCAAGTACAAAATGTGGATCGTT | qPCR primer for detecting mRNA level of *HEM12_Km_* |
| HEM12-R | TTAACCTCATGATATTCAGGCAAGTAA |  |
| HEM13-F | ATGACAGACTCTGCTGTGCCAATG | qPCR primer for detecting mRNA level of *HEM13_Km_* |
| HEM13-R | GGTACCACCACCACCATCGTTAC |  |
| HEM14-F | ATGAATCCTACGGTATTAAAACTACCAGC | qPCR primer for detecting mRNA level of *HEM14_Km_* |
| HEM14-R | GCCACCACATCTATCTTTCGATTC |  |
| HEM15-F | ATGATTCGCCGTGAAGTAGGAAGT | qPCR primer for detecting mRNA level of *HEM15_Km_* |
| HEM15-R | GTCGTATGTCTCAGGCACCGTG |  |
| gltx-f | TCGCTAGTTATTGGTGCTAAGGCAC | qPCR primer for detecting mRNA level of *GLTX_Km_* |
| gltx-R | TTCAGTTTTGCCTTGGAAAGAGG |  |
| hem3_sc_-f | ATGGGCCCTGAAACTCTACATATTG | qPCR primer for detecting mRNA level of *HEM3_Sc_* |
| hem3_sc_-R | GAATTGAATCTGGTCACCAAGAGTTTG |  |
| hem12_sc_-f | CGATTTGATATTGAGAGCCGCAA | qPCR primer for detecting mRNA level of *HEM12_Sc_* |
| hem12_sc_-R | GAAGCAATTTCCGCATCCCTG |  |
| hem14_sc_-f | GACGGGACCGTTCTGATTATGGA | qPCR primer for detecting mRNA level of *HEM14_Sc_* |
| hem14_sc_-R | CCATCATACCCGTGATGAGTCCTT |  |
| hem15_sc_-f | ATGCTTTCCAGAACAATCCGTACACA | qPCR primer for detecting mRNA level of *HEM15_Sc_* |
| hem15_sc_-R | AGAGGGGCCACCCATGTTCATC |  |
| hemA_sty_-F | GCAAACTGGCGAACGCTTATCT | qPCR primer for detecting mRNA level of *HEMA_Sty_* |
| hemA_sty_-R | CACGAATCTGCTCCGACTGACTAC |  |
| hemL_Eco_-f | AATGGAAGTGAAAATGGCGCAAC | qPCR primer for detecting mRNA level of *HEML_Eco_* |
| hemL_Eco_-R | CCTTCAAATTTAATAATTTTGTCGCG |  |
| gRNA-LSC1-F | TCATTCTTGATGGTGCTGTCGTA | Deleting *LSC1* |
| gRNA-LSC1-R | AACTACGACAGCACCATCAAGAA |  |
| gRNA-LSC2-F | TCAGTCTCAACAATTCGGCTGAA | Deleting *LSC2* |
| gRNA-LSC2-R | AACTTCAGCCGAATTGTTGAGAC |  |
| gRNA-ROX1-F | TCACACTCTCTGCATCGTTCAGT | Deleting *ROX1* |
| gRNA-ROX1-R | AACACTGAACGATGCAGAGAGTG |  |
| gRNA-HAP1-F | TCATGCTGTGTGCCCGTGCTCCC | Deleting *HAP1* |
| gRNA-HAP1-R | aacGGGAGCACGGGCACACAGCA |  |
| gRNA-SSN3-F | GGTGATATCTAAAGATGACACCACT | Deleting *SSN3* |
| gRNA-SSN3-R | TTGTGTAAAGTTTCTCCTCTGATT |  |
| gRNA-TUP1-F | TCAACGAGTTAGAGTTGACGCAC | Deleting *TUP1* |
| gRNA-TUP1-R | AACGTGCGTCAACTCTAACTCGT |  |
| gRNA-VPS10-F | TCATTTGGCGGAATGCGGTTATA | Deleting *VPS10* |
| gRNA-VPS10-R | AACTATAACCGCATTCCGCCAAA |  |
| gRNA-PEP4-F | TCAACTGCGCATGTCTCTCGTTT | Deleting *PEP4* |
| gRNA-PEP4-R | AACAAACGAGAGACATGCGCAGT |  |
| gRNA-HIS3-F | ATCAGATAGCCATATCCTTACAT | Deleting *HIS3* |
| gRNA-HIS3-R | AACATGTAAGGATATGGCTATCT |  |
| gRNA-TRP1-F | ATCGATCGCGGCGCAGACCTGCT | Deleting *TRP1* |
| gRNA-TRP1-R | AACAGCAGGTCTGCGCCGCGATC |  |
